# Methods for Evaluation of medical prediction Models, Tests And Biomarkers (MEMTAB) 2020 Symposium: Virtual. 10-11 December 2020

**DOI:** 10.1186/s41512-021-00094-7

**Published:** 2021-04-01

## Keynotes and oral presentations MEMTAB2020

### List of abstracts (in order by programme schedule)

#### I1: Introductory extended abstract: MEMTAB2020

##### Jan Y Verbakel^1,2^, Ann Van den Bruel^1^ and Ben Van Calster^3,4^

###### ^1^Department of Public Health and Primary Care, KU Leuven, Leuven, Belgium; ^2^Nuffield Department of Primary Care Health Sciences, University of Oxford, UK; ^3^Department of Development and Regeneration, KU Leuven, Leuven, Belgium; ^4^Department of Biomedical Data Sciences, Leiden University Medical Center, Leiden, the Netherlands

####### Keynote session on PATH statement

This supplement contains the conference abstracts accepted at the MEMTAB2020 virtual symposium, hosted from Leuven, Belgium, focussing on Methods for Evaluation of Medical prediction Models, Tests And Biomarkers.

The MEMTAB2020 symposium was hosted by Professor Ben Van Calster, Professor Ann Van den Bruel, Professor Jan Verbakel and the University of Leuven's EPI-Centre, part of the Department of Public Health and Primary Care.

The international MEMTAB symposium attracts researchers, healthcare workers, policy makers and manufacturers actively involved in the development, evaluation or regulation of tests, (bio) markers, models, tools, apps, devices or any other modality used for the purpose of diagnosis, prognosis, risk stratification or (disease or therapy) monitoring.

Rapid technological progress coupled with the significant methodological complexities involved in developing, evaluating and implementing tests, markers, models or devices create formidable challenges. Yet these challenges are matched by unique opportunities, while the wide array of involved subdisciplines create an exciting milieu for the generation of new ideas and directions. The symposium aims to provide a forum for disseminating knowledge at the forefront of current research, and for stimulating dialogue that will propel future thought and endeavours to tackle the methodological and practical complexities facing the medical diagnostic, prognostic and monitoring field today. The virtual MEMTAB2020 event was specifically aimed at bringing together researchers from the diverse reaches of test evaluation, from in vitro test developers, industry and regulatory representatives, through methodologists, guideline developers and practising clinicians, in the hope of improving current understanding through knowledge exchange, and forging our diverse experiences and perspectives to delineate the future direction of diagnostic test research. In this respect, it is the only conference in the world that provides a platform dedicated to the investigation of medical tests, markers, models and other devices used for diagnosis prognosis and monitoring.

This year’s symposium focussed on the following conference themes:

- How to develop and apply prediction models and diagnostic tests
- High-dimensional data and genetic prediction
- Machine learning for evaluation of diagnostic tests, markers and prediction models
- Impact studies for diagnostic tests, markers and prediction models (including low resource settings)
- Systematic review and meta-analysis (including individual participant data)
- Big data, electronic health records, dynamic prediction
- How to quantify overdiagnosis

With over 135 delegates and 88 accepted abstracts, we believe we were able to offer a very strong programme.

It was our great pleasure to host this year’s symposium and are looking forward to meeting you again at the next MEMTAB symposium!

**Overview of the different committees**

***(listed alphabetically)***

**Conference Chairs:** Ben Van Calster, Ann Van den Bruel, Jan Y Verbakel

**Local organizing Committee:**

- Niel Hens, University of Antwerp and Hasselt University
- Ben Van Calster, KU Leuven and LUMC
- Ann Van den Bruel, KU Leuven
- Jan Y Verbakel, KU Leuven and University of Oxford

**Scientific Committee:**

- Gary Collins, University of Oxford
- Jon Deeks, University of Birmingham
- Nandini Dendukuri, McGill Universit
- Niel Hens, University of Antwerp and Hasselt University
- Lotty Hooft, UMC Utrecht
- Mariska Leeflang, AMC Amsterdam
- Richard Riley, Keele University
- Yemisi Takwoingi, University of Birmingham
- Maarten van Smeden, LUMC
- Laure Wynants, KU Leuven and Maastricht University
- Ben Van Calster, KU Leuven and LUMC
- Ann Van den Bruel, KU Leuven
- Jan Y Verbakel, KU Leuven and University of Oxford

### Session chair: Ben Van Calster

#### 1. Using Group Data for Individual Patients: The Predictive Approaches to Treatment Effect Heterogeneity (PATH) Statement

##### David M Kent^1^, David van Klaveren^1,2^, Jessica K. Paulus^1^ and Ewout Steyerberg^2,3^ for the PATH Group

###### ^1^Predictive Analytics and Comparative Effectiveness Center, Tufts Medical Center, Boston, MA, USA ^2^Department of Public Health, Erasmus University Medical Center, Rotterdam, The Netherlands; ^3^Department of Biomedical Data Sciences, Leiden University Medical Center, Leiden, The Netherlands

####### **Correspondence:** David M Kent

**Background:** Evidence-based medicine (EBM) relies on forecasting for an individual patient using the frequency of outcomes in groups of similar patients (i.e., a reference class) under alternative treatments. Despite a widespread belief that individuals respond differently to the same treatment, EBM has traditionally stressed the reference class of the whole trial population, in part because conventional (one-variable-at-a-time) subgroup analysis have well-known limitations.

We aimed to provide guidance for “predictive” approaches to heterogeneous treatment effects (HTE), which can provide patient-centered estimates of outcome risks under treatment alternatives, taking into account multiple relevant patient attributes simultaneously.

**Methods:** 1) a systematic literature review; 2) simulations to characterizing potential problems with predictive HTE analysis; and 3) a deliberative process engaging a technical expert panel to develop guidance.

**Results:** We found various limitations of conventional subgroup analysis in contrast to various advantages of predictive approaches. The latter span two broad classes: 1) Risk modeling, where patient subgroups are formed according to their risk of an outcome (usually the primary study outcome), exploiting the mathematical dependency of treatment effects on the control event rate; and 2) Effect modeling, where patients are disaggregated by a model developed directly from randomized trial data to predict treatment effects (i.e., contrasting outcome risks under two treatment conditions). Unlike risk modeling, effect modeling is “unblinded” to treatment assignment, allowing the inclusion of treatment-by-covariate interaction terms. We review strengths and limitations of these approaches and summarize recommendations in the PATH Statement.

**Conclusions:** While positive RCT results provide strong evidence that an intervention works for at least some patients, clinicians need to understand how a patient’s multiple characteristics combine to influence their potential treatment benefit. Revision and refinement of the PATH guidance supporting that goal is anticipated as experience with these novel methods grows.

**Keywords:** PATH statement, prediction, heterogeneity of treatment effect, personalized medicine

#### 2. Estimating heterogeneity of treatment effect by risk modeling

##### Ewout W. Steyerberg^1,2^, David M Kent^3^, David van Klaveren^1,3^

###### ^1^Department of Biomedical Data Sciences, Leiden University Medical Center, Leiden, The Netherlands; ^2^ Department of Public Health, Erasmus University Medical Center, Rotterdam, The Netherlands; ^3^ Predictive Analytics and Comparative Effectiveness Center, Tufts Medical Center, Boston, MA, USA

####### **Correspondence:** Ewout W. Steyerberg

**Background:** Heterogeneity of treatment effect refers to the nonrandom variation in the magnitude of the absolute treatment effect ('benefit') across levels of covariates. For randomized controlled trials (RCTs), the PATH (Predictive Approaches to Treatment effect Heterogeneity) Statement suggests 2 categories of predictive HTE approaches: “risk modeling” approaches, which combine a multivariable model with a constant relative effect of treatment, and “effect modeling” approaches, which includes interactions between treatment and baseline covariates^[1]^.

We aimed to assess practical challenges in deriving estimates of absolute benefit based on risk modeling.

**Methods & Results:** We re-analyzed data from 30,510 patients with an acute myocardial infarction, as enrolled in the GUSTO-I trial^[2]^. The average mortality was 6.3% with tPA and 7.3% with streptokinase, or an average benefit of 1.0% (p<.001). A multivariable logistic regression model included 6 predictors of 30-day mortality, which occurred in 2128 patients. The model provided a linear predictor (or risk score) that discriminated well between low risk and high risk patients, with an area under the ROC curve of 0.82. The benefit of tPA over streptokinase treatment increased from 0.2% to 2.4% for the lowest to the highest risk quarter (Figure 1). Proportionality of the treatment effect across predictors was not rejected in tests of interaction (overall test: p=0.30). Continuous benefit was estimated by subtracting estimated risk under either treatment with a spline transformation of the linear predictor (Figure 1). Sensitivity analyses showed similar results for different specifications of the risk model or the continuous benefit modeling. Exploratory one at a time subgroup analyses showed consistent relative effects of treatment.

**Conclusions:** Risk modeling should become part of the primary analysis of RCTs. One at a time subgroup analyses should be abandoned as secondary to indicate any heterogeneity of treatment effect.

**Fig. 1 (abstract 2).**
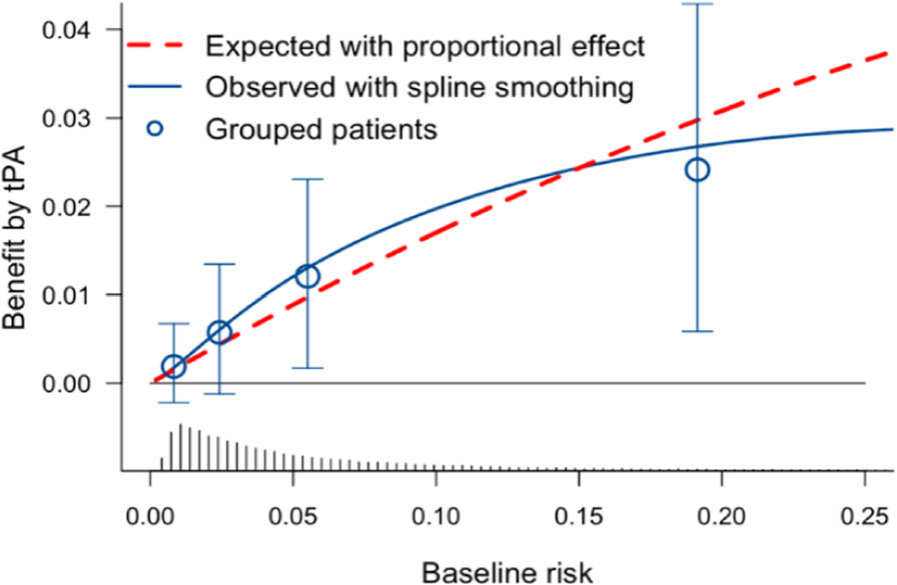
benefit of treatment by tPA compared to streptokinase in the GUSTO-I trial

**Keywords:** Heterogeneity of treatment effect, regression model, spline functions

**References**

^[1]^ David M. Kent, Jessica K. Paulus, David van Klaveren, et al. The Predictive Approaches to Treatment effect Heterogeneity (PATH) Statement. Ann Intern Med.2020;172:35-45.

## Linked contributed talks on predicting treatment response

### Session chair: Ben Van Calster

#### 3. Application of the PATH Statement: Predicting Treatment Benefit of Heart Bypass Surgery versus Coronary Stenting

##### David van Klaveren^1,2^, Kuniaki Takahashi^3^, Ewout W. Steyerberg^1,4^, David M. Kent^2^, Patrick W. Serruys^5^

###### ^1^Department of Public Health, Erasmus University Medical Center, Rotterdam, The Netherlands; ^2^Predictive Analytics and Comparative Effectiveness Center, Tufts Medical Center, Boston, MA, USA; ^3^Department of Cardiology, Academic Medical Center, University of Amsterdam, The Netherlands; ^4^Department of Biomedical Data Sciences, Leiden University Medical Center, Leiden, The Netherlands; ^5^Department of Cardiology, National University of Ireland, Galway, Ireland

####### **Correspondence:** David van Klaveren

**Background:** The Syntax Score II (SSII) was proposed to predict treatment benefit, i.e. the DIFFERENCE in 4-year mortality when treating complex Coronary Artery Disease patients with heart bypass surgery rather than coronary stenting. Between 4 and 10 years post-procedure, SSII has shown good predictive performance for mortality, but not for the treatment benefit of surgery versus stenting.

We aimed to develop a new SSII (SSII-2020) for predicting the treatment benefit of surgery versus stenting over a 10-year horizon.

**Methods:** Following the recently published PATH statement, we first used Cox regression in the SYNTAX trial data (n=1,800) to develop a clinical prognostic index (PI) for mortality over a 10-year horizon, blinded to treatment assignment. Second, we fitted a Cox model which included the treatment, the PI and 2 pre-specified effect-modifiers based on prior evidence: type of disease (Left Main Disease [LMD] or 3-Vessel Disease [3VD]), and anatomical disease complexity (SYNTAX Score [SS]). In a cross-validation, we assessed the ability of SSII-2020 to predict the absolute mortality difference between surgery and stenting.

**Results:** The PI consisted of 7 clinical predictors of mortality. SSII-2020 included the PI, treatment and 2 significant treatment interactions: surgery was on average beneficial for 3VD patients (HR 0.66; 95%CI 0.52-0.84), but not for LMD patients (HR 1.17; 95%CI 0.77-1.36; p-for-interaction 0.02), and the disease complexity only influenced mortality risk when patients were treated with stenting (HR per 10 SS points 1.17; 95%CI 1.06-1.30; p-for-interaction 0.05). In both treatment arms, SSII-2020 discriminated well (c-index 0.73) and was well calibrated for mortality risk. In contrast with SSII, SSII-2020 was well calibrated for treatment benefit both at 5 and 10 years post-procedure (Figure 1).

**Conclusions:** The newly developed SSII-2020 is able to predict the treatment benefit of heart bypass surgery versus coronary stenting over a 10-year horizon. External validation will be undertaken.

**Fig. 1 (abstract 3).**
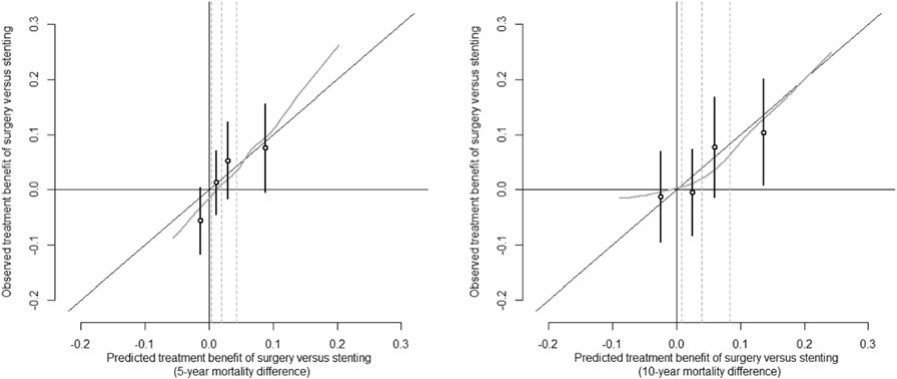
Calibration plots for benefit of treatment with surgery versus stenting at 5 years (left panel) and 10 years (right panel)

**Keywords:** PATH statement, prediction, heterogeneity of treatment effect, personalized medicine

#### 4. Individual participant data meta-analysis to examine treatment-covariate interactions: statistical recommendations for conduct and planning

##### Richard D. Riley^1^, Thomas Debray^2^, David Fisher^3^, Miriam Hattle^1^, Nadine Marlin^4^, Jeroen Hoogland^2^, Francois Gueyffier^5^, Jan A. Staessen^6^, Jiguang Wang^7^, Karel G.M. Moons^2^, Johannes B. Reitsma^2^, Joie Ensor^1^

###### ^1^Centre for Prognosis Research, School of Primary, Community and Social Care, Keele University, UK; ^2^Julius Center for Health Sciences and Primary Care, University Medical Center Utrecht, Utrecht, The Netherlands; ^3^MRC Clinical Trials Unit, Institute of Clinical Trials & Methodology, Faculty of Population Health Sciences, University College London, London, UK; ^4^Blizard Institute, Barts and The London School of Medicine and Dentistry, Queen Mary University of London, London, UK; ^5^Inserm, CIC201, Lyon, France; ^6^Studies Coordinating Centre, Research Unit Hypertension and Cardiovascular Epidemiology, KU Leuven Department of Cardiovascular Sciences, Leuven, Belgium; ^7^Centre for Epidemiological Studies and Clinical Trials, Ruijin Hospital, Shanghai Jiaotong University School of Medicine, Shanghai, China

####### **Correspondence:** Richard D. Riley

**Background:** Personalised healthcare often requires the use of treatment-covariate interactions, which refers to when a treatment effect (e.g. measured as a mean difference, odds ratio, hazard ratio) changes across values of a participant-level covariate (e.g. age, biomarker). Single randomised trials do not usually have sufficient power to detect genuine treatment-covariate interactions, which motivates the sharing of individual participant data (IPD) from multiple trials for meta-analysis. However, IPD meta-analyses are time consuming and statistically challenging

We aimed to provide statistical recommendations for conducting and planning an IPD meta-analysis of randomised trials to examine treatment-covariate interactions.

**Methods:** Drawing on our collective experience, we identify five key lessons to improve statistical analysis, and two key recommendations to improve planning IPD meta-analysis projects. Real IPD meta-analysis examples are used to substantiate the issues.

**Results:** For conduct, we recommend: (i) interactions should be estimated directly, and not by calculating differences in meta-analysis results for subgroups; (ii) interaction estimates should be based solely on within-study information; (iii) continuous covariates and outcomes should be analysed on their continuous scale; (iv) non-linear relationships should be examined for continuous covariates, using a multivariate meta-analysis; and (v) translation into clinical practice requires individualised treatment effect prediction. For planning, the decision to initiate an IPD meta-analysis should (a) not be based on between-study heterogeneity in the overall treatment effect; and (b) consider the potential power of an IPD meta-analysis conditional on characteristics of studies promising their IPD.

**Conclusions:** We hope our recommendations improve the planning and conduct of IPD meta-analyses to examine treatment-covariate interactions, to help flag when the approach is worthwhile and to ensure more robust results.^[1]^

**Keywords:** IPD meta-analysis, effect modifier, treatment-covariate interaction, subgroup

**References**

^[1]^ Riley RD, et al. IPD meta-analysis to examine interactions between treatment effect and participant-level covariates: statistical recommendations for conduct and planning. Stat Med (submitted)

## Contributed session on diagnostic tests

### Session chair: Nandini Dendukuri

#### 5. Test and Treat Superiority Plot: estimating threshold performance for developers of tests for treatment response

##### Neil Hawkins^1^, Janet Bouttell^1^, Andrew Briggs^2^, Dmitry Pomonomarev^3^

###### ^1^Health Economics and Health Technology Assessment, Institute of Health and Wellbeing, 1 Lilybank Gardens, Glasgow, Scotland; ^2^Department of Health Services Research and Policy, London School of Hygiene and Tropical Medicine, London, UK; ^3^Meshalkin National Medical Centre, Novosibirsk, Russian Federation

####### **Correspondence:** Neil Hawkins; Janet Bouttell

**Background:** It is useful for developers of diagnostic technologies to know how accurate a test predicting response to a treatment would need to be in order for a “test and treat” strategy to produce superior clinical outcomes to a ‘treat all’ strategy.

This study explored the derivation of a set of sensitivity and specificity values that define the threshold for clinical superiority for a test and treat strategy.

**Methods:** Taking the scenario of a test that predicts response to treatment A with a given sensitivity and specificity but does not predict the response to an alternative treatment (B), we developed a mathematical model that determined the threshold sensitivity and specificity required for a strategy of test and treat with “A” if positive or “B” if negative to outperform a treat-all strategy with either treatment A or B.

**Results:** We demonstrated that an estimate of odds ratio of response rate between treatments is sufficient to determine a set of threshold sensitivities and specificities for clinical superiority of the test and treat strategy. However, if the absolute probability of response is known for one of the treatments, the net-clinical benefit of the test can be estimated as a function of sensitivity and specificity. Using a hypothetical test of response to hormone treatment compared to chemotherapy in ovarian cancer, we demonstrate in a Shiny App the “Test and Treat Superiority Plot”, which illustrates the threshold performance necessary for a test of treatment response to outperform a treat all strategy given only the odds ratio between two treatments.

**Conclusions:** This model and plot can be used to distinguish promising candidate diagnostics from those unlikely to have clinical value. The plot also indicates how the relative importance of sensitivity and specificity varies as a function of the relative treatment effect.

**Keywords:** Diagnostic tests, sensitivity and specificity, test development

#### 6. Unblinded sample size re-estimation for diagnostic accuracy studies

##### Antonia Zapf^1^, Annika Hoyer^2^

###### ^1^Department of Medical Biometry and Epidemiology, University Medical Center Hamburg-Eppendorf, Hamburg, Germany; ^2^German Diabetes Center, Leibniz Center for Diabetes Research at Heinrich Heine University Düsseldorf, Institute for Biometrics and Epidemiology, Düsseldorf, Germany

####### **Correspondence:** Antonia Zapf

**Background:** In diagnostic accuracy studies, sensitivity and specificity are recommended as co-primary endpoints. For the sample size calculation, assumptions about the expected sensitivity and specificity of the index test as well as the minimal acceptable diagnostic accuracy or the expected diagnostic accuracy of the comparator test have to be made. However, the assumptions from previous studies are often unsure.^[1]^ As an example for the talk we chose the study from Yan et al., where the estimated sensitivity was 75.8%, whereas the authors expected 91%.^[2]^

**Methods:** Because of the uncertainty, it is essential to develop methods for a sample size re-estimation in diagnostic accuracy trials. While such adaptive designs are standard in interventional trials, in diagnostic trials they are uncommon.^[3]^ Known approaches from interventional trials cannot be applied to diagnostic accuracy studies or have to be modified; especially because the specific feature of diagnostic accuracy trials are the two co-primary endpoints sensitivity and specificity.

**Results:** In this talk we propose an approach for an unblinded sample size re-estimation in diagnostic accuracy studies. We can show that with the adaptive design the type-one error is maintained and the desired power is achieved. Furthermore, the results of the example study are presented.

**Conclusion:** Using unblinded sample size re-estimation, diagnostic accuracy studies can be made more efficient.

**Keywords:** Diagnostic accuracy, adaptive design, unblinded interim analysis

**References**

^[1]^ AW. Rutjes, JB. Reitsma, M. Di Nisio, N. Smidt, JC. van Rijn, PM. Bossuyt. CMAJ, 174(4) 2006, 469-476.

^[2]^ L. Yan, S. Tang, Y. Yang, X. Shi, Y. Ge, W. Sun, Y. Liu, X. Hao, X. Gui, H. Yin, Y. He, Q. Zhang. Medicine (Baltimore), 95(4) 2016, e2597.

^[3]^ A. Zapf, M. Stark, O. Gerke, C. Ehret, N. Benda, P. Bossuyt, J. Deeks, J. Reitsma, T. Alonzo, T. Friede. Stat Med [Epub ahead of print] 2019.

#### 7. An alternative method for presenting risk of bias assessments in systematic review of accuracy studies

##### Yasaman Vali^1^, Jenny Lee^1^, Patrick M. Bossuyt^1^, Mohammad Hadi Zafarmand^1^

###### ^1^Department of Clinical Epidemiology, Biostatistics & Bioinformatics, Amsterdam UMC, Amsterdam, The Netherlands

####### **Correspondence:** Yasaman Vali

**Background:** Systematic reviews include primary studies that differ in sample sizes, with larger studies contributing more to the meta-analysis. At present, study size is not considered in Risk of Bias evaluations.

We aimed to develop an alternative way to present the contribution of individual studies to the total body of evidence on diagnostic accuracy, in terms of risk of bias and concerns about applicability, one that takes the effective sample size into account.

**Methods:** We used the results of a systematic review of diagnostic accuracy studies of the Enhanced Liver Fibrosis (ELF) test for diagnosing liver fibrosis among non-alcoholic fatty liver disease patients. We assessed the 11 studies identified from our systematic search of five databases with the QUADAS-2 (Quality Assessment of Diagnostic Accuracy Studies) tool. We first used number of studies to show the proportion of studies at low, unclear and high risk of bias. We then developed an alternative version of the graph, which relies on the proportion of the total sample size of studies at different levels of risk of bias.

**Results:** The risk of bias levels for each domain of the QUADAS-2 checklist changed after replacing the number of studies with the relative sample sizes of the individual studies. For instance, the risk of bias was high in the patient selection domain in 45% of the studies, and low and unclear in 27% of studies (Figure 1A). The alternative graph using the sample sizes showed 25%, 41% and 34% of included population with high risk, unclear and low risk of bias, respectively (Figure 1B).

**Conclusion:** A fair representation of the risk-of-bias and concerns about applicability in the available body of evidence from diagnostic accuracy studies should be based on the total sample size, not on the number of studies

**Fig. 1 (abstract 7).**
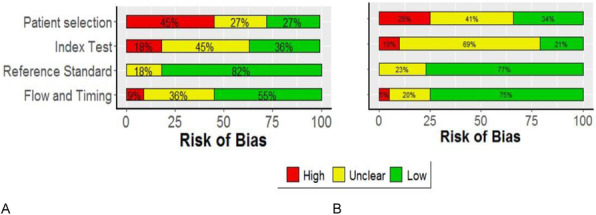
Risk of bias assessment results based on (A) proportion of included studies in the systematic review. (B) proportion of included patients in the systematic review

**Keywords:** Meta-analysis, accuracy studies, risk of bias assessment

#### 8. Major depression classification based on different diagnostic interviews: A synthesis of individual participant data meta-analyses

##### Yin Wu^1,2,3^, Brooke Levis^1,2,4^, John P. A. Ioannidis^5^, Andrea Benedetti^2,6^, Brett D Thombs^1,2,3^, and the DEPRESsion Screening Data (DEPRESSD) Collaboration^7^

###### ^1^Lady Davis Institute for Medical Research, Jewish General Hospital, Montréal, Québec, Canada; ^2^Department of Epidemiology, Biostatistics and Occupational Health, McGill University, Montréal, Québec, Canada; ^3^Department of Psychiatry, McGill University, Montréal, Québec, Canada; ^4^Centre for Prognosis Research, School of Primary, Community and Social Care, Keele University, Staffordshire, UK; ^5^Departments of Medicine, Health Research and Policy, Biomedical Data Science, and Statistics, Stanford University, Stanford, California, CA, USA; ^6^Respiratory Epidemiology and Clinical Research Unit, McGill University Health Centre, Montréal, Québec, Canada; ^7^McGill University, Montréal, Québec, Canada

####### **Correspondence:** Yin Wu

**Background:** Three previous individual participant data meta-analyses (IPDMAs) found that, compared to the semi-structured Structured Clinical Interview for DSM (SCID), the Composite International Diagnostic Interview (CIDI) and Mini International Neuropsychiatric Interview (MINI) tended to misclassify major depression.

We aimed to synthesize results from the three studies to compare the performance of the most commonly used diagnostic interviews for major depression classification: the SCID, CIDI, and MINI; and to determine if (1) probability of major depression classification based on the CIDI and MINI differs from probability based on SCID and (2) if differences are associated with depressive symptom levels.

**Methods:** We updated the Patient Health Questionnaire-9 IPDMA database, and standardised screening tool scores in all three databases. We re-analysed by fitting binomial generalized linear mixed models to compare odds of major depression classification across interviews, controlling for screening tool scores and participant characteristics, with and without an interaction term between interview and screening score. We synthesised results from these IPDMAs by estimating pooled adjusted odds ratios (aORs) for each interview and for interactions of each interview with screening scores using random effects meta-analysis.

**Results:** In total, 69,405 participants (7,574 [11%] with major depression) from 212 studies were included. The MINI (74 studies, 25,749 participants, 11% major depression) classified major depression more often than the SCID (108 studies, 21,953 participants, 14% major depression; aOR [95% CI] = 1.45 [1.11-1.92]). As screening scores increased, odds of major depression classification increased less for the CIDI (30 studies, 21,703 participants, 7% major depression) than the SCID (interaction aOR [95% CI] = 0.64 [0.52-0.80]).

**Conclusions:** Compared to the SCID, the MINI classifies major depression more often and the CIDI is less responsive to increases in symptom levels, regardless of measure of depressive symptom severity. Findings from research studies using MINI or CIDI should be cautiously interpreted.

**Keywords:** Depressive disorders, diagnostic interviews, individual participant data meta-analysis, major depression

#### 9. What makes a good cancer biomarker? Developing a consensus

##### Katerina-Vanessa Savva^1^, Melody Ni^1^, George B. Hanna^1^, and Christopher J. Peters^1^

###### ^1^Department of Surgery and Cancer, Imperial College London, London, UK

####### **Correspondence:** Katerina-Vanessa Savva

**Background:** Although a large number of resources have been invested in biomarker (BM) discovery, for both prognostic and diagnostic purposes, very few of those BMs have been clinically adopted. In an attempt to bridge the gap between BM discovery and clinical use, our previous study has developed and retrospectively validated a checklist comprised of 125 characteristics associated with cancer BM clinical implementation. Despite validation, complexity in implementing the full checklist might present a barrier. Therefore, this study aims to generate a user-friendly and concise consensus statement with literature-reported attributes associated with successful BM implementation.

**Methods:** A checklist of BM attributes was created using Medline and Embase databases according to PRISMA guidelines. A qualitative approach was applied to validate the list utilising semi-structured interviews (n=32). Thematic analysis was conducted until thematic saturation was achieved. Upon completion of literature review and interviews, a 3-phase online Delphi-Survey was designed aiming to develop a consensus document. The participants involved were grouped based on their expertise: clinicians, academics, patient and industry representatives.

**Results:** Previously identified 125 attributes retrieved from literature and reporting guidelines were included in the checklist. Upon thematic analysis of the interviews, characteristics listed in the checklist were validated. Most commonly occurring theme focused on clinical utility. Interestingly, different groups focused on differential themes emphasising the importance of participants’ diverse background. In specific, clinician and laboratory personnel commonly occurring themes fell under clinical utility. Moreover, patient representatives and industry personnel recurrent themes focused on clinical and analytical validity, respectively.

**Conclusions:** This study generated a validated checklist with literature-reported attributes linked with successful BM implementation. Upon completion of the Delphi-survey, a consensus statement will be generated which could be used to i) detect BMs with the highest potential of being clinically implemented and ii) shape how BM studies are designed and performed.

**Keywords:** Biomarkers, clinical implementation, checklist, Delphi survey, qualitative research

#### 10. Developing Target Product Profiles for medical tests: a methodology review

##### Paola Cocco^1^, Anam Ayaz-Shah^2^, Michael Paul Messenger,^3^ Robert Michael West,^4^ Bethany Shinkins^1^

###### ^1^Test Evaluation Group, Academic Unit of Health Economics, University of Leeds, Leeds, UK; ^2^Academic Unit of Primary Care, Leeds Institute for Health Sciences, University of Leeds, Leeds, UK; ^3^Centre for Personalised Health and Medicine, University of Leeds, Leeds, UK; ^4^Leeds Institute for Health Sciences, University of Leeds, Leeds, UK

####### **Correspondence:** Paola Cocco

**Background:** A Target Product Profile (TPP) is a strategic document which describes the necessary characteristics of an innovative product to address an unmet clinical need. TPPs present valuable information for designing ‘fit for purpose’ tests to manufacturers. To our knowledge, there is no formal guidance as to best practice methods for developing a TPP specific to medical tests.

We aimed to review and summarise the methods currently used to develop TPPs for medical tests and identify the test characteristics commonly reported.

**Methods:** We conducted a methodology systematic review of TPPs for medical tests. Database and website searches were carried out in November 2018. TPPs written in English for any medical test were included. Test characteristics were clustered into commonly recognized themes.

**Results:** Forty-four studies were identified, all of which focused on diagnostic tests for infectious diseases. Three core decision-making phases for developing TPPs were identified: scoping, drafting and consensus-building. Consultations with experts and the literature mostly informed the scoping and drafting of TPPs. All TPPs provided information on unmet clinical need and desirable test analytical performance, and the majority specified clinical validity characteristics. Few TPPs described specifications for clinical utility, and none included cost-effectiveness.

**Conclusions:** Based on our descriptive summary of the methods implemented, we have identified a commonly used framework that could be beneficial for anyone interested in drafting a TPP for a medical test. We also highlighted some key weaknesses, including the quality of the information sources underpinning TPPs and failure to consider test characteristics relating to clinical utility and cost-effectiveness. This review provides some recommendations for further methodological research on the development of TPPs for medical test. This work would also help to inform the development of a formal guideline on how to draft TPPs for medical tests.

**Keywords:** Medical test, target product profile, TPP, quality by design, diagnostic, test characteristic

#### 11. Nonparametric Limits of Agreement for small to moderate sample sizes - a simulation study

##### Maria E Frey^1^, Hans Christian Petersen^2^, Oke Gerke^3^

###### ^1^Department of Toxicology, Charles River Laboratories Copenhagen A/S , Hestehavevej 36 A, 4623 Lille Skensved, Denmark; ^2^Department of Mathematics and Computer Science, University of Southern Denmark, Campusvej 55, 5230 Odense M, Denmark; ^3^Department of Clinical Research, University of Southern Denmark & Department of Nuclear Medicine, Odense University Hospital, Kløvervænget 47, 5000 Odense C, Denmark

####### **Correspondence:** Oke Gerke

**Background:** The assessment of agreement in method comparison and observer variability analysis on quantitative measurements is often done with Bland-Altman Limits of Agreement (BA LoA) for which the paired differences are implicitly assumed to follow a Normal distribution. Whenever this assumption does not hold, the respective 2.5% and 97.5% percentiles are often assessed by simple quantile estimation.

Sample, subsampling, and Kernel quantile estimators as well as other methods for quantile estimation have been proposed in the literature and were compared in this simulation study.

**Methods:** Given sample sizes between 30 and 150 and different distributions of the paired differences (Normal; Normal with 1%, 2%, and 5% outliers; Exponential; Lognormal), the performance of 14 estimators in generating prediction intervals for one newly generated observation was evaluated by their respective coverage probability.

**Results:** For n=30, the most simple sample quantile estimator (smallest and largest observation as estimates for the 2.5% and 97.5% percentiles) outperformed all other estimators. For sample sizes of n=50, 80, 100, and 150, only one other sample quantile estimator (a weighted average of two order statistics) complied with the nominal 95% level in all distributional scenarios. The Harrell-Davis subsampling estimator and estimators of the Sfakianakis-Verginis type achieved at least 95% coverage for all investigated distributions for sample sizes of at least n=80 apart from the Exponential distribution (at least 94%).

**Conclusions:** Simple sample quantile estimators based on one and two order statistics can be used for deriving nonparametric Limits of Agreement. For sample sizes exceeding 80 observations, more advanced quantile estimators of the Harrell-Davis and Sfakianakis-Verginis types that make use of all observed differences are equally applicable, but may be considered intuitively more appealing than simple sample quantile estimators that are based on only two observations per quantile (Figure 1).

**Fig. 1 (abstract 11).**
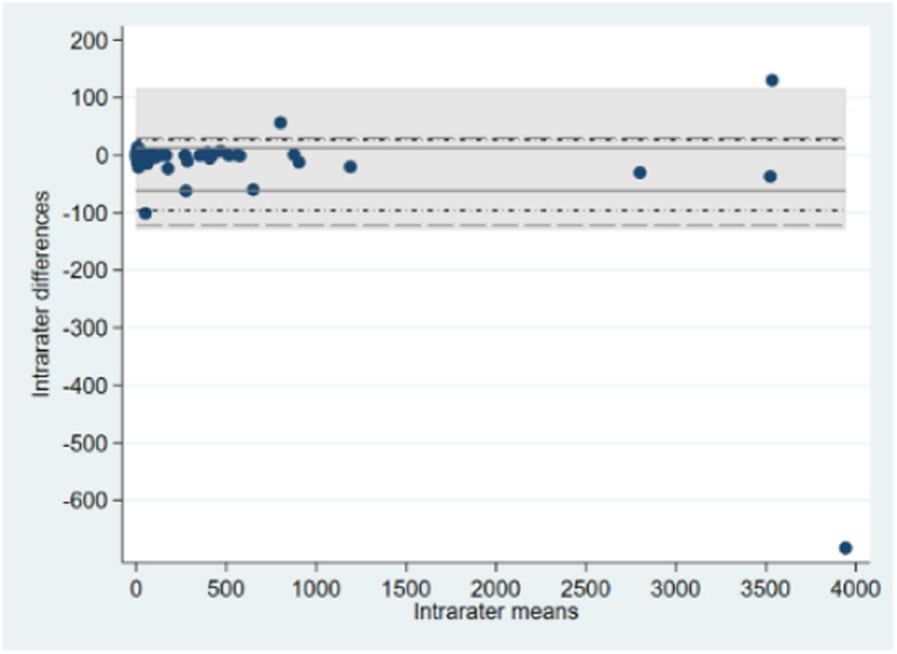
A sample quantile estimator (weighted average of two order statistics; solid), Harrell-Davis subsampling estimator (short dashes and dots), and an estimator of Sfakianakis-Verginis type (long dashes) contrasted with classical BA LoA (shaded area); n=129.

**Keywords:** Agreement, Bland-Altman plot, coverage, prediction, quantile estimation, repeatability, reproducibility

## Contributed session on prediction models

### Session chair: Laure Wynants

#### 12. QUADAS-C: a tool for assessing risk of bias in comparative diagnostic accuracy studies

##### Bada Yang^1^, Penny Whiting^2^, Clare Davenport^3,4^, Jonathan Deeks^3,4^, Christopher Hyde^5^, Susan Mallett^3^, Yemisi Takwoingi^3,4^ and Mariska Leeflang^1^ for the QUADAS-C group

###### ^1^Department of Clinical Epidemiology, Biostatistics and Bioinformatics, Amsterdam UMC, University of Amsterdam, Meibergdreef 9, 1105AZ, Amsterdam, The Netherlands; ^2^Population Health Sciences, Bristol Medical School, Canynge Hall, 39 Whatley Road, Bristol BS8 2PS, UK; ^3^Test Evaluation Research Group, Institute of Applied Health Research, University of Birmingham, Edgbaston, Birmingham, B15 2TT, UK; ^4^NIHR Birmingham Biomedical Research Centre, University Hospitals Birmingham NHS Foundation Trust and University of Birmingham, Birmingham, UK; ^5^Exeter Test Group, Institute of Health Research, College of Medicine and Health, University of Exeter, Exeter, UK

####### **Correspondence:** Bada Yang

**Background:** Comparative diagnostic test accuracy studies assess the accuracy of multiple tests in the same study and compare their accuracy. While these studies have the potential to yield reliable evidence regarding comparative accuracy, shortcomings in the design, conduct and analysis may bias their results. The currently recommended quality assessment tool for diagnostic accuracy studies, QUADAS-2, is not designed for the assessment of test comparisons.

We developed QUADAS-C as an extension to QUADAS-2 to assess the risk of bias in comparative diagnostic test accuracy studies.

**Methods:** Through a four-round Delphi study involving 24 international experts in test evaluation and a face-to-face consensus meeting, we developed a draft version of QUADAS-C which will undergo piloting in ongoing systematic reviews of comparative diagnostic test accuracy.

**Results:** QUADAS-C retains the same four-domain structure of QUADAS-2 (patient selection, index test, reference standard, flow and timing) and is comprised of additional questions to each QUADAS-2 domain. A risk of bias judgement for comparative accuracy requires a risk of bias judgement for each test (QUADAS-2), and additional criteria specific for test comparisons. Examples of such additional criteria include whether patients either received all index tests or were randomized to index tests, and whether index tests were interpreted blinded to other index tests.

**Conclusions:** QUADAS-C will be useful for systematic reviews of diagnostic test accuracy addressing comparative accuracy questions. Furthermore, researchers may use this tool to identify and avoid risk of bias when designing a comparative diagnostic test accuracy study. Currently a draft version of QUADAS-C is being piloted and the tool will be finalized by the time of the conference.

**Keywords:** Diagnostic accuracy, bias, test comparison, methodology, systematic review

#### 13. Minimum sample size for external validation of a clinical prediction model with a continuous outcome

##### Archer L^1^, Snell KIE^1^, Ensor J^1^, Hudda M^2^, Collins GS^3^, Riley RD^1^

###### ^1^Centre for Prognosis Research, School of Primary, Community and Social Care. Keele University, Staffordshire, UK; ^2^Population Health Research Institute, St George’s, University of London, London, UK; ^3^Centre for Statistics in Medicine, Nuffield Department of Orthopaedics, Rheumatology and Musculoskeletal Sciences, University of Oxford, Oxford, UK

####### **Correspondence:** Archer L

**Background:** Once a clinical prediction model has been developed its predictive performance should be examined in new data, independent to that used for model development. This process is known as external validation. Many current external validation studies suffer from small sample sizes and, subsequently, imprecise estimates of a model’s predictive performance.

To address this, in our talk we propose methods to determine the minimum sample size needed for external validation of a clinical prediction model with a continuous outcome.

**Methods:** Four criteria are proposed, that target precise estimates of (i) *R*^2^ (the proportion of variance explained), (ii) calibration-in-the-large (agreement between predicted and observed outcome values on average), (iii) calibration slope (agreement between predicted and observed values across the range of predicted values), and (iv) the variance of observed outcome values. Closed-form sample size solutions are derived for each criterion, which require the user to specify anticipated values of the model’s performance (in particular *R*^2^) and the outcome variance.

**Results:** The sample size formulae require the user to specify their desired precision for each performance estimate, whilst also making assumptions about the anticipated distribution of predicted values and the expected model performance in the validation study. For the latter, a sensible starting point is to base values on those reported in the model development study, assuming the target population is similar. The largest sample size required to meet all four criteria is the recommended minimum sample size needed in the external validation dataset. We illustrate the proposed methods on a case-study predicting fat-free mass in children, with the criteria suggesting a sample size of at least 234 participants are needed.

**Conclusion:** We recommend that researchers consider the minimum sample size required to precisely estimate key predictive performance measures, before commencing external validation of a prediction model for a continuous outcome.

**Keywords:** Sample size, external validation, prediction model, continuous outcome

#### 14. A systematic review of clinical prediction models developed using machine learning methods in Oncology

##### Paula Dhiman^1^, Jie Ma^1^, Benjamin Speich^1^, Garrett Bullock^2^, Constanza Andaur-Navarro^3^, Shona Kirtley^1^, Ben Van Calster^4^, Richard Riley^5^, Karel G Moons^3^, Gary S Collins^1^

###### ^1^Centre for Statistics in Medicine, University of Oxford, Oxford, UK; ^2^Nuffield Department of Orthopaedics, Rheumatology and Musculoskeletal Sciences, University of Oxford, Oxford, UK; ^3^Julius Center, UMC Utrecht, Utrecht University, Utrecht, The Netherlands; ^4^Department of Development and Regeneration, KU Leuven, Leuven, Belgium; ^5^School of Primary, Community and Social Care, University of Keele, Keele, UK

####### **Correspondence:** Paula Dhiman

**Background:** Clinical prediction models (CPMs) are of great interest to Oncology clinicians, who can use past and current patient characteristics to inform current and future health status. However, systematic reviews show that CPMs are often developed and validated using inappropriate methodology and are poorly reported. Application of Machine Learning (ML) methods to develop CPMs has risen considerably and it is often portrayed to offer many advantages (over traditional statistical methods), especially when using ‘big’, non-linear and high-dimensional data. However, poor methodology and reporting continue to be barriers to their clinical use. To improve usability of ML-CPMs, it is important to evaluate their methodological quality and adherence to reporting guidelines for prediction modelling.

We aimed to evaluate methodological conduct and reporting of author-defined ML-CPM studies within Oncology.

**Methods:** We conducted a systematic review of Oncology ML-CPMs published in 2019 using MEDLINE and Embase. We excluded studies using imaging or lab-based data. We extracted data on study design, outcome, sample size, ML methodology, and items for risk of bias^[1]^. The primary outcome was adherence to prediction modelling reporting guidelines^[2]^.

**Results:** We identified 2922 publications and excluded 2843 based on the eligibility criteria; extracting data from 79 publications. Preliminary results show poor reporting and methodological conduct. Studies used inefficient validation methods (e.g., split-sample) and did not adequately address missing data. Sample size was not reported for most studies, and discrimination was emphasised over calibration. Studies were at increased risk of overfitting, leading to optimistic performance measures for their models.

**Conclusions:** Reporting and methodological conduct of Oncology ML-CPMs needs to be improved. Caution is needed when interpreting ML-CPMs as performance may be over-optimistic.

**Keywords:** Machine learning, prediction modelling, reporting

**References**

^[1]^ Wolff RF, Moons KGM, et al, *Annals of Internal Medicine*, 170 **2019**, 51-58.

^[2]^ Heus P, Damen JAAG, et al, *BMJ Open*, 9 **2019**, e025611.

#### 15. Causal interpretation of clinical prediction models: When, why and how

##### Matthew Sperrin^1^, Lijing Lin^1^, David Jenkins^1^, Niels Peek^1^

###### ^1^Health e-Research Centre, Division of Informatics, Imaging and Data Science, University of Manchester, UK

####### **Correspondence:** Matthew Sperrin

**Background:** When developing models for prediction, neither the parameters of the model, nor the output predictions, have any causal interpretations. For pure prediction this is perfectly acceptable. However, prediction models are commonly interpreted in a causal manner - for example by altering inputs to the model to demonstrate hypothetical impact of an intervention. This can lead to biased causal effects being inferred, and thus misinformed decision making.

We aimed to collect examples of use of prediction models in a causal manner in practice, and to identify and interpret literature that provides methods for enriching prediction models with causal interpretations.

**Methods:** We systematically reviewed literature to identify methods for prediction models with causal interpretations, by adapting a scoping review framework, and considering the interaction of prediction modelling keywords, and causal inference keywords. We included papers where methods are developed or applied that undertake prediction enriched with causal inference methods; specifically allowing for some assessment of the causal impact of an intervention on predicted risk.

**Results:** There were two broad categories of approach identified: 1) enriching prediction models with externally estimated causal effects, such as from meta-analyses of clinical trials; and 2) estimating both a prediction model, and causal effects, from observational data. The latter category included methods such as marginal structural models and g-estimation, embedded within both statistical and machine learning frameworks.

**Conclusions:** There is a need for prediction models that allow for 'counterfactual prediction': i.e. estimating risk of outcomes under different hypothetical interventions, to support decision making. Methods exist but require development, particularly when triangulating data from different sources (e.g. observational data and randomised controlled trials). Techniques are also required to validate such models.

**Keywords**: Causal, counterfactual, prediction, model

#### 16. Risk prediction with discrete ordinal outcomes; calibration and the impact of the proportional odds assumption

##### Michael Edlinger^1,2^, Maarten van Smeden^3,4^, Hannes F Alber^5,6^, Ewout W Steyerberg^7^, Ben Van Calster^1,7^

###### ^1^Department of Development and Regeneration, KU Leuven, Leuven, Belgium; ^2^Department of Medical Statistics, Informatics, and Health Economy, Medical University Innsbruck, Innsbruck, Austria; ^3^Julius Center for Health Science and Primary Care, University Medical Center Utrecht, Utrecht, the Netherlands; ^4^Department of Clinical Epidemiology, Leiden University Medical Center, Leiden, the Netherlands; ^5^Department of Internal Medicine and Cardiology, Klinikum Klagenfurt am Wörthersee, Klagenfurt, Austria; ^6^Karl Landsteiner Institute for Interdisciplinary Science, Rehabilitation Centre, Münster, Austria; ^7^Department of Biomedical Data Sciences, Leiden University Medical Center, Leiden, the Netherlands

####### **Correspondence:** Michael Edlinger

**Background:** When evaluating the performance of risk prediction models, calibration is often underappreciated. There is little research on calibration for discrete ordinal outcomes.

We aimed to compare calibration measures for risk models that predict a discrete ordinal outcome (typically 3 to 6 categories), investigate the impact of assuming proportional odds on risk estimates and calibration, and study the impact of assuming proportional odds.

**Methods:** We studied multinomial logistic, cumulative logit, adjacent category logit, continuation ratio logit, and stereotype logistic models. To assess calibration, we investigated calibration intercepts and slopes for every outcome level, for every dichotomised version of the outcome, and for every linear predictor (i.e. algorithm-specific calibration). Finally, we used the estimated calibration index as a single-number metric, and constructed calibration plots. We used large sample simulations to study the behaviour of the logistic models in terms of risk estimates, and small sample simulations to study overfitting. As a case study, we used data from 4,888 symptomatic patients to predict the degree of coronary artery disease (five levels, from no disease to three-vessel disease).

**Results:** Models assuming proportional odds easily resulted in incorrect risk estimates. Calibration slopes for specific outcome levels or for dichotomised outcomes often deviated from unity, even on the development data. Non-proportional odds models, however, suffered more from overfitting, because these models require more parameters. Algorithm-specific calibration for proportional odds models assumes that this assumption holds, and therefore did not fully evaluate calibration.

**Conclusions:** Deviations from the proportional odds assumption can result in poor risk estimates and calibration. Therefore, non-proportional odds models are generally recommended for risk prediction, although larger sample sizes are needed.

**Keywords**: Prediction, calibration, ordinal outcome, proportional odds

#### 17. Recovering the full equation of an incompletely reported logistic regression model

##### Toshihiko Takada^1^, Chris van Lieshout^1^, Jeroen Hoogland^1^, Ewoud Schuit^1^, Johannes B Reitsma^1^

###### ^1^Julius Center for Health Sciences and Primary Care, University Medical Center Utrecht, Utrecht University, Universiteitsweg 100, 3584 CG Utrecht, The Netherlands

####### **Correspondence:** Toshihiko Takada

**Background:** Reporting of clinical prediction models has been shown to be poor with information on the intercept often missing. To allow application of a model for individualized risk prediction, information on the intercept is essential.

We aimed to evaluate possible methods to estimate an unreported intercept of a logistic regression model.

**Methods:** Using existing data, we developed a logistic regression model with 6 predictors to predict the risk of operative delivery in pregnant women. We considered 4 scenarios which did not report the intercept, but in which different information was available: i) web calculator, ii) nomogram, iii) coefficients/odds ratios, and iv) scoring table (i.e., three simplified categories and corresponding predicted probabilities). In scenario i) and ii), the coefficient for each predictor was estimated by assessment of the change in predicted probabilities that occurred with the change in the particular predictor. Then, the intercept was estimated by calculating the differences between the predicted probability and the estimated predictor coefficients. In scenario iii), the intercept was estimated based on the assumption that the mean risk estimated by the model would be close to the observed incidence of the outcome in a patient who had the mean value for each predictor. In scenario iv), the intercept was estimated by the association between score categories and corresponding predicted probabilities.

**Results:** Among 5667 laboring women, 1590 (28.1%) had an operative delivery. While the true value of the intercept was -9.563, the estimated intercept in each scenario was -9.552, -9.580, -9.308, and -8.940, respectively.

**Conclusion:** In scenarios i) and ii) where detailed information of predicted probability is available, the unreported intercept can be accurately estimated. On the other hand, the estimation of the intercept could be unstable when only coefficients/odds ratios or simple scoring and corresponding predicted probabilities are reported.

**Keywords:** Intercept, prediction model, logistic regression

#### 18. AI phone apps for skin cancer: Reviewing the evidence, regulations, marketing, plus what happened next

##### Jon Deeks^1,2^, Jac Dinnes^1,2^, Karoline Freeman^1,3^, Naomi Chuchu^1,4^, Sue E Bayliss^1^, Rubeta N Matin^5^, Abhilash Jain^6,7^, Yemisi Takwoingi^1,2^, Fiona Walter^8^, Hywel Williams^9^

###### ^1^Test Evaluation Research Group, University of Birmingham, Birmingham B30 1UZ UK; ^2^NIHR Biomedical Research Centre, University Hospitals Birmingham NHS Foundation Trust and University of Birmingham, Birmingham UK; ^3^Warwick Medical School, University of Warwick, Coventry, UK; ^4^London School of Hygiene and Tropical Medicine, London UK; ^5^Department of Dermatology, Churchill Hospital, Oxford UK; ^6^Nuffield Department of Orthopaedics, Rheumatology and Musculoskeletal Sciences, University of Oxford, Oxford UK; ^7^Department of Plastic and Reconstructive Surgery, Imperial College Healthcare NHS Trust, St Mary's Hospital, London UK; ^8^Department of Public Health and Primary Care, University of Cambridge, Cambridge UK; ^9^Centre for Evidence Based Dermatology, University of Nottingham, Nottingham UK

####### **Correspondence:** Jon Deeks

**Background:** AI based smartphone diagnostic apps can empower app users to make risk assessments and diagnoses. Apps for assessing suspicious moles for skin cancer are being implemented in health systems in the UK and elsewhere.

As a case study of AI, we examined the validity and findings of studies of AI smartphone apps to assess suspicious lesions for skin cancer, and assessed regulatory and NHS implementation processes.

**Methods:** We searched eight major databases and registers, including accuracy studies of any design. Reference standards included histological diagnosis and follow-up, or expert assessment. QUADAS-2 assessments summarised weaknesses in the evidence. Consequently we investigated how evidence is used with the MHRA and the NHS.

**Results:** Nine small studies evaluating six smartphone apps were found, two further studies have been provided since. QUADAS-2 assessments showed studies at high risk of bias with selective recruitment, unevaluable images, and differential verification. Applicability concerns included recruitment in secondary care clinics; clinicians performing lesion selection and image acquisition, and commercial confidentiality, name changes, and no version identifiers preventing app identification. Sensitivity estimates improved over time, but specificity remained below 80%. Two apps have CE marks as Class 1 devices. The Intended Use Statements state apps should not be used instead of medical assessments. NHS marketing material did not report specificity values, implied favorable comparisons with health professionals, and was contrary to the Intended Use statement. The NHS AI implementation process does not require a formal independent assessment of evidence.

**Conclusions:** Evidence does not support use of smartphone apps to detect melanoma. Current marketing of one app is contrary to its authorisation, and there is serious risk of harm to the public as a result. We have reported our findings to the NHS and the regulators and will update on what happened next.

**Keywords:** AI, apps, evidence, regulation

#### 19. TRIPOD-CLUSTER: reporting of prediction model studies in IPD-MA, EHR and other clustered datasets

##### Thomas P. A. Debray^1^, Kym Snell^2^, Ben van Calster^3^, Gary S. Collins^4^, Richard D. Riley^2^, Johannes B. Reitsma^1^, Doug G. Altman^4^, Karel G. Moons^1^

###### ^1^Julius center for Health Sciences and Primary Care, University Medical Center Utrecht, Utrecht University, The Netherlands; ^2^Research Institute for Primary Care and Health Sciences, Keele University, Staffordshire, UK; ^3^Department of Development and Regeneration, KU Leuven, Leuven, Belgium; ^4^Centre for Statistics in Medicine, Nuffield Department of Orthopaedics, Rheumatology & Musculoskeletal Sciences, Botnar Research Centre, University of Oxford, Oxford, UK

####### **Correspondence:** Thomas P. A. Debray

**Background:** The TRIPOD (Transparent Reporting of a multivariable prediction model for Individual Prognosis Or Diagnosis) Statement is a widely acknowledged guideline for the reporting of studies developing, validating, or updating a prediction model. With increasing availability of large datasets (or “big data”) from electronic health records and from individual participant data (IPD) meta-analysis, authors face novel opportunities and challenges for conducting and reporting their prediction model research. In particular, when prediction model development and validation studies include participants from multiple clusters such as multiple centers or studies, prediction models may generalize better across multiple centers, settings and populations. However, differences in participant case-mix (e.g. in participant eligibility criteria), in variable definitions and in data quality may also lead to substantial variation in model performance. Accordingly, prediction model studies that are based on large clustered datasets need to be sufficiently reported to determine whether a developed or validated prediction model is fit for purpose.

**Methods:** We describe the rationale and development process of an extension of TRIPOD focusing on studies aimed at developing, validating or updating a prediction model that use large clustered datasets with IPD from multiple studies or datasets with data combined from multiple centers, regions or countries.

**Results:** We pay specific attention to new items (N=10) and those TRIPOD items that required adjustment (N=20) due to the fact that participants are clustered within studies, practices, regions or countries.

**Conclusions:** The rationale for each items is discussed, along with examples of good reporting and why transparent reporting is important, with a view to assessing risk of bias and clinical usefulness of the developed or validated prediction model.

**Keywords:** Prediction, model, reporting, meta-analysis, individual participant data

## Invited talk: Rudi Pauwels

### Session chair: Ann Van den Bruel

#### 20. High Impact Pandemics: From Crisis to Preparedness

##### Rudi Pauwels^1^

###### ^1^Praesens Foundation, Zemst, Belgium

####### Keynote: Cecile Janssens

**Background:** The ongoing Covid-19 pandemic was just a matter of time to occur, given not only the long history of outbreaks and pandemics but also the increase in their frequency and diversity during the past decades. A number of human and non-human related factors are converging and driving these outbreaks.

The causative viral agent, SARS-Co-2, spread around the world in a matter of weeks, facilitated by transmission via the respiratory airways and even by people who do not display symptoms.

The world still had to predominantly react in crisis mode, exposing gaps in pandemic preparedness at multiple fronts. It's a reminder that a problem in one part of the world can rapidly become a problem in every part of the world where it impacts can be felt beyond the medical and public health levels.

**Methods:** Therefore, we call for a quantum change in the world’s approach, preparedness and response to pandemics, some posing existential threats to society. Proper preparation should include an international re-evaluation of the role of basic healthcare around the world. The growing threat of future ‘unseen’ enemies requires the adoption of a fundamental new mind set, one with a longer time horizon, new technological tools, including more advanced diagnostics that can be deployed locally, generate high quality data rapidly and can be mass produced.

**Results:** In order to respond better to future threats we should invest and develop pan-viral therapies and more vaccine platforms that can deliver solutions much more rapidly.

**Conclusions:** The Praesens Foundation and its partners are developing new technologies to assist in better surveillance, rapid response to outbreaks and pandemics. This will be illustrated by a new mobile laboratory example.

**Keywords:** Covid-19, pandemics, outbreaks, diagnostics, mobile labs

### Session chair: Jan Y. Verbakel

#### 21. Risk Prediction using Polygenic Scores: What's the State of the Evidence?

##### A. Cecile J.W. Janssens^1^

###### ^1^Department of Epidemiology, Rollins School of Public Health, Emory University, Atlanta GA, USA

####### Contributed session on diagnostic tests

**Background:** Polygenic scores have become the standard for quantifying genetic liability in the prediction of disease risks.

**Methods:** Polygenic scores are generally constructed as weighted sum scores of risk alleles using effect sizes from genome-wide association studies as their weights.

**Results:** The construction of polygenic scores is being improved with more appropriate selection of independent single-nucleotide polymorphisms and optimized estimation of their weights, but other aspects of the research methods are receive little attention. Polygenic prediction research is primarily done in large convenience samples, with no questions asked about the relevance of the data, and, hence, the relevance of the evidence that is being gathered.

**Conclusion:** In this lecture, I will review 15 years of polygenic risk research and discuss lessons learned, lessons not learned, and promising directions for future research.

**Keywords:** Prediction, model, polygenic scores, evidence, genome-wide association studies

## Contributed session on diagnostic tests

### Session chair: Mariska Leeflang

#### 22. A framework to evaluate proposals to change a screening test

##### Sian Taylor-Phillips^1,2^, Lavinia Ferrante di Ruffano^2^, Julia Geppert^1^, Aileen Clarke^1^, Chris Hyde^3^, Russ Harris^4^, Patrick Bossuyt^5^, Jon Deeks^1^

###### ^1^Warwick Medical School, University of Warwick, Coventry, UK; ^2^Institute of Applied Health Research, University of Birmingham, Birmingham, UK; ^3^College of Medicine and Health, University of Exeter, Exeter, UK; ^4^University of North Carolina, North Carolina, USA; ^5^Department of Clinical Epidemiology and Biostatistics, University of Amsterdam, The Netherlands

####### **Correspondence:** Sian Taylor-Phillips

**Background:** Screening programmes are evaluated using randomised controlled trials with lengthy follow-up to morbidity, mortality and overdiagnosis. The fast pace of advances in technology means these trials are often based on outdated screening tests. A framework is needed to guide how to evaluate proposed changes to screening tests.

We aimed to develop a practical framework to evaluate proposed changes to screening tests in established screening programmes, by synthesis of existing methods and development of new theory.

**Methods:** We identified published frameworks for the evaluation of tests and screening programmes (n=64), and existing methods for evaluating or comparing screening tests published in websites of national screening organisations from 16 countries. We extracted principles relevant to evaluation of screening tests. We then searched the same websites for reviews evaluating changes to screening tests (n=484). We analyzed the pathways through which these changes to screening tests affected downstream health, and used these to adapt and extend our framework.

**Results:** We did not find an existing framework specifically designed for evaluation of screening tests across screening programmes. Our proposed framework describes the pathways through which changing a screening test can affect downstream health. Some of these pathways are already included in test evaluation frameworks, e.g. test failures, test accuracy and incidental findings. Some are specific to screening, such as overdiagnosis. We recommend study designs to evaluate these pathways, and recommend a stepwise approach to ensure proportionate review, with the most intensive evaluation required when there is a change to spectrum of disease detected.

**Conclusions:** We present a draft framework for evaluating changes in screening tests. This framework adapts principles from diagnostic test frameworks to the unique challenges of evaluating screening tests, including the complexity in estimating the benefit of earlier detection following screen detection, and associated overdiagnosis.

**Keywords:** Screening, test, systematic review

#### 23. Bias in diagnostic accuracy estimates due to data-driven cutoff selection: a simulation study

##### Brooke Levis^1,2,3^, Parash Mani Bhandari^1,2^, Dipika Neupane^1,2^, Brett D Thombs^1,2^, Andrea Benedetti^2,4^, and the DEPRESsion Screening Data (DEPRESSD) PHQ Collaboration

###### ^1^Lady Davis Institute for Medical Research, Jewish General Hospital, Montréal, Québec, Canada; ^2^Department of Epidemiology, Biostatistics and Occupational Health, McGill University, Montréal, Québec, Canada; ^3^Centre for Prognosis Research, School of Primary, Community and Social Care, Keele University, Staffordshire, UK; ^4^Respiratory Epidemiology and Clinical Research Unit, McGill University Health Centre, Montréal, Québec, Canada

####### **Correspondence:** Brooke Levis

**Background:** Diagnostic accuracy studies with small numbers of cases often use data-driven methods to simultaneously identify an optimal cutoff and estimate its accuracy. When data-driven optimal cutoffs diverge from standard or commonly used cutoffs, authors sometimes argue that sample characteristics influence accuracy and thus different optimal cutoffs are needed for particular population subgroups.

We aimed to explore variability in optimal cutoffs identified and diagnostic accuracy estimates from samples of different sizes and quantify bias in accuracy estimates for data-driven optimal cutoffs, using real participant data on Patient Health Questionnaire-9 (PHQ-9) diagnostic accuracy.

**Methods:** We conducted a simulation study using data from an individual participant data meta-analysis (IPDMA) of PHQ-9 diagnostic accuracy (N studies = 58, N participants = 17,436, N cases = 2,322). 1000 samples of size 100, 200, 500 and 1000 participants were drawn with replacement from the IPDMA database. (Figure 1) Optimal cutoffs (based on Youden’s J) and their accuracy estimates were compared to accuracy estimates for the standard and optimal cutoff of ≥ 10 in the full IPDMA database.

**Results:** Optimal cutoffs ranged from 3-19, 5-14, 5-13, and 6-12 in samples of 100, 200, 500, and 1000 participants, respectively. Compared to estimates for a cutoff of ≥ 10 in the full IPDMA database, sensitivity was overestimated by 10%, 8%, 6% and 5% in samples of 100, 200, 500, and 1000 participants, respectively. Specificity was underestimated by 4% across sample sizes.

**Conclusions:** Using data-driven methods to select optimal cutoffs in small samples leads to large variability in optimal cutoffs identified and exaggerated accuracy estimates, although cutoff variability and sensitivity exaggeration reduce as sample size increases. Researchers should report accuracy estimates for all cutoffs rather than just study-specific optimal cutoffs. Differences in accuracy and optimal cutoffs seen in small studies may be due to the small sample sizes rather than participant characteristics.

**Fig. 1 (abstract 23).**
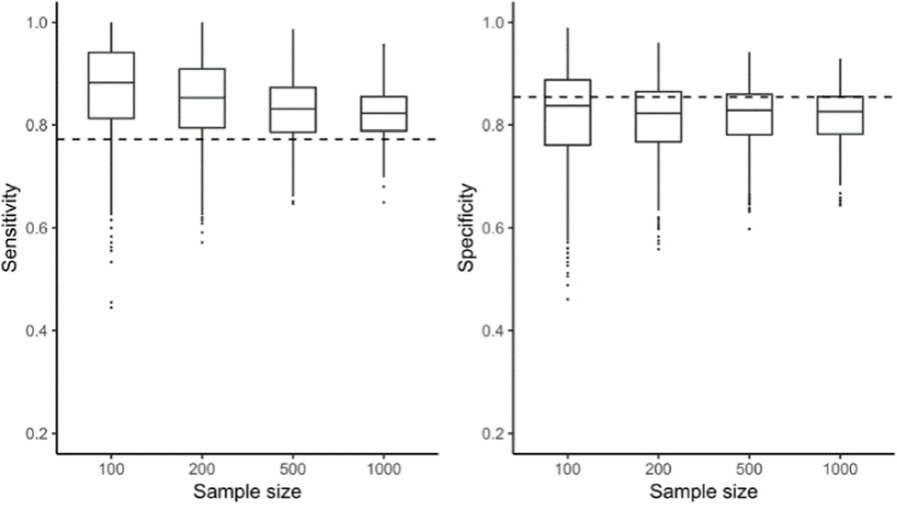
Bias in accuracy results for samples of 100, 200, 500 and 1000 participants

**Keywords:** Diagnostic test accuracy, bias, individual participant data meta-analysis

#### 24. Graphical Enhancements to Summary Receiver Operating Characteristic Plots to Facilitate Diagnostic Test Accuracy Meta-Analysis

##### Amit Patel^1,2^, Nicola J Cooper^2^, Suzanne C Freeman^2^, Alex J Sutton^2^

###### ^1^Cancer Research UK Clinical Trials Unit, Institute of Cancer and Genomic Sciences, College of Medical and Dental Sciences, University of Birmingham, Birmingham, UK; ^2^Biostatistics Research Group, Department of Health Sciences, University of Leicester, Leicester, UK

####### **Correspondence:** Suzanne C Freeman

**Background:** Results of diagnostic test accuracy (DTA) meta-analyses are often presented in two ways: i) forest plots displaying meta-analysis results for sensitivity and specificity separately, and ii) Summary Receiver Operating Characteristic (SROC) curves to provide a global summary of test performance. However other relevant information on included studies is often not presented graphically and in the context of the results.

We aimed to develop graphical enhancements to SROC plots to address shortcomings in the current guidance on graphical presentation of DTA meta-analysis results.

**Methods:** A critical review of guidelines for conducting DTA systematic reviews and meta-analyses was conducted to establish and critique current recommendations for best practice for producing plots. New plots addressing shortcomings identified in the review were devised and implemented in MetaDTA [1].

**Results:** Two primary shortcomings were identified: i) lack of incorporation of quality assessment results into the main analysis and; ii) ambiguity with how the contribution of individual studies to the meta-analysis are represented on SROC curves. In response, two novel graphical displays were developed: i) *A quality assessment enhanced SROC plot* which displays the results from individual studies in the meta-analysis using glyphs to simultaneously represent the multiple dimensions of quality assessed using QUADAS-2; and ii) *A percentage study weights enhanced SROC plot* which accurately portrays the percentage contribution each study makes to both sensitivity and specificity simultaneously using ellipses.

**Conclusions**: The proposed enhanced SROC curves facilitate the exploration of DTA data, leading to a deeper understanding of the primary studies including identifying reasons for between-study heterogeneity and why specific study results may be divergent. Both plots can easily be produced in the free online interactive application, MetaDTA [1].

**Keywords**: Diagnostic test accuracy, meta-analysis, visualisation

**References**

[1] Freeman et al. Development of an interactive web-based tool to conduct and interrogate meta-analysis of diagnostic test accuracy studies: MetaDTA. *BMC Med Res Methodol,* 2019;19:81

#### 25. Network meta-analysis of cerebrospinal fluid and blood biomarkers for the diagnosis of sporadic Creutzfeldt-Jakob disease

##### Nicole Rübsamen^1^, Stephanie Pape^1^, André Karch^1^

###### ^1^Institute for Epidemiology and Social Medicine, University of Münster, Domagkstraße 3, 48149 Münster, Germany

####### **Correspondence:** Nicole Rübsamen

**Background:** Several biomarkers have been proposed for the diagnosis of sporadic Creutzfeldt-Jakob disease (sCJD), the most prevalent form of human prion disease.

We identified and evaluated all relevant diagnostic studies for the biomarker-based differential diagnosis (using serum or cerebrospinal fluid biomarkers) of sCJD, and combined direct and indirect evidence from these studies in a network meta-analysis.

**Methods:** We systematically searched Medline (via PubMed), Embase, and the Cochrane Library. To be eligible, studies had to include the established diagnostic criteria of sCJD and established diagnostic criteria for other forms of dementia as reference standard. The studies had to provide sufficient information to construct the 2×2 contingency table (i.e., false and true positives and negatives). We registered the study protocol with PROSPERO, number CRD42019118830. Risk of bias was assessed with the QUADAS-2 tool. We used a bivariate model to conduct meta-analyses of individual biomarkers and to estimate the between-study variability in logit sensitivity and specificity. To investigate sources of heterogeneity, we performed subgroup analyses based on QUADAS-2 quality and clinical criteria. We used a Bayesian beta-binomial analysis of variance model for the network meta-analysis.

**Results:** We included eleven studies, which investigated 14-3-3 (n=11), NSE (n=1), RT-QuIC (n=3), S100B (n=3), and tau (n=9). Heterogeneity was high in the meta-analyses of individual biomarkers and different depending on the level of certainty of sCJD diagnosis. In the network meta-analysis, 14-3-3 was the most sensitive, but among the least specific test, while RT-QuIC was the most specific though among the least sensitive test.

**Conclusions:** Our work shows the weaknesses of previous diagnostic accuracy studies. Subgroup analyses will reveal if our results depend on methodological quality of the studies or clinical criteria of the patients.

**Keywords:** Blood, cerebrospinal fluid, neurodegeneration, diagnosis, sporadic Creutzfeldt-Jakob disease

#### 26. The potential for seamless designs in traditional diagnostic research?

##### Werner Vach^1^, Eric Bibiza-Freiwald^2^ , Oke Gerke^3^, Tim Friede^4^, Patrick M Bossuyt^5^, Antonia Zapf^2^

###### ^1^Department of Clinical Research, University of Basel, Schanzenstrasse 55, CH-4031 Basel, Switzerland; ^2^Institute of Medical Biometry and Epidemiology, University Medical Center Hamburg-Eppendorf, Martinistraße 52, D-20246 Hamburg, Germany; ^3^Department of Nuclear Medicine, Odense University Hospital & Department of Clinical Research, University of Southern Denmark, J. B. Kløvervænget 47, DK-5000 Odense C, Denmark; ^4^Department of Medical Statistics, University Medical Center Goettingen, Humboldtallee 32, D-37073 Goettingen, Germany; ^5^Department of Clinical Epidemiology, Biostatistics and Bioinformatics, Amsterdam University Medical Centers, Postbus 22660, NL-1100 DD Amsterdam, The Netherlands

####### **Correspondence:** Werner Vach

**Background:** New diagnostic tests to identify a well-established disease state have to undergo a series of scientific studies from test construction until finally demonstrating a societal impact. Traditionally, these studies are performed with substantial time gaps in between. Seamless designs allow us to combine a sequence of studies in one protocol and may hence accelerate this process.

We performed a systematic investigation of the potential of seamless designs in diagnostic research.

**Methods:** We summarized the major study types in diagnostic research and identified their basic characteristics with respect to applying seamless designs. This information was used to identify major hurdles and opportunities for seamless designs.

**Results:** 11 major study types were identified. The following basic characteristics were identified: type of recruitment (case-control vs population-based), application of a reference standard, inclusion of a comparator, paired or unpaired application of a comparator, assessment of patient relevant outcomes, possibility for blinding of test results. Two basic hurdles could be identified: 1) Accuracy studies are hard to combine with post-accuracy studies, as the first are required to justify the latter and as application of a reference test in outcome studies is a threat to the study’s integrity. 2) Questions, which can be clarified by other study designs, should be clarified before performing a randomized diagnostic study. However, there is a substantial potential for seamless designs since all steps from the construction until the comparison with the current standard can be combined in one protocol. This may include a switch from case-control to population-based recruitment as well as a switch from a single arm study to a comparative accuracy study. In addition, change in management studies can be combined with an outcome study in discordant pairs. Examples from the literature illustrate the feasibility of both approaches.

**Conclusions:** There is a potential for seamless designs in diagnostic research.

**Keywords**. Test construction studies, accuracy studies, randomized diagnostic studies, seamless design, blinding

#### 27. Meta-analysis of diagnostic test accuracy using multivariate probit models

##### Enzo Cerullo^1^, Hayley Jones^2^, Terry Quinn^3^, Nicola Cooper^1^, Alex Sutton^1^

###### ^1^NIHR Complex Reviews Support Unit, Department of Health Sciences, University of Leicester, Leicester, UK; ^2^Population Health Sciences, Bristol Medical School, University of Bristol, Bristol, UK; ^3^NIHR Complex Reviews Support Unit, Institute of Cardiovascular and Medical Sciences, University of Glasgow, Glasgow, UK

####### **Correspondence:** Enzo Cerullo

**Background:** Multivariate probit models are used to analyze correlated ordinal data. In the context of diagnostic test accuracy without a gold standard test, their use has been more limited. Multivariate probit models have been used for the analysis of dichotomous and categorical (>1 threshold) diagnostic tests in a single study, and for the meta-analysis of dichotomous tests.

We aimed to (i) develop a model for the meta-analysis of multiple binary and categorical diagnostic tests without a gold standard; (ii) extend the model to enable estimation of joint test accuracy.

**Methods:** We extended proposed multivariate probit models for the meta-analysis of diagnostic test accuracy, modelling the conditional within-study correlations between tests. Dichotomous tests use binary multivariate probit likelihoods and categorical tests use ordered likelihoods. We also showed how the model can be extended to estimate joint test accuracy, to meta-analyse studies which report accuracy at distinct thresholds, and how to incorporate priors for the 'gold standard' tests based on inter-rater agreement information. We fitted the models using Stan which uses a state-of-the-art Hamiltonian Monte Carlo algorithm.

**Results:** We applied the methods to a dataset in which studies evaluated the accuracy of tests for deep vein thrombosis, where studies included two dichotomous tests and one categorical test. We compared our results to the original study, which assumed a perfect reference test. In Stan, we found estimation to be very slow for meta-analyses which contained large studies with sparse data. We discuss these computational issues and possible ways to improve scalability by making use of recently proposed algorithms, such as calibrated data augmentation Gibbs sampling.

**Conclusions:** We developed a model for the meta-analysis of multiple, categorical diagnostic tests without a gold standard. Unlike latent class models, they can be extended to tackle a variety of problems without having to inappropriately simplify or discard data.

**Keywords:** Meta-Analysis, diagnostic, test, accuracy, probit, imperfect, gold, reference, thresholds, interrater, agreement

## Contributed session on prediction models

### Session chair: Maarten Van Smeden

#### 28. Performance of Heart Failure Clinical Prediction Models: A Systematic External Validation Study

##### Jenica N. Upshaw^1,2^, Jason Nelson^1^, Benjamin Koethe^1^, Jinny G. Park^1^, Hannah McGinnes^1^, Benjamin S. Wessler^1,2^, Ben Van Calster PhD^3^, David van Klaveren PhD^1,4^, Ewout Steyerberg PhD^4^, David M. Kent MD MS^1^

###### ^1^Predictive Analytics and Comparative Effectiveness (PACE) Center, Institute for Clinical Research and Health Policy Studies (ICRHPS), Tufts Medical Center, 800 Washington St, Boston, MA, USA; ^2^Division of Cardiology, Tufts Medical Center, 800 Washington St, Boston, MA, USA; ^3^Department of Development and Regeneration, KU Leuven, Leuven, Belgium; ^4^Department of Biomedical Data Sciences, Leiden University Medical Center, Leiden, The Netherlands

####### **Correspondence:** Jenica N. Upshaw

**Background**: Most heart failure (HF) clinical prediction models (CPMs) have not been independently externally validated.

We aimed to test the performance of HF CPMs using a systematic approach.

**Methods**: We performed a systematic review to identify CPMs predicting outcomes in HF, stratified by acute and chronic HF CPMs. External validations were performed using individual patient data from 8 large HF trials. CPM discrimination (c-statistic, % relative change in c-statistic) as well as calibration (Harrell's E, E90, net benefit) was estimated for each CPM with and without recalibration.

**Results**: Of 135 HF CPMs screened, 24 (18%) were matched on population, predictors and outcomes to the trials and 42 external validations were performed. The median derivation c-statistic of acute HF CPMs was 0.76 (IQR, 0.75-0.8), validation c-statistic was 0.67 (0.65, 0.68) and model-based c-statistic was 0.68 (0.66, 0.76), demonstrating that most of the decrement in model performance was due to narrower case-mix in the validation cohort compared with the development cohort. The median derivation c-statistic for chronic HF CPMs was 0.76 (0.74, 0.8), validation c-statistic 0.61 (0.6, 0.63) and model-based c-statistic 0.68 (0.62, 0.71), thus decrement in model performance was only partially due to case-mix heterogeneity. The median E (standardized by outcome rate) was 0.5 (0.3, 2.2) for acute HF CPMs and 0.6 (0.3, 0.7) for chronic HF CPMs. Updating the intercept alone led to a significant improvement in calibration in acute HF CPMs, but not chronic HF CPMs. Net benefit analysis showed potential for harm in using CPMs when decision threshold was not near the overall outcome rate but this improved with model recalibration (Table).

**Conclusions:** A small minority of published CPMs were matched to clinical trial datasets. For acute HF CPMs, discrimination is largely preserved after adjusting for case-mix; however, model updating is required for both acute and chronic HF CPMs.

**Table 1 (abstract 29).**
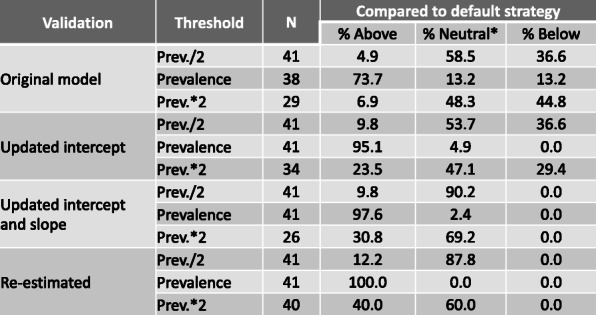
Effects of updating on net benefit by decision curve analysis. Threshold refers to decision threshold and Prev./2 refers to the net benefit when the decision threshold is half the event rate, prevalence means the decision threshold is at the outcome prevalence and Prev.*2 refers to decision threshold at twice the outcome prevalence.

**Keywords:** Clinical prediction model, heart failure, mortality

#### 29. Informative Presence and Observation: A review of methods for clinical risk prediction

##### Rose Sisk^1^, Lijing Lin^1^, Glen P. Martin^1^, Niels Peek^1^, Matthew Sperrin^1^

###### ^1^Health e-Research Centre, Division of Informatics, Imaging and Data Science, University of Manchester, UK

####### **Correspondence:** Rose Sisk

**Background:** Clinical prediction models (CPMs) are increasingly developed and validated using electronic health records (EHRs) since they provide rich, longitudinal information on a patient’s interactions with healthcare services. The analysis of such data is not, however, without challenges. Specifically, the observation process of EHRs is dependent on the underlying health status of the individual, which not only leads to irregularly collected information, but importantly means that the type, timing, and frequency of data collection could be informative with respect to a patient’s health status. This is referred to as “informative presence” and “informative observation”. Informative presence/observation may be an opportunity, as the additional information contained within the observation process could improve accuracy of prediction models.

This project aims to synthesise the existing analytical methodology that could be used to allow CPMs to learn from “informative presence” and “informative observation”. In doing so, we aim to identify remaining methodological challenges in this area.

**Methods:** A systematic literature search was conducted by two independent reviewers. Keywords were identified and used to search Embase, MEDLINE and Web of Science. Articles were screened based on title and abstract at stage one, and full texts at stage two for any remaining papers.

**Results:** All methods (within 37 papers) discovered during this review broadly fall under three categories; methods which use derived information about the observation process as model predictors (e.g. counts of observations or visits), methods which make indirect use of the observation process via a latent structure (e.g. through random effects in joint models), or methods that model under informed missingness.

**Conclusions:** Methodology to incorporate informative presence/observation in CPMs is beginning to emerge, and shows promise in improving the performance of prediction models. However this is still an underdeveloped area, and further work should explore where each method improves predictive accuracy.

**Keywords**: Informative, observation, presence, absence, prediction, model

#### 30. Penalisation and shrinkage methods do not guarantee a reliable prediction model

##### Gary S Collins^1^, Richard D Riley^2^

###### ^1^Centre for Statistics in Medicine, Nuffield Department of Orthopaedics, Rheumatology and Musculoskeletal Sciences, University of Oxford, Oxford, UK; ^2^Centre for Prognosis Research, School of Primary, Community and Social Care. Keele University, Staffordshire, UK

####### **Correspondence:** Gary S Collins; Richard D Riley

**Background:** Prediction models are often developed using a multivariable regression framework (e.g. logistic, survival, or linear regression), which provides an equation to estimate an individual's outcome probability (for binary or time-to-event outcomes) or outcome value (for continuous outcomes) conditional on values of multiple variables (‘predictors’). When estimating such equations using a particular dataset, standard estimation techniques are often used, in particular ordinary least squares or maximum likelihood estimation. However, when applied in new individuals, these fitted equations tend to produce optimistic (i.e. too extreme) predictions; that is, the predicted outcome probability (from logistic or time-to-event regression models) or the predicted outcome value (from linear regression) for new individuals is too far from the mean for some individuals. This is a particular concern when the number of predictors is large relative to the sample size, such that overfitting is a concern.

**Methods:** To address the issue of overfitting, penalisation estimation techniques are increasingly being recommended, especially for situations where the effective sample size is low. These include uniform shrinkage (e.g. estimated via bootstrapping), the lasso, elastic net, and ridge regression. Many researchers believe such methods resolve the issue of overfitting entirely.

**Results:** In this talk we highlight that penalisation methods are no substitute for obtaining large sample sizes for model development. In particular, through examples, simulation and analytic reasoning, we show that shrinkage and penalty factors are typically estimated with large uncertainty, especially in small development datasets where the potential for overfitting is large.

**Conclusion:** We discuss and illustrate approaches to reduce this uncertainty for the lasso, elastic net and ridge regression, and reinforce guidance for how to derive the sample size needed to develop a model.

**Keywords**: Prediction, shrinkage, penalisation, uncertainty

#### 31. Evaluating the prognostic performance of a polygenic risk score model for breast cancer risk stratification

##### Maria Olsen^1^, Krista Fischer^2,3^, Patrick M. Bossuyt^1^, Els Goetghebeur^4^

###### ^1^Amsterdam University Medical Centers, dept. of Clinical Epidemiology, Biostatistics and Bioinformatics, Amsterdam Public Health Research Institute, Meibergdreef 9, 1105 AZ Amsterdam, The Netherlands; ^2^Institute of Mathematics and Statistics, University of Tartu, Narva mnt 18, 51009 Tartu, Estonia; ^3^Estonian Genome Center, Institute of Genomics, University of Tartu, Estonia; ^4^Ghent University, dept. of Applied Mathematics, Computer Science and Statistics, Institute for Continuing Education Center for Statistics, Campus Sterre, S9, Krijgslaan 281, 9000 Gent, Belgium

####### **Correspondence:** Maria Olsen

**Background:** Polygenic risk scores (PRS) can be used in breast cancer (BC) risk stratification to improve current screening programs. Recently, Läll et al. developed a PRS based on the Estonian Biobank (EstBB) cohort ^[1]^. We analyzed how well this PRS performs in estimating women’s future risk of developing BC.

We aimed to estimate the cumulative BC incidence for women in the EstBB cohort, using the prevalence-based PRS^[1]^ as predictor together with year of cohort-entry, age, BMI, smoking status, educational level and prevalent co-morbidities. To evaluate the prognostic performance of PRS-based risk.

**Methods:** We included data on 30,312 women from the EstBB cohort, between 20-89 years and without a history of BC. We estimated absolute 3 and 5-year PRS-based risk with a Cox Proportional Hazards model, retaining PRS and age as covariates. Performance of the age-adjusted PRS-based risk was assessed in terms of cross-validated calibration, discrimination, and reclassification.

**Results:** Calculated risks derived from the age-adjusted PRS-model were consistent with the observed cross-validated 3-year cumulative incidence of 0.33% and 5-year cumulative incidence of 0.61% for the entire cohort. The AUC was 0.720 (95% CI: 0.675 to 0.765) for 3 years and 0.704 (95% CI: 0.670 to 0.737) for 5 years. Compared to an age-only model, this was just 0.022 higher for 3 years and 0.023 higher for 5 years. Reclassification analysis, using a 1% risk cut-off, showed that few but overall more women were correctly vs incorrectly reclassified (3-year NRI 0.094; 5-year NRI 0.0527).

**Conclusion:** Despite good calibration, we found modest incremental performance improvement of the PSR-based risk compared to age-based. A considerably larger study would be needed to assess whether the PRS could meaningfully contribute to the development of more efficient screening strategies.

**Keywords:** Prognostic accuracy, Breast cancer, Polygenic risk score, Precision screening, Risk stratification, Medical test evaluation, Biomarker evaluation, Performance measures.

**References:**

^[1]^ Läll K, Lepamets M, Palover M, Esko T, Metspalu A, Tõnisson N, Padrik P, Mägi R, Fischer K. Polygenic prediction of breast cancer: comparison of genetic predictors and implications for risk stratification. BMC Cancer. 2019 Jun 10;19(1):557.

#### 32. Systematic Review of Prognostic Models for Recurrent Event Data

##### Victoria Watson^1^, Catrin Tudur Smith^1^, Laura Bonnett^1^

###### ^1^University of Liverpool, Department of Biostatistics, Block F Waterhouse Building, 1-5 Brownlow Street, Liverpool, L69 3GL, UK

####### **Correspondence:** Victoria Watson

**Background:** Prognostic models predict outcome for people with an underlying medical condition. Many conditions are typified by recurrent events such as seizures in epilepsy. Prognostic models for recurrent events can be utilised to predict individual patient risk of disease recurrence or outcome at certain time points.

**Methods:** Methods for analysing recurrent event data are not widely known or applied in research. Most analyses use survival analysis to consider time until the first event, meaning subsequent events are not analysed and key information is lost. An alternative is to analyse the event count using Poisson or Negative Binomial regression. However, this ignores the timing of events.

**Results:** Therefore, a systematic review on methodology for analysing recurrent event data in prognostic models is ongoing. Results from this review will identify methods commonly used in practice. Information such as the event rate of the underlying condition will be collected to determine whether model choice might be influenced by this factor.

**Conclusions:** Results from this review will be presented including a summary of each method identified. The results will be the first step towards a toolkit for future analysis of recurrent event data.

**Keywords:** Prognostic models, systematic review, recurrent event data

#### 33. Prognostic model to clinical tool: the OxMIV tool for violence risk in psychiatry

##### Daniel Whiting^1^

###### ^1^University of Oxford, Department of Psychiatry, Warneford Hospital, Oxford, UK

**Background:** A gulf exists between the number of prognostic models developed and those effectively adopted as clinical tools. Bridging this gap requires recognition that a tool will not sit in isolation, but be integrated into an often complex clinical system. Utility therefore rests on both providing accurate predictions in a target population, and on understanding a tool's role and acceptability in a clinical system.

The OxMIV prediction model for violence in severe mental illness has been internally and externally validated using Swedish population registers.^[1]^ The current project aims to externally validate and develop OxMIV as a clinical tool for UK community mental health teams who engage individuals with first-episode psychosis. This study will examine how OxMIV can be used in a clinical setting- and specifically its clinical acceptability, to develop a framework for its implementation and wider evaluation.

**Methods:** Mixed methods are used to examine the process of integrating OxMIV into community mental health teams in two counties. Interviews with 20 multidisciplinary clinicians focus on acceptability and barriers to use. Approaches to examining uptake, reach and utility are piloted using structured data from electronic records.

**Results:** Pilot work demonstrated the feasibility of using routine data for validation, and showed 1 in 10 individuals under the care of these services were arrested for violence in 12-months, but no structured framework currently exists to determine risk. Preliminary findings from work to develop OxMIV for this role will be discussed, focusing on transferrable themes pertinent to the clinical translation of prediction models.

**Conclusions:** These will include service management perspectives, interface with electronic systems, risk communication, decision pathways, and clinician views on the desirable properties of a usable tool.

**Keywords:** Prediction, model, clinical, psychiatry, psychosis, violence

**References:**

^[1]^ S. Fazel, A. Wolf, H. Larsson, P. Lichtenstein, S. Mallett, T. Fanshawe, *Lancet Psychiatry,* 4(6) 2017, 461-468.

#### 34. Methods for misclassified exposures in individual participant data meta-analysis

##### Valentijn M.T. de Jong^1^, Harlan Campbell^2^, Thomas Jaenisch^3^, Paul Gustafson^2^, Thomas P.A. Debray^1,4^

###### ^1^Julius Center for Health Sciences and Primary Care, University Medical Center Utrecht, Utrecht University, Utrecht, The Netherlands; ^2^Department of Statistics, University of British Columbia, Vancouver, Canada; ^3^Department for Infectious Diseases, Heidelberg University Hospital, Heidelberg, Germany; ^4^Cochrane Netherlands, University Medical Center Utrecht, Utrecht, The Netherlands

####### **Correspondence:** Valentijn M.T. de Jong

**Background:** Measurement error of binary variables (misclassification) is a common problem in the analysis of multiple data sources, including individual participant data meta-analysis (IPD-MA). Misclassification may lead to biased parameter estimates, even when the misclassification is entirely random. Available methods for addressing misclassification do not account for between-study heterogeneity in an IPD-MA.

We aimed to develop statistical methods that facilitate unbiased estimation of logistic regression models for a one-stage IPD-MA, where the extent and nature of misclassification may vary across studies. We focus on the estimation of predictor-outcome associations and between-study heterogeneity.

**Methods:** We present Bayesian methods that allow misclassification to be dependent on study-level and participant-level characteristics. We illustrate this in an example of the differential diagnosis of dengue using two predictors, where the gold standard measurement for one (muscle pain) is unavailable for some studies, which only measured a surrogate prone to misclassification. We present a simulation study to assess bias, root mean square error (RMSE), coverage and power in estimating the muscle pain-dengue association.

**Results:** In the example, our methods yielded estimates with less error than analyses naive with regard to misclassification or based on gold standard measurements alone. Minor differences were observed in the estimates of heterogeneity of the muscle pain-dengue association.

In our simulations, adjusting the one-stage IPD-MA models for misclassification lead to valid estimates of the adjusted predictor-outcome association, with less RMSE, greater power and similar coverage compared to an analysis restricted to available gold standard measurements.

**Conclusion:** Our proposed framework can account for the presence of predictor misclassification in IPD-MA. It requires that 1) some studies supply IPD for the surrogate and gold standard variables and 2) misclassification is exchangeable across studies conditional on observed covariates (and outcome). Further work is needed for other types of misclassification.

**Keywords:** Meta-analysis, misclassification, measurement error, individual-participant-data (IPD)

## Invited talk: Xiaoxuan Liu

### Session chair: Ben Van Calster

#### 35. Improving the Quality of Evidence for Artificial Intelligence in Healthcare

##### Xiaoxuan Liu^1,2,3,4,5^

###### ^1^Academic Unit of Ophthalmology, Institute of Inflammation and Ageing, College of Medical and Dental Sciences, University of Birmingham, UK; ^2^Department of Ophthalmology, University Hospitals Birmingham NHS Foundation Trust, Birmingham, UK; ^3^Moorfields Eye Hospital NHS Foundation Trust, London, UK; ^4^Health Data Research UK, London, UK; ^5^Birmingham Health Partners Centre for Regulatory Science and Innovation, University of Birmingham, Birmingham, UK

**Background:** Advances in artificial intelligence (AI)/machine learning (ML) have attracted significant attention in recent years for their potential applications in healthcare. A vast body of literature has been published proposing AI/ML-based solutions for disease detection, classification, prediction, or even as therapeutic interventions. However, there are concerns that the quality of evidence has not been sufficiently robust to support safe and effective deployment of AI algorithms in clinical care. In this talk, some of the major limitations of reporting in clinical AI studies will be presented.

**Methods:** To address this urgent evidence gap, several new reporting guidelines have been developed or are currently in development. The first of these, published in September 2020, is the SPIRIT-AI (Standard Protocol Items: Recommendations for Interventional Trials –AI) and CONSORT-AI (Consolidated Standards of Reporting Trials–AI) extensions for clinical trials evaluating AI interventions.

**Results:** SPIRIT-AI and CONSORT-AI include recommendations which are AI-specific, such as asking authors to provide clear descriptions of the AI system, including instructions and skills required for use, the operational environment in which the AI intervention is integrated, the handling of input and output data of the AI system, the human–AI interaction and provision of an analysis of error cases.

**Conclusion:** SPIRIT-AI and CONSORT-AI will help promote transparency and completeness of studies in this area. It will assist editors and peer reviewers, as well as the general readership, to understand, interpret and critically appraise the quality of clinical trial design and risk of bias in the reported outcomes.

**Keywords**: Artificial intelligence, machine Learning, clinical trials, randomised controlled trials, protocol, reporting guidelines

## Poster presentations

### List of abstracts (in alphabetic order by presenting author)

#### 36. Predicting pre-eclampsia in nulliparous women using routinely-collected maternal characteristics: A model development and validation study

##### Ziad TA Al-Rubaie^1^, H Malcolm Hudson^2^, Gregory Jenkins^3^, Imad Mahmoud^4^, Joel G Ray^5^, Lisa M Askie^2^, Sarah J Lord^1,2^

###### ^1^School of Medicine, The University of Notre Dame Australia, 160 Oxford Street, Darlinghurst NSW 2010, Australia; ^2^NHMRC Clinical Trial Centre, University of Sydney, Level 6 Medical Foundation Building, 92 Parramatta Road, Camperdown NSW 2050, Australia; ^3^Department of Obstetrics, Westmead Hospital, Suite 110, 9 Norbrik Drive, Bella Vista, NSW 2153, Australia; ^4^Department of Obstetrics, Auburn and Mount-Druitt and Blacktown Hospitals, Suite 108, 9 Norbrik Drive, Bella Vista, NSW 2153, Australia; ^5^Department of Medicine, St. Michael’s Hospital, 30 Bond Street, Toronto, Ontario, M5B 1W8, Canada

####### **Correspondence:** Ziad TA Al-Rubaie

**Background:** Guidelines recommend identifying in early pregnancy women at elevated risk of pre-eclampsia. Existing prediction tools perform poorly among nulliparous women.

We aimed to 1) develop and validate a pre-eclampsia risk prediction model for nulliparous women. 2) compare the model’s performance against the existing NICE approach.

**Methods:** This retrospective cohort study included all nulliparous women who gave birth in three public hospitals, Western-Sydney-Local-Health-District, Australia, 2011-2014. Using births from 2011-2012, we performed multivariable logistic regression incorporating established maternal risk factors to develop, and internally validate, the “Western Sydney (WS) model”. The WS model was externally validated using births from 2013-2014, assessing its discrimination and calibration. We fitted the final WS model for all births from 2011-2014, and compared its accuracy with the NICE approach.

**Results:** Among 12,395 births, 293 women (2.4%) had pre-eclampsia. The WS model included: maternal age, BMI, ethnicity, multiple pregnancy, family history of pre-eclampsia, autoimmune disease, chronic hypertension and chronic renal disease. In the validation sample (N=6201), the model c-statistic was 0.70 [95% CI 0.65–0.75], suggesting good discrimination. The observed:expected ratio for pre-eclampsia was 0.91, and the Hosmer-Lemeshow *p*-value of 0.20 suggesting good calibration. In the entire sample (N=12,395), 374 (3.0%) women had a WS model-estimated pre-eclampsia risk ≥8%, the risk-threshold for considering aspirin prophylaxis. Of these, 54 (14.4%) developed pre-eclampsia (sensitivity 18% [14–23], specificity 97% [97–98]). Using the NICE approach, 1173 (9.5%) women were classified as high-risk, of which 107 (9.1%) developed pre-eclampsia (sensitivity 37% [31-42], specificity 91% [91–92]). The final model showed similar accuracy to NICE approach when using a lower risk-threshold ≥4%.

**Conclusions:** This WS risk model achieved modest performance for pre-eclampsia prediction in nulliparous women. Although not superior to the NICE approach, the WS model has the advantage of providing individualised risk-estimates to inform decisions for pregnancy surveillance and aspirin prophylaxis.

**Keywords:** Antenatal care, Australia, maternal health, National Institute of Health and Care Excellence, pre-eclampsia, prediction, risk assessment, risk prediction model

#### 37. Why are Machine Learning-based prediction models still unpopular in clinical practice?

##### Constanza L Andaur Navarro^1,2^, Johanna Damen^1,2^, Toshihiko Takada^1,2^, Paula Dhiman^3^, Gary S Collins^3^, Jie Ma^3^, Ram Bajpai^4^, Richard D. Riley^4^, Lotty Hooft^1,2^, Karel G Moons^1,2^

###### ^1^Julius Center for Health Sciences and Primary Care, UMC Utrecht, Utrecht University, Universiteitsweg 100, 3584 CG Utrecht, The Netherlands; ^2^Cochrane Netherlands, UMC Utrecht, Utrecht University, Universiteitsweg 100, 3584 CG Utrecht, The Netherlands; ^3^Center for Statistics in Medicine, University of Oxford, Windmill Road, OX1 2JD Oxford, UK; ^4^School of Primary, Community and Social Care, Keele University, David Weatherall Building, ST5 5BG Keele, UK

####### **Correspondence:** Constanza L Andaur Navarro

**Background:** Studies addressing diagnostic and prognostic prediction models are abundant in many clinical domains. At the same time, many systematic reviews showed that the quality of reporting of prediction model studies is suboptimal.^[1]^ Due to the increasing availability of larger, routinely collected and complex data, and the rising application of Artificial Intelligence (AI) and Machine Learning techniques (ML) for clinical predictions, the number of prediction models is expected to increase even further. These AI/ML-based prediction model studies are often labeled as a "black box" and not much is known yet about the quality of reporting.

The aim of this systematic review is to evaluate the reporting and methodological conduct of prediction model studies that applied AI/ML techniques for model development or validation.

**Methods:** Our protocol was registered in PROSPERO (CRD42019161764). A search was performed in January 2020 to identify primary studies developing and/or validating prediction models using any AI/ML methodology across all medical fields. Studies were included if predicted patient-related outcomes, used any study design and were published in 2018-2019. We assessed (1) the quality of reporting by measuring the adherence to Transparent Reporting of a multivariable prediction model for Individual Prognosis or Diagnosis guideline (TRIPOD) and (2) the risk of bias in prediction model development or validation using the Prediction model Risk of Bias Assessment Tool (PROBAST).

**Results:** Initial results from the review will be presented, stratified by medical field and prevalent AI/ML methods.

**Conclusions:** Emerging issues will be discussed, as well as the necessity for specific reporting (TRIPOD-AI/ML) and risk of bias (PROBAST AI/ML) assessment for AI/ML-based prediction model studies.

**Keywords:** Systematic review, machine learning, prediction

**References**

^[1]^ Heus P, Damen JAAG, Pajouheshnia R, et al. Poor reporting of multivariable prediction model studies: Towards a targeted implementation strategy of the TRIPOD statement. BMC Med. 2018;16(1):1-12.

#### 38. Development of prediction models using competing risk models in big healthcare databases

##### Constantinos Koshiaris^1^, Richard Stevens^1^, Richard Riley^2^, Sarah Lay-Flurrie^1^, Kym Snell^2^, Lucinda Archer^2^, James Sheppard^1^

###### ^1^Nuffield Department of Primary Care Health Sciences, University of Oxford, Radcliffe Primary Care Building, Radcliffe Observatory Quarter, Woodstock Rd, Oxford, UK; ^2^Centre for Prognosis Research, School of Primary, Community and Social Care, Keele University, Staffordshire, UK

####### **Correspondence:** Lucinda Archer

**Background:** The Cox proportional hazards model is a commonly used method when developing prognostic prediction models using time-to-event data. In the presence of competing risks - events that might prevent the occurrence of the event of interest – using a Cox model leads to predicted probabilities that are too high. Thus methods that account for competing risks, such as the Fine-Gray model, are preferred. However, fitting this model is computationally complex, particularly when used in combination with multiple imputation and fractional polynomials. This poses a significant challenge when developing prediction models in big databases.

We aimed to describe prediction modelling approaches that minimise computation time, without compromising model validity, in a large dataset of electronic health records.

**Data:** Data from the Clinical Practice Research Datalink were used to create prognostic models for adverse events related to antihypertensive medication, treating death as a competing event (prevalence 10%). The dataset included 1,773,224 patients and 40 predictors.

**Methods/Results:** A multivariable competing risk model developed using the stcrreg command in STATA 16 (8 cores) required approximately two weeks to converge using an 8 core 32GB, i9 PC. Computation time was reduced to less than one day when estimating regression coefficients using the R package fastcmprsk, which uses a forward backward scan algorithm: this is more efficient than the Newton-Raphson method. Robust bootstrap confidence intervals were estimated using the percentile method. Fractional polynomial transformations were computationally prohibitive, thus variable transformations were modelled with the use of Cox regression, providing a good approximation of the relationship.

**Conclusions:** Computational obstacles to correctly account for competing risks in clinical prediction models can be overcome by combining fast algorithms, robust bootstraps and approximate fractional polynomials transformations. We are currently investigating how to optimise the use of the Fine-Gray model in conjunction with multiple imputation.

**Keywords:** Prediction, competing risks, big data

#### 39. Weighted variogram analyses for estimating within-patient variance components using routine data from biomarker monitoring programmes

##### S. Baldwin^1,2^, A. Sitch^1,2^, Y. Takwoingi^1,2^, J. Deeks^1,2^

###### ^1^Test Evaluation Research Group, Institute of Applied Health Research, University of Birmingham, Edgbaston, Birmingham, B15 2TT, UK; ^2^National Institute for Health Research Birmingham Biomedical Research Centre, University Hospitals Birmingham NHS Foundation Trust and University of Birmingham, UK

####### **Correspondence:** S. Baldwin

**Background:** Measurement error in biomarkers is best estimated in Biological Variability Studies (BVS) where individuals have repeated measures both at the same and at different time points. However, BVS are not always feasible. We investigate whether measurement error can be estimated using routine data from biomarker monitoring programmes by application of a method known as the variogram.

We aimed to demonstrate the potential of the variogram using open-source monitoring programme data of serum albumin measurements on stage 2-4 primary biliary cirrhosis patients.

**Methods:** Variation in measurements from patients over time includes three components: true differences at baseline between-patients; true changes from baseline within-patients (‘signal’); and measurement error (‘noise’). The variogram considers differences within-patients computed between baseline and each follow-up point; the variances of these differences increase at a rate dependent on the magnitude of the within-patient variability. We grouped measurements by year, and assigned weights according to the closeness of the actual time to the midpoint using a Gaussian kernel approach. Weighted variances of differences in serum albumin were calculated per time. The variogram is a plot of weighted variance of differences (y-axis) against time (x-axis), with a fitted line estimated by linear regression, weighted according to sample size. Extrapolation of the fitted line to intersect the y-axis was used to estimate the measurement error (‘noise’).

**Results:** The measurement error (‘noise’) estimate was 0.10 (gm/dL)^2^. (Figure 1) ‘Signal’ first surpassed ‘noise’ at five years; the variance of differences at one year was estimated almost entirely ‘noise’. Such results from weighted variogram analyses could be used to help define optimal measurement timings for monitoring programmes.

**Fig. 1 (abstract 39).**
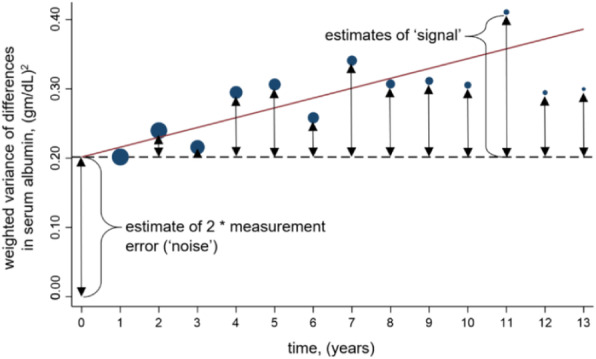
weighted variogram analysis

**Conclusions:** Weighted variogram analyses have potential for application where health status changes are unlikely; care should be exercised in implementation, particularly related to bias from dropout.

**Keywords:** Variogram, variability, monitoring, measurement error

#### 40. Bayesian Hierarchical Models for Personalized Health Care

##### Nicolas Banholzer^1^, Stefan Feuerriegel^1^

###### ^1^Department of Management Information Systems, ETH Zurich, Weinbergstr. 56/58, 8092 Zurich, Switzerland

####### **Correspondence:** Nicolas Banholzer

**Background:** Health care data often has a hierarchical structure with observations at different group levels (e.g., regions, hospitals, patients). Most commonly applied statistical models either combine the data to estimate an average effect (complete pooling) or partition the data to estimate a separate effect for each group (no pooling). Such models treat the between-group variance implicitly as either zero (i.e., complete pooling) or infinity (i.e., no pooling).

We seek statistical models that balance the trade-off between zero and infinite between-group variance and, as a result, incorporate both between- and within-group information in the group-level estimates.

**Methods:** Bayesian hierarchical models account for the uncertainty in the estimate of the between-group variance through partial pooling. That is, group-level effects are estimated by taking into account the uncertainty about the estimates. For groups with few observations (or few information), the estimates are closer to the estimate from complete pooling. Instead, for groups with many observations, the estimates are closer to the estimate from no pooling. This principle is called shrinkage and can be thought of as pulling group-level estimates towards the population mean when uncertainty in the estimate is high.

**Results:** We apply Bayesian hierarchical models in different contexts of personalized health care. Thereby, hierarchical models reveal that patient-specific effects can be estimated precisely for patients with many observations, while the estimate for patients with few observations are pulled towards the patient-average.

**Conclusions:** Our applications demonstrate the advantages of Bayesian hierarchical models for personalized health care. Results from such models entail important implications for medical practitioners. They inform physicians about patients where personalized treatment is more likely to be successful and, vice versa, where a common treatment should rather be administered because the range of possible treatment effects is too large.

**Keywords:** Bayesian hierarchical models, patient-specific effects, personalized health care

#### 41. Dealing with multiple thresholds in diagnostic test accuracy meta-analysis: application of two modelling strategies

##### Hanne Ann Boon^1^, Thomas Struyf^1^, Dominique Bullens^2,3^, Ann Van den Bruel^1,4^ and Jan Y Verbakel^1,4^

###### ^1^Department of Public Health and Primary Care, KU Leuven, Leuven, Belgium; ^2^Department of Microbiology, Immunology and Transplantation, KU Leuven, Leuven, Belgium; ^3^Clinical division of pediatrics, UZ Leuven, Leuven, Belgium; ^4^Nuffield Department of Primary Care Health Sciences, University of Oxford, Oxford, UK

####### **Correspondence:** Hanne Ann Boon

**Background:** Dealing with multiple thresholds in diagnostic accuracy meta-analysis can be challenging. We applied two modelling strategies to summarize the available evidence of the diagnostic accuracy of biomarkers for urinary tract infections in children.

**Methods:** We performed a systematic review and meta-analysis of diagnostic test accuracy studies. We searched seven databases for relevant articles. Eligible studies were prospective or retrospective observational studies that reported the accuracy of urine or blood biomarkers for urinary tract infections in children. Statistical analyses were performed using R software. The bivariate random effects model by Reitsma et al. ^[1]^ (‘mada’ package) and the model by Steinhauser et al. ^[2]^ ( ‘diagmeta’ package), taking into account multiple thresholds per study, were both performed to calculate summary estimates for six biomarkers. We compared the output of two modelling strategies and reported the following characteristics: Area Under the Curve (AUC) and clinical usability (clinically relevant threshold providing a specificity of 0.90). For now, only results for C-reactive protein (CRP) are shown, with the other biomarkers to be presented at the MEMTAB 2020 symposium.

**Results:** We screened 9975 eligible studies, of which we included 62 in the review. For CRP, we found eight primary studies that reported on 1 to 6 thresholds, ranging from 5 to 200 mg/l. Using the model by Reitsma et al. ^[1]^ and Steinhauser et al. ^[2]^ the AUC was 0.705 (95%CI 0.581 – 0.812) and 0.748 (95%CI 0.617-0.856), respectively. To reach a specificity of 0.90 (diagnosing UTIs), the clinically relevant threshold was 25.33 mg/l, using the second method.

**Conclusions:** The ‘diagmeta’ package, implementing the method by Steinhauser et al., allows specification of clinically relevant thresholds according to the intended test aim. All primary study data, including all reported thresholds, can be implemented in the model, resulting in more reliable summary estimates.

**Keywords:** Meta-analysis, diagnostic, test, accuracy, biomarkers

**References:**

^[1]^ Reitsma JB, Glas AS, Rutjes AW, Scholten RJ, Bossuyt PM, Zwinderman AH. Bivariate analysis of sensitivity and specificity produces informative summary measures in diagnostic reviews. J Clin Epidemiol. 2005 Oct;58(10):982-90.

^[2]^ Steinhauser S, Schumacher M, Rücker G. Modelling multiple thresholds in meta-analysis of diagnostic test accuracy studies. BMC Med Res Methodol. 2016 Aug 12;16(1):97.

#### 42. Harnessing repeated measurements of predictor variables: A review of existing methods for clinical risk prediction

##### Lucy M. Bull^1,2^, Mark Lunt^1^, Glen P. Martin^3^, Kimme Hyrich^1,4^, Jamie C. Sergeant^1,2^

###### ^1^Centre for Epidemiology Versus Arthritis, Centre for Musculoskeletal Research, Manchester Academic Health Science Centre, University of Manchester, Oxford Road, Manchester M13 9PL, UK; ^2^Centre for Biostatistics, Manchester Academic Health Science Centre, University of Manchester, Oxford Road, Manchester M13 9PL, UK; ^3^Division of Informatics, Imaging and Data Science, Faculty of Biology, Medicine and Health, University of Manchester, Manchester Academic Health Science Centre, Oxford Road, Manchester M13 9PL, UK; ^4^National Institute for Health Research Manchester Biomedical Research Centre, Manchester University NHS Foundation Trust, Manchester Academic Health Science Centre, Oxford Road, Manchester M13 9PL, UK

####### **Correspondence:** Lucy M. Bull

**Background:** Clinical prediction models (CPMs) can predict the risk of health outcomes, such as disease onset or progression, for individual patients. The majority of existing CPMs only harness cross-sectional patient information. Incorporating repeated measurements into CPMs may provide an opportunity to enhance their performance.

We aimed to systematically review the literature to understand and summarise existing approaches for harnessing repeated measurements in the development of CPMs, and empirically investigate the suitability of identified methods to real-world data using an illustrative example in rheumatoid arthritis (RA).

**Methods:** Medline, Embase, and Web of Science were searched for articles reporting the development of a multivariable CPM for patient-level prediction, and modelling repeated measurements of at least one predictor. Information was extracted on: the method, its specific aim, reported advantages and limitations, and software available to apply the method. For the illustrative example, CPMs were developed to predict serious infections for RA patients starting anti-TNF therapy. Preliminary analyses include a comparison of compatible cross-sectional and longitudinal CPMs to predict a patient’s 12-month risk of serious infection at various time points during follow-up.

**Results:** The database search revealed 217 relevant articles. Seven methodological frameworks were identified: time-dependent covariate modelling, generalised estimating equations, landmark analysis, two-stage modelling, joint-modelling, trajectory classification and machine learning. Each of these frameworks satisfies at least one of three aims: to better specify predictor-outcome relationship over time; to infer a covariate value at a pre-specified time, and to account for the effect of covariate change. Identified features in available RA observational cohort data motivated the comparison of six applicable methods.

**Conclusions:** The applicability of identified methods depends on the motivation for including longitudinal information and the method’s compatibility with the clinical context and available patient data, for both model development and risk estimation in practice.

**Keywords:** Dynamic prediction, clinical prediction models, longitudinal data

#### 43. Adaptive sample size determination for the development of clinical prediction models

##### Evangelia Christodoulou^1^, Maarten van Smeden^2^, Dirk Timmerman^1,3^, Ewout Steyerberg^4^, Ben Van Calster^1, 4^

###### ^1^Department of Development & Regeneration, KU Leuven, Leuven, Belgium; ^2^Department of Clinical Epidemiology, Leiden University Medical Center, Leiden, The Netherlands; ^3^University Hospitals Leuven, Leuven, Belgium; ^4^Department of Biomedical Data Sciences, Leiden University Medical Center, Leiden, Netherlands

####### **Correspondence:** Evangelia Christodoulou

**Background:** For prediction model development, specifying an optimal sample size in terms of predictive performance is an active area of research. It is suggested that sample size depends on factors including event per variable (EPV), outcome prevalence and prevalence of binary predictors.

We introduce a flexible approach for sample size determination based on learning curves. Such curves monitor model performance as new data comes in, to allow stopping patient recruitment when a pre-specified stopping criterion has been reached. We illustrate the approach using data for the diagnosis of obstructive coronary artery disease (n=4888, 44% event rate).

**Methods:** We used logistic regression to develop prediction models consisting of a-priori selected variables. We mimicked prospective patient recruitment as follows. First, we fitted the model on 100 randomly chosen patients, and estimated model performance metrics using bootstrapping. Second, we sequentially added 50 random new patients until we reached 3000 patients, and estimated model performance at each step. We repeated the procedure 500 times to investigate variability. We built models once without addressing nonlinear effects of continuous predictors (ML-LR), and once with restricted cubic splines (RCS). We examined the required sample size for the following possible stopping criteria: (1) calibration slope (CS) 0.9, (2) CS 0.9 and c-statistic increase (Δc) <=0.01, (3) CS 0.9 and Δc<=0.01 for two consecutive sample sizes.

**Results:** When ML-LR was used, stopping criteria were met on average at sample sizes of 698 for criterion 1 (range 450-1000; EPV range 17-41), 1276 for criterion 2 (950-1550; 38-62), and 1368 (1050-1650; 41-66) for criterion 3. In contrast, EPV 10 was reached after 278 patients on average, with an average CS of 0.78. With RCS, the stopping criteria required 35%-41% more patients.

**Conclusions:** Learning curves are important instruments to tailor sample size to a specific context.

**Keywords:** Prediction model development, sample size, stopping criteria

#### 44. Propensity-based standardization to enhance the interpretation of predictive performance in individual participant data meta-analysis

##### Valentijn M.T. de Jong^1^, Jeroen Hoogland^1^, Karel G.M. Moons^1,2^, Tri-Long Nguyen^1,3,4^, Thomas P.A. Debray^1,2^

###### ^1^Julius Center for Health Sciences and Primary Care, University Medical Center Utrecht, Utrecht University, Utrecht, The Netherlands; ^2^ Cochrane Netherlands, University Medical Center Utrecht, Utrecht, The Netherlands; ^3^ Section of Epidemiology, Department of Public Health, University of Copenhagen, Copenhagen, Denmark; ^4^ Department of Pharmacy, University Hospital Centre of Nîmes and University of Montpellier, Nîmes, France

####### **Correspondence:** Valentijn M.T. de Jong

**Background:** Meta-analysis of individual participant data (IPD-MA) offers new opportunities for studying the generalizability of prediction models across different settings and populations. The interpretation of model performance estimates in IPD-MA is often challenging, because between-study heterogeneity may arise from invalid model coefficients and differences in (the distribution of) population characteristics. Hence, the benefit of local model revisions may be unclear.

We aimed to disentangle the effects of differences in case-mix and invalid regression coefficients, to allow for the identification of reproducibility of model performance and predictor effects.

**Methods:** We propose to standardize the c-statistic, calibration slope and calibration-in-the-large for case-mix differences between samples by applying propensity-weighting. The propensity scores are derived using a (multinomial) membership model that predicts the originating sample of an individual in the IPD-MA.

We illustrate our methods in a motivating example on the validation of eight diagnostic prediction models for detecting deep vein thrombosis (DVT) that may aid in the diagnosis of patients suspected of DVT in 12 external validation data sets. We analyze the estimates of prediction models’ performance across the external validation sets with random effects meta-analysis.

**Results:** In the meta-analysis of c-statistics, summary estimates were not affected much by standardization. However, standardization substantially reduced the between-study heterogeneity, indicating that variation of the models’ discrimination across the validation studies can partially be attributed to differences in case-mix, rather than invalid model coefficients.

Standardization increased the estimated between-study heterogeneity in calibration slopes. This implies that the predictor effects do not reproduce well in new samples with the same case-mix distribution.

**Conclusions:** Propensity score-based standardization may facilitate the interpretation of (heterogeneity in) prediction model performance across external validation studies, guide model updating strategies or show that the validation sample does not reflect the target population of the model.

**Keywords:** Prediction model, performance, propensity score, standardization, external validation

#### 45. Exploring test accuracy of faecal calprotectin for IBD using primary care electronic health records

##### Karoline Freeman^1^, Ronan Ryan^1^, Sian Taylor-Phillips^1,2^, Brian H. Willis^2^, Aileen Clarke^1^

###### ^1^Warwick Medical School, University of Warwick, Coventry, UK; ^2^Institute of Applied Health Research, University of Birmingham, Birmingham, UK

####### **Correspondence:** Karoline Freeman

**Background:** Test accuracy measures for primary care are often derived from small, heterogeneous studies suffering from differential verification bias.

We aimed to explore the potential of using routine primary care data to derive test accuracy estimates of faecal calprotectin (FC) testing for inflammatory bowel disease (IBD) compared to conventional and tailored meta-analyses of published test accuracy studies.

**Methods:** FC tests in adult patients with no previous IBD diagnosis from 2006-2016 were extracted from THIN. Multiple tests, tests without numeric results, with missing units or units other than μg/g were excluded. The reference standard was a coded record of IBD diagnosis or disease specific medication at three follow-up times. Sensitivity analyses explored assumptions on test exclusions, reference standard and patient selection. Results were compared to pooled estimates of sensitivity and specificity using conventional and tailored meta-analysis of studies from a recent systematic review.

**Results:** 7084/17466 FC tests were included. 4570 FC tests had no subsequent diagnosis recorded. Longer follow-up had no impact on the number of IBD diagnoses. The main methodological issues were 1) missing test results, 2) missing variables including indication for testing, test/laboratory information and results from secondary care testing, 3) misclassification of disease using clinical codes, 4) the inability to confirm absence of disease. Study assumptions had a greater impact on specificity than sensitivity. Sensitivity and specificity were similar to pooled estimates from meta-analyses (Table 1). The test positive rate was higher but IBD prevalence in FC tested patients was similar in routine data and published studies of similar settings.

**Table 1 (abstract 45).**
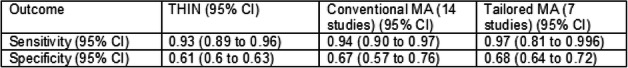
Sensitivity and specificity of FC testing using three methods

**Conclusions:** Test performance measures using routine data need to be interpreted with caution considering study limitations. Triangulation of tailored meta-analysis and routine data may provide evidence sufficient to support decision making.

**Keywords**: Electronic health records, test accuracy, tailored meta-analysis

#### 46. Multiple screening tools, multiple thresholds, multiple clinical cohorts: Evaluating screening tools for obstructive sleep apnoea

##### Suzanne C Freeman^1^, Emer M Brady^2^, Helena Polmann^3^, Jessica Réus^3^, Graziela De Luca Canto^3^, Noelle Robertson^4^, Iain Squire^5^, Lizelle Bernhardt^5,6^

###### ^1^ Department of Health Sciences, University of Leicester, Leicester, UK; ^2^ Diabetes Centre, University Hospitals of Leicester NHS Trust, Leicester, UK; ^3^ Brazilian Centre for Evidence-based Research/Centro Brasileiro de Pesquisas Baseadas em Evidências, Federal University of Santa Catarina, Florianopólis, Brazil; ^4^ Department of Neuroscience, Psychology and Behaviour, University of Leicester, Leicester, UK; ^5^ Department of Cardiovascular Sciences, University of Leicester, Leicester, UK; ^6^ Community Health Services, Leicestershire Partnership NHS Trust, Leicester, UK

####### **Correspondence:** Suzanne C Freeman

**Background:** In the UK, 1.4 million people live with undiagnosed and untreated obstructive sleep apnoea (OSA) and are at an increased risk of cardio-metabolic complications and diabetes. Polysomnography (PSG) is the gold standard for the diagnosis of OSA but is expensive, time-consuming and has long waiting lists. A questionnaire to identify patients at high risk of OSA requiring further investigation and treatment would be of great benefit.

We aimed to determine the best questionnaire for identifying adults at high risk of OSA amongst different clinical cohorts accounting for multiple questionnaires and multiple thresholds.

**Methods:** 31 studies reporting the diagnostic accuracy of the Berlin, STOP or STOP-Bang questionnaires as a screening tool for moderate-to-severe OSA were available for meta-analysis from two clinical cohorts of patients: sleep clinic and surgical. Within each cohort random effects bivariate binomial models were fitted to each questionnaire. Where there was a difference in diagnostic ability between questionnaires we tested this using meta-regression. In the surgical cohort, we accounted for multiple thresholds using the methods of Steinhauser et al^[1]^.

**Results:** In both the sleep clinic and surgical cohorts, meta-regression including questionnaire as a covariate identified statistical differences in sensitivity between STOP-Bang and Berlin. There was no evidence of differences in specificity. Due to the large number of parameters estimated when accounting for multiple thresholds we were only able to fit two of the eight models proposed by Steinhauser et al^[1]^.

**Conclusions:** Performing a coherent analysis under the frequentist framework that is able to incorporate multiple questionnaires and multiple thresholds across different clinical cohorts whilst avoiding the well-known issues associated with multiple testing can be challenging within the limits of current methodology, even with a moderately sized dataset.

**Keywords:** Diagnosis, meta-analysis, screening

**References**

[1] Steinhauser et al. Modelling multiple thresholds in meta-analysis of diagnostic test accuracy studies. BMC Med Res Meth 2016;16:97

#### 47. Only fools rush in! – initial data analysis is required for developing and validating prediction models

##### Georg Heinze^1^, Mark Baillie^2^, Marianne Huebner^3^

###### ^1^Medical University of Vienna, CeMSIIS - Section for Clinical Biometrics, Prognosis research group; Spitalgasse 23, 1090 Vienna, Austria; ^2^Biostatistical Sciences and Pharmacometrics, Novartis Pharma AG, Basel, Switzerland; ^3^Department of Statistics and Probability, Michigan State University, East Lansing, MI, USA

####### **Correspondence:** Georg Heinze

**Background:** In the age of personalized medicine, prediction models are becoming increasingly popular for risk stratification and informed treatment decisions. Accessibility of large routine data collections and observational cohorts facilitates the validation of existing prediction models and the development of new ones.

We aimed to define necessary steps and to stress the importance of initial data analysis before running regression analysis (IDA-REG), assuming that a data set has already passed an initial data cleaning stage.

**Methods:** Following a conceptional framework for IDA^[1]^, we describe 3 mandatory and 3 optional steps of IDA-REG.

**Results:** IDA-REG focuses on informing an analyst about features in the data that should be known to the data analyst in order to a) properly interpret results of an analysis, b) make decisions on how to present the results of an analysis, and c) adapt the statistical analysis plan to avoid analysis errors. Mandatory steps include summaries of univariate distributions of predictors and outcome variable, summaries of bi- and trivariate distributions of predictors, and summaries of patterns of missing values. Optional steps include investigation of measurement error, investigation of levels of measurement (hierarchies), and exploring unsupervised possibilities to reduce dimensionality of regression models. The evaluation of associations of predictors with the outcome is explicitly not part of an IDA-REG. We exemplify IDA-REG by means of simulated and real data.

**Conclusions:** Appropriate graphical and analytical tools enable a researcher to perform IDA-REG in order to avoid misinterpretation, poor presentation and analysis errors. These necessary preparations are too often forgotten by inexperienced data analysts. (253 < 300 words)

**Keywords:** Prediction, model, data screening

**References**

^[1]^ Huebner M, le Cessie S, Schmidt C, Vach W on behalf of the Topic Group “Initial Data Analysis” of the STRATOS Initiative: A Contemporary Conceptual Framework for Initial Data Analysis. Observational Studies 4 (2018):171-192.

#### 48. Pertussis in Belgium - The challenge of using historical serial serological survey data

##### Sereina A Herzog^1^, Steven Abrams^2,3^, Amber Litzroth^4^, Isabelle Desombere^5^, Heidi Theeten^1^, Niel Hens^1,2,3^

###### ^1^Vaccine and Infectious Disease Institute, University of Antwerp, Belgium; ^2^Department of Epidemiology and Social Medicine, University of Antwerp, Belgium; ^3^Data Science Institute, Hasselt University, Belgium; ^4^SD Epidemiology and Public Health, Sciensano, Brussels, Belgium; ^5^SD Infectious Diseases in Humans, Sciensano, Brussels, Belgium

####### **Correspondence:** Niel Hens

**Background:** Pertussis or whooping cough is a highly contagious vaccine preventable disease. Incidence of pertussis, has known a steady decline after the introduction of pertussis vaccination nevertheless pertussis incidence increased over the past two decades in many countries. The analysis of serial serological survey data can improve our understanding about the dynamics of pertussis. However, the development of assays for the detection of IgG antibodies in sera entails that various assays have been used for different survey years. We need comparable sero-epidemiological results for statistical and mathematical models to estimate time-varying epidemiological parameters.

We aimed to investigate the consequences of the uncertainty related to the standardization of pertussis toxin IgG antibodies results from three serological surveys conducted in Belgium (2002, 2006, 2013).

**Methods:** In each survey, 150 samples were selected such that the range of the original values for IgG antibodies against pertussis toxin was as best as possible covered. All 450 samples were then tested using a magnetic bead-based multiplex immunoassay (MIA).^[1]^ We investigated different models for the log-transformed values and considered also different strategies for outliers and censored data.

**Results:** The model choice for the standardization can be sensitive to the strategy applied for outliers and censored data. The survey 2013 was originally already tested using MIA but at different concentrations as the current study. The comparison with the re-tested 150 samples from 2013 together with validation data can be used to investigate intra-assay variability.

**Conclusions:** The uncertainty in the standardization of antibody titres needs to be reflected in models aimed at estimating time-varying epidemiological parameters, such as the force of infection, from serial serological survey data.

**Keywords:** Sero-epidemiology, pertussis, assay comparison

**References**

^[1]^ R. N. Caboré, D. Piérard, K. Huygen, Vaccines, 4 2016, 1-13.

#### 49. Diagnostic accuracy of C-reactive protein for appendicitis in primary care

##### Gea Holtman^1^, Guus Blok^1^, Eelke Nikkels^1^, Johan van der Lei^**2**^, Marjolein Berger^1^

###### ^1^ Department of General Practice and Elderly Care Medicine, University Medical Center Groningen, University of Groningen, PO Box 196, 9700 AD Groningen, The Netherlands; ^**2**^ Department of Medical Informatics, Erasmus Medical Center, Rotterdam, The Netherlands

####### **Correspondence:** Gea Holtman

**Background:** The diagnostic value of C-reactive protein (CRP) for appendicitis has been extensively evaluated in specialist care, but not in primary care.

We aimed to determine the (added) diagnostic value of CRP for appendicitis in children with acute abdominal pain in primary care.

**Methods:** A retrospective cohort study of children aged 4-18 years who presented in general practices with acute abdominal pain and a CRP test, between 2010 and 2016. CRP levels at first contact were compared to the final diagnosis of appendicitis reported in the specialist reports within six weeks. Test characteristics of CRP were calculated for multiple thresholds. To evaluate the added value to history and physical exam we compared the area under the receiver operating characteristics curves (AUC) and decision curves of two logistic regression models: 1) basic model (six clinical features assessed by the GP) and 2) basic model plus CRP.

**Results:** Of 1076 included patients, 203 (19%) were referred to specialist care and 70 (7%) had appendicitis. The sensitivity and specificity of CRP for a commonly used threshold of 10 mg/L was 0.87 (0.77-0.94) and 0.77 (0.74-0.79) respectively. The sensitivity increased to 1.00 (0.89-1.00) when the symptoms had lasted longer than 48 hours. Adding CRP to the basic model of clinical features increased the AUC significantly from 0.81 (0.76-0.85) to 0.88 (0.84-0.91). The decision curve showed that the basic model plus CRP had the highest net benefit at reasonable threshold probabilities (Figure 1).

**Conclusions:** In primary care, CRP showed to have an added value to history and physical examination in the diagnostic work-up of appendicitis in children with acute abdominal pain. A value below 10 mg/L in children with symptoms longer than 48 hours could safely rule out appendicitis.

**Fig. 1 (abstract 49).**
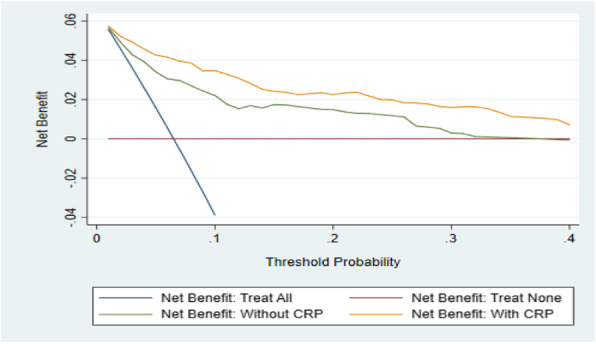
Decision curve for both models predicting appendicitis.

**Keywords:** CRP, appendicitis, primary care

#### 50. Developing and validating a warfarin dose prediction model for patients in sub-Saharan Africa

##### Innocent G Asiimwe^1^, Munir Pirmohamed^1^, Andrea L Jorgensen^2^

###### ^1^The Wolfson Centre for Personalised Medicine, MRC Centre for Drug Safety Science, Department of Molecular and Clinical Pharmacology, University of Liverpool. University of Liverpool, UK; ^2^Department of Biostatistics, University of Liverpool, UK

####### **Correspondence:** Andrea L Jorgensen

**Background:** Warfarin remains the most used oral anticoagulant in sub-Saharan Africa. It has a narrow therapeutic index and highly variable clinical response for a given dose and thus optimal dose prediction is difficult.

We aimed to develop and validate a warfarin dose prediction model for use in sub-Saharan African populations.

**Methods:** Multivariable linear regression models were fitted using data from 364 patients. Starting with a list of potential variables, all possible linear models were fitted, with the optimal models chosen with reference to mean absolute error (MAE), mean absolute percentage error (MAPE) and logarithmic accuracy ratio. Bootstrap validation was applied to correct overfitting and the final models were externally validated in a cohort of 690 patients. In both development and external validation cohorts, we compared our models with current warfarin initiation practice (fixed dose of 35 mg/week) and two widely known dose prediction models.

**Results:** The final model included the three predictor variables age, weight and target International Normalized Ratio, and gave MAE of 11.7 (95% CI, 10.5-12.9) mg/wk, MAPE of 14.4% and a log accuracy ratio of 0.003. Ideal dose (predicted dose within 20% of actual dose) was achieved in 42.6% patients. In external validation, MAE was 12.4 (11.5-13.5) mg/wk, MAPE 14.7%, log accuracy ratio 0.006 and ideal dose was achieved in 41.4%. Based on all these metrics, our model performed better than the two well-known models, and compared to fixed dosing, it decreased the percentage of patients at high risk of sub-optimal anticoagulation by 8.3% and 11.9% in the development and validation cohorts respectively.

**Conclusions:** A dosing model has been developed for the first time for Black African patients starting warfarin in South Africa and Uganda. Its clinical utility is soon to be tested in a prospective study.

**Keywords:** Dose prediction, warfarin, anticoagulation, Black African, personalized medicine

#### 51. Collinearity in prognostic models for dysphagia

##### Artuur Leeuwenberg, Ewoud Schuit, Johannes B. Reitsma, Karel G. Moons

###### Julius Center for Health Sciences and Primary Care, University Medical Center Utrecht, 3508 GA, Utrecht, The Netherlands

####### **Correspondence:** Artuur Leeuwenberg

**Background:** Normal Tissue Complication Probability (NTCP) models predict the risk of radiation induced complications and can be used to optimize dosage plans of radiation-based therapies to minimize the risk of such complications. Dosage delivered to organs at risk (OARs) surrounding the targeted tumor is often highly collinear (r^2^ > 0.8), inducing high variance on coefficient estimates. Consequently, many NTCP models are developed using only a subset of relevant OAR, especially for complex complications, like dysphagia, involving many relevant OAR. Excluding OAR can be problematic when using NTCP models for dosage optimization as it steers dosage towards excluded OAR.

We aimed to compare different methods to address collinearity without degradation of model performance.

**Methods:** We compared five methods that constrain the coefficient search space (reducing coefficient variance) for standard logistic regression: Lasso, Ridge, principal component regression, dropout regularization (random dropout of predictors during iterative model fitting), and non-negativity constraints for OAR dosage coefficients. Each method is empirically evaluated, using a 16-predictor logistic regression NTCP model for dysphagia grade ≥2 (predictors are: primary tumor location, and mean dose for 11 OARs).

**Table 1 (abstract 51).**
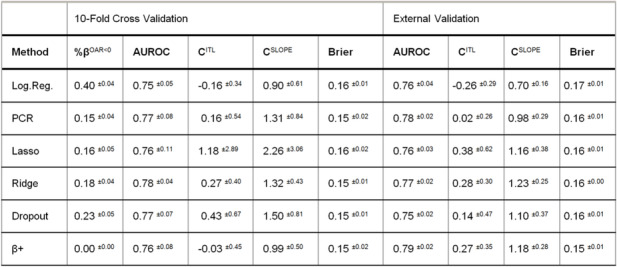
Comparison of methods for a within-hospital 10-fold-cross-validation (n=489) and an external validation (n^dev^=489, n^test^=143). Reported statistics (and standard deviations) are: the percentage of negative OAR coefficients (%β^OAR<0^), area under the receiver-operating-characteristic curve (AUROC), calibration in-the-large (C^ITL^), calibration slope (C^SLOPE^), and Brier score.

**Results & Conclusions:** Table 1 shows similar AUROC and Brier across methods, and similar calibration (in-the-large and slope) at cross-validation. PCA and non-negativity constraints stand out in terms of calibration at external validation. All methods reduce but still contain one or more negative coefficients for OAR dosage variables except when using non-negativity constraints (β+). If accepted for presentation, we expect to show results of combining PCA and non-negativity constraints as well, as we concluded these methods to be most beneficial for this use case based on these preliminary results.

**Keywords:** Collinearity, NTCP, radiotherapy, prediction, dysphagia

#### 52. Evaluating risk of bias and applicability in meta-analyses of individual participant data

##### Brooke Levis^1^, Kym IE Snell^1^, Yemisi Takwoingi^2^, Gary S Collins^3^, Karel G Moons^4^, Johannes B Reitsma^4^, Lotty Hooft^4^, and Richard D Riley^1^

###### ^1^Centre for Prognosis Research, School of Primary, Community and Social Care, Keele University, Staffordshire, UK; ^2^Test Evaluation Research Group, Institute of Applied Health Research, University of Birmingham, Birmingham, UK; ^3^Centre for Statistics in Medicine, Nuffield Department of Orthopaedics, Rheumatology and Musculoskeletal Sciences, University of Oxford, UK; ^4^Julius Center for Health Sciences and Primary Care, University Medical Center Utrecht, Utrecht, The Netherlands

####### **Correspondence:** Brooke Levis

**Background:** Assessing risk of bias and applicability (RoB) of included studies is critical for interpreting meta-analysis (MA) results. RoB tools for diagnostic, prognostic, and prediction studies include QUADAS-2 and PROBAST. However, individual participant data meta-analyses (IPD-MAs) differ from aggregate-data MAs in that in IPD-MA, datasets may include additional information, eligibility criteria may differ from the original publications, and definitions for index tests/predictors and reference standards/outcomes can be standardized across studies. Thus, tailored RoB tools may be needed.

We aimed to review how RoB is currently assessed in IPD-MAs, and to examine QUADAS-2 and PROBAST, with the goal of developing IPD-MA extensions for each tool.

**Methods:** We reviewed RoB assessments in IPD-MAs published in the last 12 months. We then examined how QUADAS-2 (and in-progress extensions) and PROBAST items might be evaluated in an IPD-MA context; noting which items might be removed, edited, or added; and hypothesized how results may be incorporated into IPD-MA analyses.

**Results:** We observed that current IPD-MAs rarely and inconsistently evaluate RoB, and most do not incorporate RoB judgements into analyses. Our findings indicate using QUADAS-2 and PROBAST to assess RoB of IPD datasets themselves, rather than study publications. Certain items may need to be coded at the participant level (e.g., timing between index test/predictor and reference standard/outcome), whereas others (e.g., quality of diagnostic tool) may apply uniformly to an included study. Most analysis items (e.g., pre-specification of thresholds and variables for analysis) may not be relevant, as IPD-MA researchers perform the analyses themselves. RoB results may be incorporated into analyses by conducting subgroup analyses among studies and participants with overall low RoB or by conducting formal interaction analyses with item-level RoB responses.

**Conclusions:** Development and dissemination of IPD-MA extensions for QUADAS-2 and PROBAST will lead to improved RoB assessments in IPD-MAs of diagnostic, prognostic, and prediction studies.

**Keywords:** Risk of bias, applicability, QUADAS-2, PROBAST, individual participant data meta-analysis

#### 53. Selective Cutoff Reporting in Diagnostic Accuracy Studies of the PHQ-9 and EPDS Depression Screening Tools

##### Dipika Neupane^1,2^, Brooke Levis^1,2,3^, Parash Mani Bhandari^1,2^, Brett D Thombs^1,2^, Andrea Benedetti^2,4^, and the DEPRESsion Screening Data (DEPRESSD) Collaboration

###### ^1^Lady Davis Institute for Medical Research, Jewish General Hospital, Montréal, Québec, Canada; ^2^Department of Epidemiology, Biostatistics and Occupational Health, McGill University, Montréal, Québec, Canada; ^3^Centre for Prognosis Research, School of Primary, Community and Social Care, Keele University, Staffordshire, UK; ^4^Respiratory Epidemiology and Clinical Research Unit, McGill University Health Centre, Montréal, Québec, Canada

####### **Correspondence:** Brooke Levis

**Background:** Selectively reporting accuracy results from only well-performing cutoffs in studies of diagnostic or screening tests may result in biased estimates when synthesized. Extent of bias may differ depending on the availability of a well-defined standard cutoff.

We compared bias in accuracy estimates and cutoff reporting patterns for the Patient Health Questionnaire-9 (PHQ-9; well-defined standard cutoff ≥10) and Edinburgh Postnatal Depression Scale (EPDS; no standard cutoff, common cutoffs ≥10 to ≥13).

**Methods:** We analyzed subsets of datasets from two separate individual participant data meta-analyses (IPDMAs) on PHQ-9 and EPDS accuracy. Separately, for the PHQ-9 and EPDS, we used bivariate random effects meta-analysis to compare accuracy estimates based on published cutoffs only versus all cutoffs from all studies. We also compared the number of published cutoffs below and above the standard or common cutoffs in relation to study-specific “optimal” cutoffs.

**Results:** For the PHQ-9 (30 studies, N = 11,773), published results underestimated sensitivity compared to results for all cutoffs for cutoffs below ≥10 (median difference: -0.06) and overestimated for cutoffs above ≥10 (median difference: 0.07). EPDS (19 studies, N = 3,637) sensitivity estimates were similar for cutoffs below ≥10 (median difference: 0.01) but higher for published cutoffs above ≥13 (median difference: 0.14). Mean cutoff of all cutoffs reported among PHQ-9 studies with optimal cutoffs below ≥10 was 8.8 compared to 11.8 for studies with optimal cutoffs above ≥10. 18 of 19 EPDS studies had optimal cutoffs below ≥13; those below ≥10 did not report more cutoffs below ≥10 (mean cutoff: 9.9), but those with above ≥10 reported more above ≥10 (mean cutoff: 11.8).

**Conclusions:** Selective cutoff reporting and resulting bias in accuracy estimates were more pronounced for the PHQ-9 than EPDS. Researchers evaluating diagnostic accuracy of screening tools should report results for all relevant cutoffs.

**Keywords:** diagnostic test accuracy, individual participant data meta-analysis, meta-analysis, selective cutoff reporting, publication bias

#### 54. Estimating the prevalence of misdiagnosis of giant cell arteritis: using a genetic test as umpire

##### Charikleia Chatzigeorgiou^1^, Jennifer H Barrett^1,2^, Javier Martin^3^, Ann W Morgan^1,2^, Sarah L Mackie^1,2^

###### ^1^School of Medicine, University of Leeds, Leeds, UK; ^2^NIHR Leeds Biomedical Research Centre, Leeds Teaching Hospitals NHS Trust, Leeds, UK; ^3^Instituo di Parasitologia y Biomedicina, Consejo Superior de Investigaciones Cientificas (Spanish National Research Council), Madrid, Spain

####### **Correspondence:** Sarah L Mackie

**Background:** The diagnosis of giant cell arteritis (GCA) can be confirmed by temporal artery biopsy (TAB). However, TAB is insensitive; therefore, GCA is often diagnosed on clinical grounds despite negative TAB. The prevalence of misdiagnosis in this patient group is unknown. GCA has a strong HLA genetic association [1] that might be used as an umpire test.

We aimed to estimate the prevalence of misdiagnosis of GCA among patients diagnosed with GCA without a positive TAB.

**Methods:** Cases came from UK GCA Consortium, which recruited patients with a firm clinical diagnosis of GCA. Population control data came from the Wellcome Trust Case Control Consortium. Cases were genotyped using an Illumina genotyping chip [1]. Case and control genomes were jointly imputed using SNP2HLA. A genetic association analysis was carried out, adjusting for the first ten principal components. Misdiagnosis rate was estimated using observed frequencies of nominally-associated variants in the HLA region (P<0.1), assuming the GCA patient group was composed of a mixture of genuine GCA cases and misdiagnosed cases.

**Results:** 663 patients diagnosed with GCA (356 with a positive TAB, 147 with a negative TAB and 160 with no TAB result) were compared with 2619 controls. Allele frequencies of 470 variants in the HLA region were compared. The estimated proportion of patients misdiagnosed as GCA was 67% in the negative-TAB group and 33% in the group without TAB result.

**Conclusions:** The proportion of patients misdiagnosed with GCA can be estimated under certain assumptions. We assumed accurate reporting of TAB and that the cases with genuine GCA with and without a positive TAB are genetically similar. This method could be extended to similar diseases with an insensitive but highly specific reference-standard test and strong genetic susceptibility associations.

**Keywords:** Imperfect reference standard, umpire test, misdiagnosis, genetic association

**References**

^[1]^ Carmona et al., *Am J Hum Genet* 100 2017, 64-74.

#### 55. Bayesian latent class analysis versus composite reference standards for estimating tuberculosis meningitis diagnostic test accuracy

##### Emily MacLean^1,2^, Mikashmi Kohli^2^, Nandini Dendukuri^1,2^

###### ^1^Department of Epidemiology, Biostatistics, and Occupational Health, McGill University; 1020 av des pins ouest, H3A 1A2, Montreal, Quebec, Canada; ^2^McGill International TB Centre, Research Institute of the McGill University Health Centre; 1001 rue Decarie, Montreal, Quebec, H4A 3J1 Canada

####### **Correspondence:** Emily MacLean

**Background:** Tuberculosis meningitis (TBM) represents 2-5% of the global annual TB burden, or 0.2-0.5 million cases^[1]^, a highly uncertain prevalence estimate as there is no reliable gold standard to definitively classify it. Despite recognition of the imperfect nature of employed reference standards, naïve methods used to evaluate accuracy of new TBM tests, e.g. composite reference standards (CRS), do not account for the uncertainty in their accuracy. Consequently, estimates of TBM diagnostic test sensitivity and specificity based on such methods may be biased.

We used Bayesian latent class analysis to estimate Xpert MTB/RIF (Xpert) accuracies for diagnosing TBM and compared these to estimates from multiple CRSs.

**Methods:** An existing dataset of all adults presenting to a tertiary care hospital with suspected extrapulmonary tuberculosis in New Delhi, India, in 2012 was analysed. We selected individuals undergoing investigation for TBM with valid results for bacterial culture, smear microscopy, cytopathology/histopathology, and Xpert. A heuristic model was created to understand relationships between latent classes and tests, with a random effect to denote bacterial burden (Figure 1). A Bayesian approach was used to estimate the latent class model. Multiple CRSs were defined by increasing numbers of positive component tests. Analyses were performed using RJAGS (Version 4-8) through R-studio (3.5.2).

**Results:** Using 224 patients with suspected TBM, Xpert sensitivity and specificity were 51.2% (95%CrI:34.2-71.0) and 99.6% (95%CrI:97.7-100). Xpert sensitivity varied dramatically by CRS definition: sensitivity was 33% (95%CI:98-100) with a CRS of any one positive test result, increasing to 52% (95%CI:34-69) with two positive results, and reaching 100% (95%CI:48-100) with all four positive results.

**Conclusions:** Unlike CRSs, Bayesian latent class analysis produces estimated test accuracies that incorporate reference standard uncertainty and conditional dependence for TBM, the most severe form of tuberculosis.

**Fig. 1 (abstract 55).**
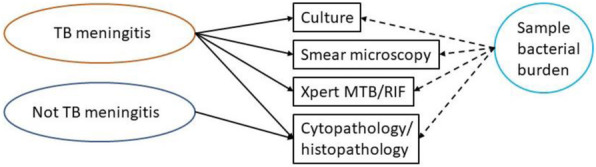
Heuristic model for TBM showing assumed relationships between latent classes (ovals), diagnostic test results (rectangles), and random effect (circle).

**Keywords:** Latent class analysis, diagnostics, tuberculosis meningitis, composite reference standards

**References**

^[1]^ World Health Organization, *Global tuberculosis report*, 2019, Geneva: World Health Organization.

#### 56. Measuring the impact of diagnostic tests on patient management decisions within three clinical trials

##### Sue Mallett^1^, Stuart A. Taylor^2^, Gauraang Batnagar^2^, STREAMLINE COLON Investigators, STREAMLINE LUNG Investigators, METRIC Investigators

###### ^1^Institute of Applied Health Research, University of Birmingham, Birmingham UK B15 2TT; ^2^Centre for Medical Imaging, University College London, Charles Bell House, 43-45 Foley Street, W1W 7TS, UK

####### **Correspondence:** Sue Mallett

**Background:** Standard studies comparing diagnostic tests measure diagnostic test accuracy. Some trials also provide information on additional outcomes such as time to diagnosis and the number of additional tests in patient pathway. Ideally diagnostic tests would be compared as interventions in randomised controlled trials (RCTs). However RCTs for comparison of diagnostic tests as interventions can be problematic to design and run. Problems include long time periods required for studies following patient outcomes during which either test or treatment pathways change, high numbers of patients required, high costs, ethical issues about randomizing to receive tests, difficulty understanding role of diagnostic test as complex intervention, plus other barriers. We present three examples where we have measured how tests affect patient management decisions within diagnostic accuracy trials.

We aimed to describe methods and insight from three clinical trials recently completed measuring the impact of diagnostic tests on patient management.

**Methods:** Three trials, each comparing alternative diagnostic tests or diagnostic test pathways against a reference standard of normal clinical practice have been designed to collect patient management decisions. In each patient management decisions based on the alternative pathways are reported based on eight or ten alternative management options. STREAMLINE COLON and LUNG compare whole body MRI to current NICE recommended pathways for detection of metastases at diagnosis of colon and lung cancer respectively. METRIC compares ultrasound and MRI for diagnosing the extent and activity of Crohn’s disease in newly diagnosed and relapsed patients.

**Results and discussion:** Additional analysis, subsequent to prior main trial results, are ongoing to explore impact of patient management decisions by linking detailed analysis of patient diagnosis and management decisions. These three trials provide insight into the design, analysis issues and how measuring patient management decisions in a clinical trial can provide important information on the role and uses of diagnostic tests.

**Keywords:** Diagnosis, impact, patient management, accuracy, clinical trial

#### 57. Ambiguous baseline definitions affect automated AKI diagnosis at emergency department

##### Michael S A Niemantsverdriet^1,2^, Meriem Khairoun^3^, Ayman El Idrissi^3^, Romy R Koopsen^3^, Imo E Hofer^1^, Wouter W Van Solinge^1^, Jan Willem Uffen^3^, Wouter M Tiel-Groenestege^1^, Karin A H Kaasjager^3^, Saskia Haitjema^1^

###### ^1^Central Diagnostic Laboratory, University Medical Center Utrecht, Utrecht University, Heidelberglaan 100, 3584 CX Utrecht, The Netherlands; ^2^SkylineDx, Lichtenauerlaan 40, 3062 ME Rotterdam, The Netherlands; ^3^Department of Internal Medicine, University Medical Center Utrecht, Utrecht University, Heidelberglaan 100, 3584 CX Utrecht, The Netherlands

####### **Correspondence:** Michael S A Niemantsverdriet

**Background:** Incorrect labeling of patients can hamper model development resulting in sub-optimal clinical decision support systems (CDSS) that can misclassify patients. During model development, patients are labeled, using guidelines postulated in literature, based on their retrospective routine electronic health record (EHR) data. Acute kidney injury (AKI) is identified by the Kidney Disease Improving Global Outcomes (KDIGO) criteria using changes in serum creatinine measurements. Creatinine measured at the emergency department (ED) is compared to a `baseline` measurement extracted from the patient's medical history. Depending on the definition of 'baseline' patients can be misclassified by the model. We evaluated the effect of multiple baseline-definitions on AKI prevalence in the ED.

**Methods:** 47.190 ED-visits (19.956 patients) in the UMC Utrecht with a prior creatinine measurement between 2011-2019 were included from the Utrecht Patient-Orientated Database. An increase of 26,5 μmol/L creatinine between baseline-value and the ED-value was used as AKI definition (KDIGO). We analyzed four baseline-definitions: lowest, mean, median and most recent value from the patient’s EHR. Multiple time intervals were used (≤365 days prior ED-presentation) to determine AKI-prevalence.

**Results:** The longest interval (365 days prior presentation) in combination with the lowest value as baseline resulted in the highest AKI-prevalence (12,65%) compared to the mean (4,23%), median (4,8%) and the most recent value (4,5%). Iteratively reducing the time window for extracting the creatinine measurement only showed extreme differences when using the lowest value as baseline. In comparison with the shortest interval (45 days) the longest interval increased the prevalence with 10,92% (5.151/47.190 additional AKI labels).

**Conclusions:** Using a specific definition of baseline, results in significantly different AKI prevalence in the ED. Adequate translation of guidelines to diagnose disease is crucial for accurate patient labeling to reduce misclassification by the model and to improve CDSS's accuracy to better support clinical decision making by treating physicians.

**Keywords:** Acute kidney injury, electronic health records, outcome

#### 58. Real-time handling of Missing Predictor Values when implementing and using prediction models in daily practice

##### Steven WJ Nijman^1^, T Katrien J Groenhof^1^, Jeroen Hoogland^1^, Michiel L Bots^1^, Menno Brandjes^2^, John JL Jacobs^2^, Folkert W Asselbergs^3,4,5^, Karel GM Moons^1^, Thomas PA Debray^1,4^

###### ^1^Julius Center for Health Sciences and Primary Care, Utrecht University, Utrecht, The Netherlands; ^2^LogiqCare, Ortec B.V. Zoetermeer, The Netherlands; ^3^Department of Cardiology, Division Heart & Lungs, University Medical Center Utrecht, Utrecht University, Utrecht, The Netherlands; ^4^Institute of Cardiovascular Science, Faculty of Population Health Sciences, University College London, London, UK; ^5^Health Data Research UK, Institute of Health Informatics, University College London, London, UK

####### **Correspondence:** Steven WJ Nijman

**Background:** Using prediction models to calculate a patients individual risk in clinical practice, requires complete information on all predictors in the prediction model. Unfortunately, routine care data is often incomplete due to a variety of reasons. Although several methods for real-time imputation of missing predictor values exist, they often require immediate access to data from other similar patients and are therefore not directly suitable for routine care.

We aimed to develop and evaluate methods for real-time imputation of missing predictor values in routine clinical care when applying prediction models to individual patients.

**Methods:** We describe (i) mean imputation (where missing values are replaced by the sample mean), (ii) joint modeling imputation (JMI, where we use a multivariate normal approximation to generate patient-specific imputations) and (iii) conditional modeling imputation (CMI, where a multivariable imputation model is derived for each predictor from a population). We compared the imputation methods by applying a previously developed prediction model (predicting 10-year risk of recurrent vascular disease) in a dataset with 3,880 participants from the Utrecht Cardiovascular Cohort in which missing predictor values were simulated. Furthermore, comparing true and imputed predictor values, the root mean squared error (RMSE) and coverage of the 95% confidence intervals (i.e. the proportion of confidence intervals that contain the true predictor value) were evaluated.

**Results:** We found that RMSE was lowest when adopting JMI or CMI, although imputation of individual predictors did not always lead to substantial accuracy improvements with regards to the RMSE, as compared to mean imputation. JMI and CMI appeared particularly useful when the values of multiple predictors of the model were missing. Coverage reached the nominal level (i.e. 95%) for both CMI and JMI.

**Conclusions:** Multiple imputation using, either CMI or JMI, is recommended when dealing with missing predictor values in real time settings.

**Keywords:** Missing data, multiple imputation, real-time imputation, prediction, decision support system, electronic health care records

#### 59. intelligent Liver Function Testing (iLFT): an algorithm-based pathway to increase diagnosis of liver disease

##### Jennifer Nobes^1^, Iain Macpherson^1,2^, Ellie Dow^1^, Ian Kennedy^1^, Michael Miller^1^, Elizabeth Furrie^1^, John Dillon^2^

###### ^1^NHS Tayside, Ninewells Hospital and Medical School, Dundee, DD1 9SY, UK; ^2^University of Dundee, Ninewells Hospital and Medical School, Dundee, DD1 9SY, UK

####### **Correspondence:** Jennifer Nobes; Iain Macpherson

**Background:** Mortality from chronic liver disease (CLD) is rising. This is despite ‘early warning’ from commonly requested liver function tests (LFTs) which are abnormal in around 20% of cases, providing a clear opportunity for earlier diagnosis and intervention. Intelligent liver function testing (iLFT) is a revolutionary system which aims to increase early diagnosis of CLD. The referring clinician provides information on alcohol intake and co-morbidities, allowing an automated algorithm to reflex relevant tests without further venepuncture when initial LFTs are abnormal. Recommended outcomes are then provided: secondary care referral; primary care follow-up; or further investigations and referral criteria. This replaces the current, protracted system in which tests are often repeated over many years before diagnosing irreversible liver cirrhosis. iLFT is cost-effective and provides a window of opportunity for lifestyle modification and treatment.

We aimed to improve healthcare by identifying an appropriate care pathway for individual patients, utilise the existing potential of equipment and working practices, and improve service access to Hepatology, ensuring appropriate patients are seen by specialists.

**Methods:** A retrospective analysis was performed of iLFT requests and results in the first year, and a user questionnaire was analysed.

***Results:*** 2362 iLFT requests were received over 12 months, identifying 509 patients with advanced CLD requiring secondary care review, and 1504 patients with early CLD in whom lifestyle modifications could prevent disease progression. The proportion of liver testing made up by iLFT increased month-on-month; iLFT now accounts for 3% of monthly LFTs. 98 of 100 local General Practitioners surveyed would recommend iLFT to colleagues.

**Conclusions:** iLFT is a successful system which utilises currently available resources to increase the diagnosis of CLD and provide appropriate referral advice. This creates a means to manage the growing healthcare burden from CLD and allows access to specialist care for appropriate patients.

**Keywords:** Liver, algorithm, cirrhosis, fibrosis, primary care, diagnosis

#### 60. Recommended labels for approaches to evaluate diagnostic accuracy: the STARD ReLabel project

##### Maria Olsen^1^, Bada Yang^1^, Patrick Bossuyt^1^ and Chris Hyde^2^

###### ^1^Amsterdam University Medical Centers, dept. of Clinical Epidemiology, Biostatistics and Bioinformatics, Amsterdam Public Health Research Institute, Meibergdreef 9, 1105 AZ Amsterdam, The Netherlands; ^2^Exeter Test Group, Institute of Health and Research, College of Medicine and Health, University of Exeter, UK

####### **Correspondence:** Maria Olsen

**Background**: There is no standardized terminology for describing diagnostic test accuracy (DTA) studies, which presents a barrier to clear and informative reporting of primary studies and hinders efforts towards making valid evidence synthesis. In a previous project, we observed a heterogeneous and sometimes confusing use of terminology for describing DTA study design features in reviews prepared for NICE guidelines^[1]^.

We aimed to develop a coherent set of terms for describing DTA study design features.

**Methods**: Based on data from our previous study, and newly collected data on features and terms, we are performing an iterative clarification, sorting, and categorization of all the terms and features we identified. These will be integrated in a coherent and complete set of terms, as a prototype. The strengths and limitations of this prototype are evaluated through an electronic survey. Participants are experienced DTA researchers and non-academic stakeholders and include health technology assessment groups, DTA guideline developers, and collaborators from industry. The survey responses are used to adapt and modify the set of terms. In the last phase, the set of terms will be piloted among end users with varying levels of DTA experience, to evaluate if it facilitates informative descriptions of DTA study designs.

**Results:** Our set of terms, developed with the input from a large group of experts and stakeholders, can be used to describe a DTA study in sufficient detail, without ambiguity.

**Conclusion:** We believe that having a standardized and agreed upon set of terms can reduce the use of misleading, subjective, ambiguous and heterogeneous wording when describing DTA research. This will eventually enable secondary researchers and health care decision-makers to better assess the validity and generalizability of DTA evidence.

**Keywords:** Diagnostic test accuracy, study designs, terminology, labelling

#### 61. Frequencies and patterns of microbiology test requests from general practice

##### José M. Ordóñez-Mena^1,2^, Thomas R. Fanshawe^1^, Dona Foster^3^, Sarah Walker^2^, Gail Hayward^1^

###### ^1^Nuffield Department of Primary Care Health Sciences, University of Oxford, Oxford, UK; ^2^NIHR Oxford Biomedical Research Centre, Oxford University Hospitals NHS Foundation Trust, Oxford, UK; ^3^Nuffield Department of Medicine, University of Oxford, Oxford, UK

####### **Correspondence:** José M. Ordóñez-Mena

**Background:** Microbiological tests requested from primary care are currently almost entirely performed in a central NHS laboratory. New diagnostic technologies allowing results to be available at the point of prescription could contribute to antimicrobial stewardship.

We aimed to quantify the demand for microbiology tests in primary care and highlight the most important individual and combinations of tests, and pathogens to inform the development of new single and multiplexed point-of-care tests.

**Methods:** A retrospective cohort of all Oxfordshire primary care patients for whom a microbiology test was requested between 2008-2018. We described test frequencies overall, positive test results, pathogens identified, and trends over time. We also investigated patterns of co-testing in the same and subsequent visits with heat-maps and hierarchical cluster analysis overall and in sex and age categories.

**Results:** 1,596,752 microbiology tests were requested for 393,905 patients of which 65.3% were women and 48.8% aged 18-49 years old. We organized individual tests into 19 microbiology test groups, 8 combined cultures and microscopies, and 11 related to individual pathogens. Urine cultures and microscopies (n=673,612) accounted for 42% of all microbiology tests and were mainly requested in isolation but also in follow-up visits after 7 and 14 days. Of all urine cultures, 27 % were positive and 26% had equivocal results. E. coli was the most prevalent pathogen in urine cultures (65.2%). Antenatal urine cultures and blood tests (Hepatitis B, HIV, Syphilis, and Rubella) formed the most common combination of tests particularly among women aged 18-49.

**Conclusions:** The greatest burden of microbiology testing in primary care can be attributable to urine cultures. Antenatal urine and blood tests done in women aged 18-49 are also a significant contributor to the burden of microbiology testing. Further research should focus on the impact of the development of point-of-care tests on these care pathways.

**Keywords:** Microbiology, primary care, testing

#### 62. Patient and public involvement in methodological research: a case study

##### Laura Quinn^1,2^, Alice Sitch^1,2^, Jon Deeks^1,2^

###### ^1^Test Evaluation Research Group, Institute of Applied Health Research, University of Birmingham, Edgbaston, Birmingham, UK; ^2^NIHR Birmingham Biomedical Research Centre, University Hospitals Birmingham NHS Foundation Trust and University of Birmingham, Birmingham, UK

####### **Correspondence:** Laura Quinn

**Background:** Public and patient involvement (PPI) in medical research is defined as research carried out “with” or “by” members of the public rather than “to,” “about” or “for” them ^[1]^. PPI is a key part of medical research, with many national health and funding organisations stating PPI is essential including bodies from the UK, Netherlands, America, Canada and USA ^[2]^. There is little information on how to integrate PPI into methodological research and the stakeholders that should be considered as public contributors.

We aimed to provide information for PPI involvement in methodological research, including available resources and present a case study.

**Methods:** As a case study, we describe a methodological research fellowship focusing on methods for determining the performance of diagnostic imaging tests by including information on interobserver variability and time to diagnosis. Within this project, we consulted with colleagues with experience integrating PPI into their methodological research, PPI leads from local hospitals and research centres, presented the research proposal to a PPI group for feedback and developed an integrated PPI approach.

**Results:** A description of the integrated PPI involvement for a methodological research fellowship and list of resources available for guidance. Some of the online resources include the INVOLVE National Standards for Public Involvement and cost calculator ^[3.4]^. Other resources include links to toolkits and useful papers on public involvement.

**Conclusions:** Investigators should plan PPI involvement in advance, research available help in local area including colleagues, PPI leads, and online support.

**Keywords:** PPI, methodological, research

**References**

^[1]^ INVOLVE – What is public involvement in research? http://www.invo.org.uk/find-out-more/what-is-public-involvement-in-research-2/. Accessed 14/01/2020.

^[2]^ Gray-Burrows, K.A., et al., Role of patient and public involvement in implementation research: a consensus study. BMJ Qual Saf, 2018. 27(10): p. 858-864.

^[3]^ INVOLVE –National Standards for Public Involvement https://www.invo.org.uk/posttypepublication/national-standards-for-public-involvement/.Accessed 14/01/2020.

^[4]^ INVOLVE – Involvement Cost Calculator https://www.invo.org.uk/resource-centre/payment-and-recognition-for-public-involvement/involvement-cost-calculator/. Accessed 14/01/2020.

#### 63. A standardized framework for risk-based assessment of heterogeneity of treatment effect

##### Alexandros Rekkas^1,2^, Peter R. Rijnbeek^1^, David M. Kent^3^, Ewout W. Steyerberg^2,4^, David van Klaveren^4,3^

###### ^1^Department of Medical Informatics, Erasmus Medical Center, Rotterdam, The Netherlands; ^2^Department of Biomedical Data Sciences, Leiden University Medical Center, Leiden, The Netherlands; ^3^Predictive Analytics and Comparative Effectiveness Center, Tufts Medical Center, Boston, MA, USA; ^4^Department of Public Health, Erasmus Medical Center, Rotterdam, The Netherlands

####### **Correspondence:** Alexandros Rekkas

**Background:** The Observational Health Data Sciences and Informatics (OHDSI) collaborative has established an international network of databases mapped to the Observational Medical Outcomes Partnership (OMOP) Common Data Model ^[1]^, enabling large-scale analyses.

We aimed to develop of a framework for risk-based assessment of heterogeneity of treatment effect (HTE) within the OHDSI setting of analysis of observational data.

**Methods:** The steps required for the standardized analysis are: 1) definition of the problem, i.e. the treatment, the comparator and the outcome(s) of interest; 2) identification of the database(s) in which the framework will be applied; 3) development of the prediction model for the outcome(s) of interest from a propensity score matched sub-population of merged treatment and comparator cohorts, using a large set of standardized predictor variables including demographics, conditions, drugs, measurements procedures and observation concepts; 4) estimation of the propensity scores within strata of predicted risk using large-scale regularized regression, selecting from the same large set of candidate variables; 5) estimation of relative and absolute treatment effects within risk strata—matching or stratification on the propensity score or inverse probability of treatment weighting can be applied.

**Results:** We compared angiotensin-converting enzyme (ACE) inhibitors (treatment) to beta blockers (comparator) with regard to a set of 9 outcomes in patients with hypertension across three observational databases.

**Conclusions:** Reproducible risk-based assessment of HTE in observational data is made possible. (Figure 1) The standardized nature of the process allows its implementation at scale, while the common data model enables collaboration across multiple sites with access to different databases.

**Fig. 1 (abstract 63).**
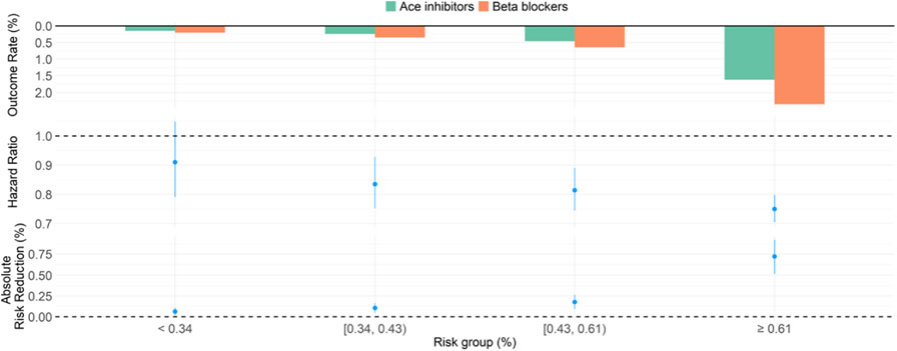
Application in Truven MarketScan Commercial Claims and Encounters (CCAE) database containing enrollees in US employer-sponsored insurance health plans. Patients are divided into quarters of predicted risk of hospitalization with heart failure. Observed event rates by risk quarter are given (top). The hazard ratios—estimated using stratification on the propensity score—show a decreasing trend in favor of ACE inhibitors (middle). The benefits of ACE inhibitors increase strongly at the absolute scale with increasing hospitalization risk (bottom, absolute risk reduction increases from 0.06% to 0.72%).

**Keywords:** heterogeneity of treatment effect, prediction, observational data, framework, electronic health records

**References**

^[1]^ Overhage JM, Ryan PB, Reich CG, Hartzema AG, Stang PE. Validation of a common data model for active safety surveillance research. J Am Med Inform Assoc 2012;19(1):54–60.

#### 64. A prognostic model for overall survival in sporadic Creutzfeldt-Jakob disease

##### Nicole Rübsamen^1^, Franc Llorens^2,3,4^, Peter Hermann^4^, Matthias Schmitz^4^, Anna Villar-Piqué^2,3,4^, Stefan Goebel^4^, André Karch^1^, Inga Zerr^4,5^

###### ^1^Institute for Epidemiology and Social Medicine, University of Münster, Domagkstraße 3, 48149 Münster, Germany; ^2^Network Center for Biomedical Research in Neurodegenerative Diseases (CIBERNED), Institute Carlos III, Campus Bellvitge, Feixa LLarga s/n, 08907 L’Hospitalet de Llobregat, Barcelona, Spain; ^3^Bellvitge Biomedical Research Institute (IDIBELL), Avinguda de la Granvia de l’Hospitalet, 199, 08908 L’Hospitalet de Llobregat, Barcelona, Spain; ^4^Department of Neurology, University Medical School, Robert-Koch-Straße 40, 37075 Göttingen, Germany; ^5^German Center for Neurodegenerative Diseases (DZNE), Von-Siebold-Straße 3A, 37075 Göttingen, Germany

####### **Correspondence:** Nicole Rübsamen

**Background:** Sporadic Creutzfeldt-Jakob disease (sCJD) is the world's most common invariably fatal human prion disease with an incidence rate of 1–2 cases per million and year. Disease duration averages 5–6 months from diagnosis to death, but ranges from weeks to several years.

We aimed to develop an individual prognostic prediction model based on cerebrospinal fluid (CSF) biomarkers and other proposed disease survival modifiers, which are easily obtainable in routine settings at the time of diagnosis.

**Methods:** Probable or definite sCJD cases from a German surveillance study (1993–2017) were included. The prognostic accuracy to predict overall survival after sCJD diagnosis was measured by the *c* statistic of a model derived from a multivariable Cox proportional hazard regression.

**Results:** Complete information about age, sex, codon 129 genotype, presence of 14-3-3 in the CSF, and CSF tau concentrations was available for 1,226 out of 2,908 sCJD cases. The median age at diagnosis was 66 years (range 19–89 years). The male-to-female ratio was 1:1. A Cox proportional hazard model containing age, sex, genotype, CSF tau and the interaction terms age × tau, sex × tau, and sex × genotype was selected as the model with the highest c statistic (0.686, 95% CI 0.665–0.707) using cross-validation. This model was well calibrated. A score chart was derived to predict 6-month survival and median survival time (Figure 1).

**Conclusions:** We developed an individual prediction model with moderate to good accuracy. The score chart developed in this study serves as a hands-on prediction tool for clinical practice, allowing better planning of care after sCJD diagnosis and easier identification of potential participants for future treatment trials.

**Fig. 1 (abstract 64).**
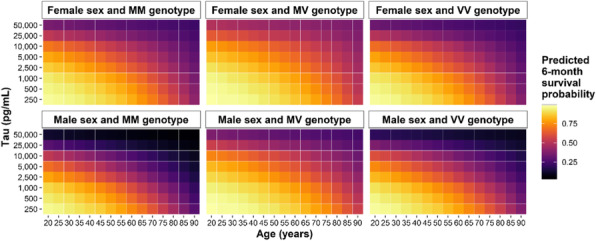
Score chart for predicting 6-month survival probability of sCJD patients

**Keywords:** Cerebrospinal fluid, neurodegeneration, prognosis, sporadic Creutzfeldt-Jakob disease, tau

#### 65. Model-based ROC (mROC) Curve: A Method For Assessing The Effect Of Case-mix And Model Calibration on The ROC Curve

##### Mohsen Sadatsafavi^1^, Paramita Saha- Chaudhuri^2^, John Petkau^3^

###### ^1^Faculty of Pharmaceutical Sciences, The University of British Columbia. 2405 Wesbrook Mall, Vancouver, BC, V6T1Z3, Canada; ^2^Department of Epidemiology, Biostatistics & Occupational Health, McGill University. Purvis Hall, 1020 Pine Avenue West, Montreal, QC, Canada; ^3^Department of Statistics, 2207 Main Mall, Vancouver, BC, Canada

####### **Correspondence:** Mohsen Sadatsafavi

**Background:** The performance of risk prediction models is often characterized in terms of discrimination and calibration. The Receiver Operating Characteristic (ROC) curve is widely used for evaluating model discrimination. When comparing ROC curves between development and validation samples, the effect of case-mix makes the interpretation of discrepancies difficult. Further, compared to model discrimination, evaluating model calibration has not received the same level of attention in medical literature. The most commonly used graphical method for model calibration, the calibration plot, requires specification of smoothing parameters or number of groups.

**Methods:** This abstract introduces the ‘model-based’ ROC (mROC) curve, the ROC curve that should be observed if the prediction model is calibrated in the external population. Unlike the ROC curve, the mROC curve is affected by even monotonical transformations of predicted risks, thus is sensitive to model calibration. We show that moderate calibration (actual risk being p% among those with predicted risk of p%) is a sufficient condition for the convergence of mROC and ROC curves. Accordingly, we propose a novel test statistic for calibration that does not require any arbitrary parameterization.

**Results:** Through an example, we demonstrate how mROC separates the effect of case-mix and model mis-calibration when comparing ROC curves from different samples (Figure 1). We present the results of simulation studies that confirm the properties of the new calibration test. A case study puts the developments in a practical context.

**Conclusion:** mROC can easily be constructed and used to interpret the effect of case-mix and calibration on the ROC plot. This can facilitate interpretation of the ROC curve in external validation studies. Given the popularity of ROC curves among applied investigators, this framework can further promote assessment of model calibration.

**Fig. 1 (abstract 65).**
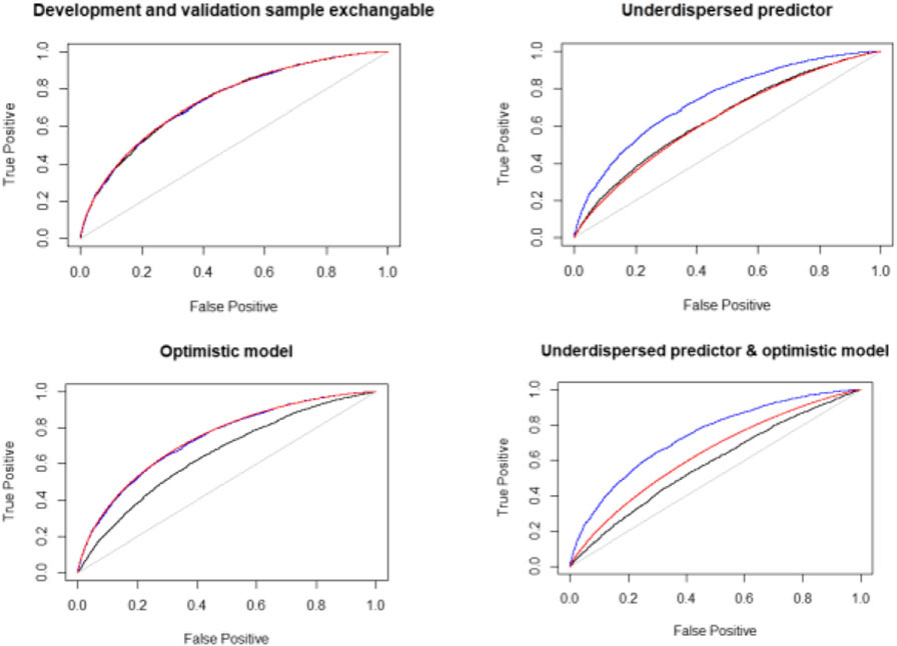
Empirical ROC in the development (blue) and validation (black) samples, and mROC (red) curves for an exemplary study.

**Keywords:** Clinical prediction, risk prediction, receiver operating characteristic curve

#### 66. A Permutation Test Approach to Provide Exact Inference for Incremental Gain from Nested Regression Models

##### P. Saha-Chaudhuri^1^, L.C. Cheung^2^, H. A. Katki^2^

###### ^1^Department of Epidemiology, Biostatistics and Occupational Health, McGill University, Montreal, Canada; ^2^Division of Cancer Epidemiology and Genetics, National Cancer Institute, NIH, Rockville, MD, USA

###### **Correspondence:** P. Saha-Chaudhuri

**Background:** Assessment of the incremental gain and impact of a novel marker to better predict disease risk is an ongoing quest in many clinical disciplines. For binary and time-to-event outcomes, two popular metrics used to assess incremental gain are difference in the C-index or the Area under the ROC curve (dAUC) and Integrated Discrimination Improvement (IDI). However, inference for these two measures are complex for their non-standard distributions, especially, while comparing nested models that are build and evaluated on the same dataset.

**Methods and results**: We propose an easy-to-implement permutation test for dAUC and IDI to provide exact inference for the incremental gain. Via extensive simulation studies, we show that for small to moderate sample sizes, the type I error rate and power for dAUC and IDI are comparable the type I error rate and power for the likelihood ratio test and Wald test for comparing nested logistic and Cox proportional hazards models. In addition, we also assess the performance of the permutation test for classification trees. We demonstrate the approach in a real dataset where the incremental value of time-to-first cigarette to select ever-smokers for lung cancer was assessed.

**Conclusions:** We show that permutation test can be used effectively for assessing incremental value of a marker based on nested models. Our work provides an viable solution for assessment of incremental gain for many scientific and clinical scenarios.

**Keywords:** Incremental value, AUC, IDI, permutation test, logistic regression, Cox proportional hazards model

#### 67. Probabilistic data standardisation of big heterogeneous datasets in biomedicine

##### Alexia Sampri, Nophar Geifman, Philip Couch, and Niels Peek

###### Division of Informatics, Imaging and Data Sciences University of Manchester, Manchester, UK

####### **Correspondence:** Alexia Sampri

**Background:** Putting data together from different sources into a homogeneous data resource would enable unprecedented opportunities to study human health. However, these disparate collections of data are inevitably heterogeneous and have made aggregation a difficult challenge. We focus on the issue of content heterogeneity in data integration. Traditional approaches for resolving content heterogeneity map all source datasets to a common data model that includes only shared data items.

Our focus is on integration of structured data. We assume that each one of these datasets that needed to be integrated consists of a single table; and that each of these datasets describes a disjoint set of entities. Therefore, record linkage is not needed.

**Methods:** We propose the development of improved, probabilistic approaches for data integration, capable of advancing the timely utilisation of large-scale biomedical data resources. Our approaches aim to forego the need for perfect data standardisation by employing a probabilistic post-alignment of data items that is integrated with statistical inference. Using these approaches, missing or semantically ambiguous information is estimated from datasets potentially relevant for answering the research question.

**Results:** The MAximizing Sle ThERapeutic PotentiaL by Application of Novel Stratified approaches programme (MASTERPLANS) aims to improve care for Systemic Lupus Erythematosus patients by taking a precision medicine approach to identifying groups of patients that respond to biologic therapies. Based on dataset examples provided by MASTERPLANS we describe and evaluate the proposed probabilistic data integration approaches.

**Conclusions:** Our approaches insist on the future existence of health data heterogeneity. They strive for post alignment of Big datasets. As a post-alignment of heterogeneous data sources will be always imperfect and it is not a problem if we estimate the probability that they are. Our approaches are also pragmatic because they always provide an answer.

**Keywords:** Big Data, probabilistic data integration, data heterogeneity

#### 68. Predicting Biomarker success: a new toolkit

##### Katerina-Vanessa Savva^1^, Melody Ni^1^, Bhamini V. Vadhwana^1^, Sara H. Jamel^1^, Elsa Angelini^2^, George Hanna^1^, and Christopher J. Peters^1^

###### ^1^Department of Surgery and Cancer, Imperial College London, UK; ^2^Institute of Translational Medicine and Therapeutics (ITMAT), Imperial College London, UK

####### **Correspondence:** Katerina-Vanessa Savva

**Background:** Increased resources have been spent on cancer biomarker (BM) discovery, for both prognostic and diagnostic purposes, but very few of these BMs have been clinically adopted. Therefore, in an attempt to bridge the gap between BM discovery and clinical use, this study aims to generate a BM assesment toolkit based on literature-reported attributes associated with successful BM implementation.

**Methods:** A checklist of BM attributes was created using Medline and Embase databases according to PRISMA guidelines. Retrospective validation of the checklist was achieved by six independent systematic literature searches using keywords/subheadings related to successfully implemented (n=2) and stalled (n=5) breast cancer BMs. Composite aggregated scores were generated for each selected publication based on the presence/absence of a characteristic listed in the BM checklist. Subsequently, logistic regression was performed to assess the relationship of each BM attribute/total average scores and the clinical implementation status.

**Results:** Attributes retrieved from literature and guidelines (n=125) were included in the BM toolkit. Average total % scores generated based on these attributes were significantly higher in the successfully implemented BM group (P<0.0001). The logistic regression model between the average total % score and BM clinical implementation status reached significance with sensitivity and specificity of 97.5% and 95.4%, respectively.

**Conclusions:** This study generated a validated checklist with literature-reported attributes linked with successful BM implementation. Upon future work and prospective validation, this toolkit could be used i) to detect BMs with the highest potential of being clinically implemented and ii) to shape how BM studies are designed and performed.

**Keywords:** Biomarkers, clinical implementation, checklist

#### 69. Assessing the impact of test measurement uncertainty on clinical and health-economic outcomes: a case study

##### Alison F Smith^1,2^, Michael P Messenger^2,3^, Claire T Hulme^4^, Peter S Hall^5^, Bethany Shinkins^1,2^

###### ^1^Test Evaluation Group, Academic Unit of Health Economics, University of Leeds, Leeds, UK; ^2^NIHR Leeds In Vitro Diagnostic (IVD) Co-operative, Leeds, UK; ^3^Leeds Centre for Personalised Medicine and Health, University of Leeds, Leeds, UK; ^4^Health Economics Group, University of Exeter, Exeter, UK; ^5^Cancer Research UK Edinburgh Centre, MRC Institute of Genetics and Molecular Medicine, University of Edinburgh, Edinburgh, UK

####### **Correspondence:** Alison F Smith

**Background:** Many factors can introduce bias and imprecision (i.e. measurement uncertainty) into in-vitro test measurements. If, as a result, test values are incorrectly observed as lying outside of key decision thresholds, then this uncertainty can affect clinical and health-economic outcomes. Currently, however, this impact is rarely considered within laboratory or test evaluation studies.

We aimed to illustrate methods for assessing the impact of test measurement uncertainty on outcomes.

**Methods:** In a recent review, we identified methods for assessing the impact of measurement uncertainty on test outcomes. In this study, we applied the *error model simulation approach* (based on iterative application of bias and imprecision) to a case study test: faecal calprotectin (FC) for the diagnosis of Inflammatory Bowel Disease (IBD). Two primary care FC pathways were evaluated: the 'NICE FC pathway' (single FC test; 50 μg/g diagnostic cut-off threshold) and the 'York FC Care Pathway (YFCCP)' (a repeat-FC strategy; 100 μg/g threshold). The error model simulation was embedded within an existing decision analytic model, to evaluate the impact of measurement uncertainty on diagnostic accuracy, clinical-utility and cost-effectiveness outcomes.

**Results:** The NICE FC pathway was found to be highly volatile to positive bias. The YFCCP meanwhile was largely robust to increased measurement uncertainty, suggesting that this pathway is suitable for wide-scale adoption. Using the simulated results, acceptable regions of analytical performance (i.e. maximum bounds for bias and imprecision) were identified, based on the impact of measurement uncertainty on clinical and health-economic outcomes.

**Conclusions:** The error model approach provides a useful method for assessing the impact of measurement uncertainty on outcomes. This information is important both for clinical decision makers (to inform whether or not to adopt a new test) and laboratory professionals (to inform evidence-based implementation and monitoring practices for tests within the laboratory).

**Keywords:** Measurement uncertainty, simulation, test evaluation

#### 70. Beyond the laboratory: methods to assess the impact of test measurement uncertainty on outcomes

##### Alison F Smith^1,2^, Michael P Messenger^2,3^, Claire T Hulme^4^, Peter S Hall^5^, Bethany Shinkins^1,2^

###### ^1^Test Evaluation Group, Academic Unit of Health Economics, University of Leeds, Leeds, UK; ^2^NIHR Leeds In Vitro Diagnostic (IVD) Co-operative, Leeds, UK; ^3^Leeds Centre for Personalised Medicine and Health, University of Leeds, Leeds, UK; ^4^Health Economics Group, University of Exeter, Exeter, UK; ^5^Cancer Research UK Edinburgh Centre, MRC Institute of Genetics and Molecular Medicine, University of Edinburgh, Edinburgh, UK

####### **Correspondence:** Alison F Smith

**Background:** For medical tests that have a central role in clinical decision-making, international laboratory guidelines advocate *outcome-based* analytical performance specifications (APS) – i.e. measurement performance goals derived from the expected impact of measurement uncertainty on clinical outcomes. The identification of outcome-based APS relies on indirect studies (e.g. simulation) to assess the impact of test measurement uncertainty on outcomes. Currently however, there is limited awareness of available methods in this context. Increased awareness and understanding of indirect study methods could further inform test evaluation methodologies.

We aimed to identify indirect methods for assessing the impact of measurement uncertainty (i.e. bias and imprecision) on outcomes (clinical performance, clinical utility and/or costs).

**Methods**: A methodology review consisting of database searches and extensive citation tracking was conducted to identify studies using indirect methods to incorporate or evaluate the impact of test measurement uncertainty on outcomes.

**Results**: Eighty-two studies were identified, most of which evaluated the impact of imprecision and/or bias on clinical accuracy. A common three-step analytical framework underpinning the various methods was apparent: (1) estimation of “true” test values; (2) estimation of measured test values (incorporating uncertainty); and (3) estimation of the impact of discrepancies between (1) and (2) on specified outcomes. Simulation techniques have become a common approach over the past two decades; the most flexible method – the *error model simulation approach* – is based on the iterative application of bias and imprecision onto baseline “true” values. Whilst previous studies have focused on clinical performance (e.g. diagnostic accuracy), evaluations can be feasibly extended to clinical-utility and cost-effectiveness outcomes using decision analytic models.

**Conclusions**: Various approaches are available for conducting indirect assessments to inform outcome-based APS and test evaluations. This study provides a useful overview of methods and key considerations for future research.

**Keywords:** Measurement uncertainty, methodology review, analytical performance specifications, test evaluation

#### 71. Impact of prediction models in obstetric care: the Expect study

##### Pim van Montfort^1^, Hubertina Scheepers^2^, Carmen Dirksen^3^, Ivo van Dooren^4^, Linda Meertens^1^, Sander van Kuijk^3^, Ella Wijnen^5^, Maartje Zelis^6^, Iris M. Zwaan^7^, Marc Spaanderman^2^, Luc Smits^1^

###### ^1^Department of Epidemiology, Care and Public Health Research Institute (CAPHRI), Maastricht University, Maastricht, The Netherlands; ^2^Department of Obstetrics and Gynaecology, School for Oncology and Developmental Biology (GROW), Maastricht University Medical Centre, Maastricht, The Netherlands; ^3^Department of Clinical Epidemiology and Medical Technology Assessment (KEMTA), Care and Public Health Research Institute (CAPHRI), Maastricht University Medical Centre, Maastricht, The Netherlands; ^4^Department of Obstetrics and Gynaecology, Sint Jans Gasthuis Weert, Weert, The Netherlands; ^5^Department of Obstetrics and Gynaecology, VieCuri Medical Centre, Venlo, The Netherlands; ^6^Department of Obstetrics and Gynaecology, Zuyderland Medical Centre, Heerlen, The Netherlands; ^7^Department of Obstetrics and Gynaecology, Laurentius Hospital, Roermond, The Netherlands

####### **Correspondence:** Luc Smits

**Background:** Obstetric healthcare relies on an adequate antepartum risk selection. Most guidelines used for risk stratification, however, do not assess absolute risks. In 2017, a prediction tool was implemented in a Dutch region. This tool combines first trimester prediction models with obstetric care paths tailored to the individual risk profile, enabling risk-based care (RBC).

We aimed to assess impact and cost-effectiveness of RBC compared to care-as-usual (CAU) in a general population.

**Methods:** A before-after study was conducted using two multicenter prospective cohorts. The first cohort (2013-2015) received CAU, the second cohort (2017-2018) received RBC. Health outcomes were 1) a composite of adverse perinatal outcomes and 2) maternal quality adjusted life years (QALYs). Costs were estimated using a healthcare perspective from conception to six weeks after the due date. Mean costs per woman, cost differences between the two groups, as well as incremental cost effectiveness ratios were calculated. Sensitivity analyses were performed to evaluate the robustness of the findings.

**Fig. 1 (abstract 71).**
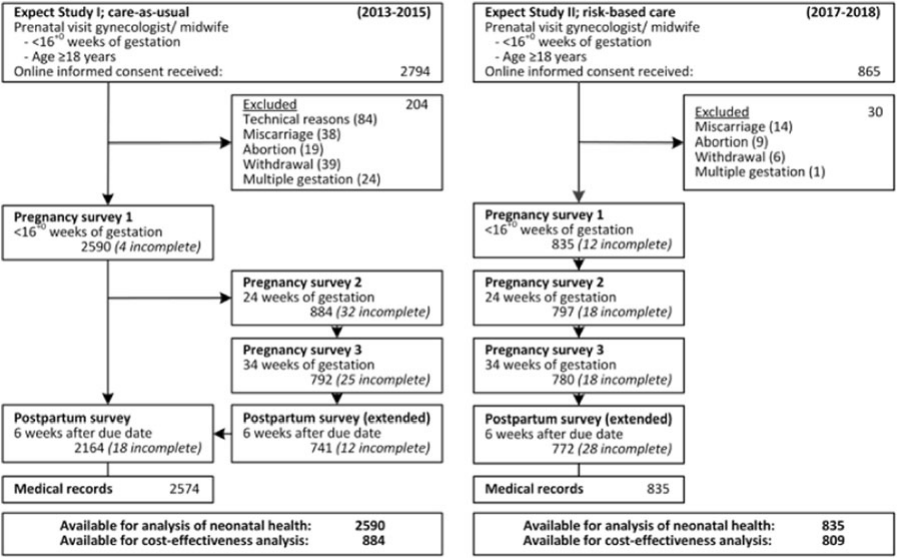
flowchart of included women

**Results:** In total 3,425 women were included. (Figure 1) In nulliparous women there was a significant reduction of perinatal adverse outcomes among the RBC group (aOR 0.56; 95%CI 0.32-0.94), but not in multiparous women. Mean costs per pregnant woman were significantly lower for RBC (mean difference -€2,766, 95%CI -€3,700 – -€1,825). No differences in maternal quality of life, adjusted for baseline health, were observed.

**Conclusion:** In the Netherlands, RBC in nulliparous women was associated with improved perinatal outcomes as compared to CAU. Furthermore, RBC was cost-effective compared to CAU and resulted in lower healthcare costs.

**Keywords:** Impact study, prediction models, obstetrics, risk-based care, perinatal outcomes, cost-effectiveness, Expect study

#### 72. Development and validation of prediction models for pre-eclampsia: An Individual Participant Data Meta-analysis

##### John Allotey^1^, Kym Snell^2^, Richard Riley^2^, Shakila Thangaratinam^1,3^ for the IPPIC Collaborative Network*

###### ^1^Barts Research Centre for Women’s Health (BARC), Barts and the London School of Medicine and Dentistry, Queen Mary University of London, London, UK; ^2^Centre for Prognosis Research, School of Primary, Community and Social Care, Keele University, Keele, UK; ^3^Institute of Metabolism and Systems Research, University of Birmingham, Birmingham, UK

####### **Correspondence:** Kym Snell

**Background:** Pre-eclampsia is the most predicted obstetric outcome, with more than 130 prognostic models developed.^[1]^ A quarter of these have been externally validated, and showed only modest predictive performance, characterised by methodological shortcomings in development including overfitting of models, small event numbers in development datasets and predictors not varied enough to adequately capture the differences between women. Access to IPD from multiple studies will provide increased sample size with more outcomes to evaluate several candidate prognostic factors, beyond what would have been possible in a single study, to subsequently develop clinically relevant and robust models. It will also enable the evaluation of any prediction model developed across different settings and population case-mix.

We aimed to develop and validate pre-eclampsia prediction models using IPD from multiple studies.

**Methods:** Logistic regression with a random intercept to account for clustering by study for model development. Internal-external cross-validation using random-effects meta-analysis to summarise performance measures across studies.

**Results:** The International Prediction of Pregnancy Complications (IPPIC) network^[2]^ is a group of 125 researchers contributing data of 3,674,684 pregnancies from 78 datasets. Twelve prediction models were developed, four each for any, early and late-onset pre-eclampsia. 3-11 datasets were used to develop each model depending on the availability of predictors within datasets. Average models discrimination were good (0.68-0.83), however calibration performance was heterogeneous across datasets. The models showed the highest net-benefit for predicted probability thresholds in nulliparous women at thresholds above 5%.

**Conclusions:** The IPPIC models on average showed promising predictive performance. However before application in practice, recalibration of model parameters to particular populations and settings may be needed. Additional predictors may improve the predictive performance of the models.

**Keywords:** Pre-eclampsia, prediction model, individual participant data

**References**

^[1]^ Allotey J, et al. Accuracy of clinical characteristics … using IPD meta-analysis. *HTA 2020 (in press)*

^[2]^ Allotey J, et al. External validation, update...the IPPIC pre-eclampsia Network protocol. *DAPR* 2017;1:16.

#### 73. External validation of prognostic models to predict pre-eclampsia: An Individual Participant Data Meta-analysis

##### John Allotey^1^, Kym Snell^2^, Richard Riley^2^, Shakila Thangaratinam^1,3^ for the IPPIC Collaborative Network*

###### ^1^Barts Research Centre for Women’s Health (BARC), Barts and the London School of Medicine and Dentistry, Queen Mary University of London, London, UK; ^2^Centre for Prognosis Research, School of Primary, Community and Social Care, Keele University, Keele, UK; ^3^Institute of Metabolism and Systems Research, University of Birmingham, Birmingham, UK

####### **Correspondence:** Kym Snell

**Background:** With about 70 published prognostic models, pre-eclampsia is the most frequently predicted outcome in obstetrics, yet only 10% have been externally validated,^1^ and none are recommended in national guidelines for routine clinical use, partly due to a paucity in external validation. Access to individual participant data (IPD) from multiple studies allows for external validation in different populations. It saves cost by reusing existing data thereby reducing research waste, and increases the sample-size with more outcomes beyond what would have been possible in a single study, allowing for evaluation of prediction models of rare conditions, such as early-onset pre-eclampsia, which affects only 0.5% of all pregnancies.

We aimed to assess the external predictive performance of existing prognostic models for pre-eclampsia within the UK healthcare setting.

**Methods:** Systematic review and external validation of prognostic models using IPD meta-analysis. Performance was evaluated using measures of discrimination, calibration and net-benefit. Random-effects meta-analysis was used to summarise and estimate heterogeneity in model performance across studies.

**Results:** IPD from 11 UK cohort studies (217,415 pregnant women) within the International Prediction of Pregnancy Complications network^[2]^ were used for external validation. Medline and Pubmed searches up to December 2017 identified 71 articles, describing the development of 131 prognostic models for predicting pre-eclampsia. Half (51%, 67/131) provided the full model equations required for validation, but only a third (36%, 24/67) could be validated because all predictors in the model were recorded in at least one study of the IPD. Summary C-statistics were modest (0.6-0.7) and calibration was generally poor (<1) suggesting overfitting.

**Conclusions:** Evidence is limited to support the implementation of evaluated models in clinical practice. Findings suggests methodological failings in their development.

**Keywords:** Pre-eclampsia, external validation, prediction model, individual participant data

**References**

^**[**1]^Kleinrouweler CE, et al. Prognostic models in obstetrics. *AJOG* 2015.

^[2]^Allotey J, et al. External validation, update and development...the IPPIC pre-eclampsia Network protocol. *DAPR* 2017;1:16.

#### 74. Simulation-based sample size calculations for studies externally validating a prediction model

##### Kym IE Snell^1^, Lucinda Archer^1^, Joie Ensor^1^, Laura J Bonnett^2^, Bob Phillips^3*,*^ Gary S Collins^4^, Richard D Riley^1^

###### ^1^Centre for Prognosis Research, School of Primary, Community and Social Care, Keele University, Keele, Staffordshire, UK; ^2^Department of Biostatistics, University of Liverpool, Liverpool, UK; ^3^Centre for Reviews and Dissemination, University of York, York, UK; ^4^Centre for Statistics in Medicine, University of Oxford, Oxford, UK

####### **Correspondence:** Kym IE Snell

**Background:** Sample size requirements for external validation of a prediction model are often based on ‘rules-of-thumb’ such as requiring at least 100 (or even 200) events or non-events. Although often overlooked, it is not simply the point estimates of performance measures that are of interest but also the precision in these estimates. Researchers should therefore ensure that validation studies are large enough to estimate performance measures with reasonable precision.

We aimed to investigate factors affecting precision of performance measures, and demonstrate a simulation-based approach for determining appropriate sample sizes for external validation studies.

**Methods:** We conducted a simulation study to investigate the relationship between various factors (outcome prevalence, linear predictor distribution (LPSD), total sample size) and precision of performance measures for a logistic regression model, and developed a simulation-based approach to determine the minimum sample size required to achieve sufficiently narrow confidence intervals for all predictive performance measures of interest.

**Results**: The simulation study demonstrates that factors other than number of events affect precision of performance measures (including LPSD and total sample size) and that even with 100 or 200 events and non-events, 95% CIs remain wide in some settings. By specifying the desired precision of performance measures and distribution of the linear predictor (e.g. based on development data), our simulation-based approach can be used to tailor sample size calculations. The approach will be illustrated for designing a validation study for a diagnostic model for deep vein thrombosis, based on published data.

**Conclusions**: Sample size for validation of logistic models cannot be solved easily using closed form solutions and rules-of-thumb are often too simplistic and fail in individual settings. In situations where the distribution of the linear predictor can be ascertained, a simulation-based approach allows the sample size to be tailored to the setting.

**Keywords:** Sample size, validation, prediction, simulation

#### 75. TRIPOD-SR: An extension to reporting guidelines for systematic reviews of prediction model studies

##### Kym IE Snell^1^, Brooke Levis^1^, Thomas PA Debray^2^, Lotty Hooft^2^, Paula Dhiman^3^, Johannes B Reitsma^2^, Karel GM Moons^2^, Gary S Collins^3^, Richard D Riley^1^

###### ^1^ Centre for Prognosis Research, School of Primary, Community and Social Care, Keele University, Keele, Staffordshire, UK; ^2^Julius Center for Health Sciences and Primary Care, University Medical Center Utrecht, Utrecht University, Utrecht, The Netherlands; ^3^Centre for Statistics in Medicine, University of Oxford, Oxford, UK

####### **Correspondence:** Kym IE Snell

**Background:** Guidelines exist for reporting the development and validation of prediction models (TRIPOD), and for reporting systematic reviews (PRISMA). However, no specific guidance exists for reporting systematic reviews of prediction models which can have different aims, ranging from identifying models through to comparing predictive performance of models. Therefore, existing reporting guidelines require modification to be more suitable for systematic reviews of prediction model studies.

We aimed to develop an extension to TRIPOD, specific to systematic reviews of prediction model studies.

**Methods:** Existing reporting guidelines were reviewed. Relevant guideline items were combined and assessed for suitability by two researchers, considering the different aims of systematic reviews: i) identification of prediction models within a broad clinical field, ii) identification of prediction models for a target population, iii) identification of models for a particular outcome, iv) assessing the performance of a particular model, and v) comparison of models (in terms of predictive performance). Item suitability and wording were discussed within the working group and a draft extension to TRIPOD was produced. An online Delphi survey was conducted, using researchers with experience in systematic review and prediction modelling to provide feedback on the proposed items.

**Results:** PRISMA and TRIPOD-Cluster (in development) were identified as the most relevant reporting guidelines. They contained many overlapping items; while PRISMA contained some items specific to systematic reviews, TRIPOD-IPD contained some items specific to prediction models. Items from both guidelines were combined, resulting in many items being merged and modified, while other items specific to model development or individual participant data were removed. Feedback from the Delphi survey was incorporated and the draft extension will be presented, welcoming feedback before a second Delphi survey.

**Conclusions:** TRIPOD-SR is an extension of existing reporting guidelines that is being developed to provide more tailored guidance for reporting systematic reviews of prediction models.

**Keywords:** Reporting guidelines, systematic reviews, prediction models

#### 76. Use and misuse of the calibration slope

##### Richard Stevens^1^, Katrina Poppe^2^

###### ^1^Nuffield Dept Primary Care Health Sciences, University of Oxford, Woodstock Road, Oxford, United Kingdom; ^2^Faculty of Medical and Health Sciences, University of Auckland, Auckland, New Zealand

####### **Correspondence:** Richard Stevens

**Background:** The slope of a calibration plot is often referred to as “calibration slope”. Methodology texts emphasize the slope should not be used in isolation but accompanied by other metrics and graphs: poor calibration, by any definition, can occur even when the slope is perfect (equals 1).

**Method:** We review recent usage of the calibration slope.

**Results:** In 33 validation papers (24 external) published 2017-2018, 25 papers identified the slope with calibration, 1 identified calibration slope with discrimination and 7 used the term calibration slope without explicitly interpreting it. In 17 papers (52%) the slope was used as sole measure of calibration. We are currently reviewing papers from 2019 and 2020.

**Conclusions:** The paper often cited as the origin of the “calibration slope” did not use the term calibration, but “spread”. More recently “spread” has been identified in some papers as an aspect of calibration and in others as an aspect of discrimination, sometimes by the same authors. We resolve this apparent paradox by proposing that calibration and discrimination are not a dichotomy. If we equate the A (calibration-in-the-large), B (calibration slope) and C (discrimination) of Steyerberg and Vergouwe’s ABCD[1] with bias, spread and ordering, then we can see that good calibration-in-the-large equates to low bias; calibration as often defined equates to low bias and adequate spread; good discrimination requires correct spread and correct ordering; and moderate to strong calibration, as defined by Van Calster[2], requires low bias, adequate spread and correct ordering. Authors, reviewers and editors have a duty to discourage the perception that calibration is a unidimensional construct quantifiable by a single statistic, the slope.

**Keywords:** Validation, calibration, discrimination, spread, slope

**References**

^[1]^ Steyerberg & Vergouwe, European Heart Journal (2014) 35, 1925–1931

^[2]^ Van Calster et al. Journal of Clinical Epidemiology 74 (2016) 167e176

#### 77. Statistical methods for estimating sources of variability in count biomarkers

##### Kostas Tryposkiadis^1,2^, Alice Sitch^1,2^, Malcolm Price^1,2^, Jon Deeks^1,2^

###### ^1^Test Evaluation Research Group, Institute of Applied Health Research, University of Birmingham, Birmingham, UK; ^2^NIHR Birmingham Biomedical Research Centre, University Hospitals Birmingham NHS Foundation Trust and University of Birmingham, Birmingham, UK

####### **Correspondence:** Kostas Tryposkiadis

**Background:** Analysis using random effects linear models is the established method used in biological variability studies to attribute the observed variability arising from between-patient differences, within-patient differences, and measurement error. However, these models assume underlying normality, and thus may not be applicable for biomarkers based on counts.

We aimed to present methods for estimating sources of variability in count-based biomarkers and apply and compare approaches in a case study of patients with Sjogren’s syndrome.

**Methods:** Both Poisson and negative binomial models are appropriate for analysis of count data, and methods for obtaining between and within-patient variance estimates are described in Leckie et al^[1]^. We analysed the biomarker data using random effects Poisson and negative binomial models, and for comparison, using a random effects linear regression model. The intra-class-correlation (ICC) was calculated as a ratio of the between-patient variance over the total variance, and was compared across the different models. The AIC and BIC criteria were used to assess each model’s performance. Data from 32 patients with Sjogren’s syndrome was used as a case study, considering the focus score, calculated for each salivary gland observed in each biopsy as the number of foci over the glandular area, multiplied by 4. Between-patient and within-patient-between-gland sources of variability were estimated.

**Results:** The ICC estimates obtained from Poisson (0.323) and negative binomial models (0.310) were similar, and higher than the linear regression model (0.222). AIC and BIC values were similar for Poisson (AIC=463.63, BIC=469.84) and negative binomial models (AIC=465.55, BIC=474.87) and indicated both were a better fit than the linear regression model (AIC=632.69, BIC=642.01).

**Conclusions:** It is important to properly model the distribution of biomarkers based on count data to correctly estimate sources of variability and measurement error.

**Keywords:** Biomarkers, variability, count data

**References**

^[1]^Leckie et al, 2019. arXiv: 1911.06888 [stat ME].

#### 78. Statistical methods for the meta-analysis of reliability estimates reported in biological variability studies

##### Kostas Tryposkiadis^1,2^, Jac Dinnes^1,2^, Alice Sitch^1,2^, Malcolm Price^1,2^, Jon Deeks^1,2^

###### ^1^Test Evaluation Research Group, Institute of Applied Health Research, University of Birmingham, Edgbaston, Birmingham B15 2TT, United Kingdom; ^2^NIHR Birmingham Biomedical Research Centre, University Hospitals Birmingham NHS Foundation Trust and University of Birmingham, UK

####### **Correspondence:** Kostas Tryposkiadis

**Background:** Biomarkers and tests are often used to diagnose or monitor a condition, or function as outcomes in clinical trials. Key questions arises on the measurement properties of biomarkers when used for such purposes, as measurements are subject to variability, such as analytical, biological, and intra/inter-rater. Methods for meta-analysis are required in order to synthesize results in systematic reviews from individual studies assessing the reliability of biomarkers or tests.

We aimed to review the current state of methods used for meta-analysis of reliability estimates reported in biological variability studies.

**Methods:** Published systematic reviews reporting the reliability of any test measuring presence or progress of any pathological condition were identified by searches of Medline and Embase from 2010-19. Detailed information was extracted regarding: the experimental test; the condition; the review methodology including the literature search, approach to quality assessment, the statistical methodology used to examine reliability; and the results each study reported.

**Results:** 228 reviews were identified, with only 23 performing a meta-analysis of the reported estimates. The most common meta-analytical estimate was the intra class correlation (61%), with 3 studies using the Fisher’s Z transformation to account for the non-normal distribution of ICC data. Other reported statistics include the Kappa coefficient, standard error of measurement, coefficient of variation, limits of agreement, repeatability coefficients, linear regression based R^2^, and correlation coefficients. The majority of studies (78%) constructed forest plots and used random effects models to account for differences between studies. One study used a fixed effects model, while the method was not specified in 2 studies. Other approaches include pooling the data and performing linear regression, Bland-Altman analysis on the test-retest values, and describing the distribution of the study results.

**Conclusions:** Any limitations in the statistical estimates and meta-analysis methods used to date will be explored and presented.

**Keywords:** Biomarker, reliability, meta-analysis

#### 79. Pre-analytical error for three point of care venous blood testing platforms in acute ambulatory care

##### Thomas R. Fanshawe^1^, Margaret Glogowska^1^, George Edwards^1^, Philip J. Turner^1^, Ian Smith^2^, Rosie Steele^2^, Caroline Croxson^1^, Jordan S. T. Bowen^2^, Gail N. Hayward^1^

###### ^1^Nuffield Department of Primary Care Health Sciences, University of Oxford, Oxford, UK; ^2^Oxford University Hospitals NHS Foundation Trust, Oxford, UK

####### **Correspondence:** Philip J. Turner

**Background:** Point of care blood testing to aid diagnosis is becoming increasingly common in acute ambulatory settings and enables timely investigation of a range of diagnostic markers. However, this testing allows scope for errors in the pre-analytical phase, which depends on the operator handling and transferring specimens correctly. The extent and nature of these pre-analytical errors in clinical settings has not been widely reported.

**Methods:** We carried out a convergent parallel mixed-methods service evaluation to investigate pre-analytical errors leading to a machine error reports in a large acute hospital trust in the UK. The quantitative component comprised a retrospective analysis of all recorded error codes from Abbott Point of Care i-STAT 1, i-STAT Alinity and Abbott Rapid Diagnostics Afinion devices to summarise the error frequencies and reasons for error, focusing on those attributable to the operator. The qualitative component included a prospective ethnographic study and a secondary analysis of an existing ethnographic dataset, based in hospital-based ambulatory care and community ambulatory care respectively.

**Results:** The i-STAT had the highest usage (113,266 tests, January 2016-December 2018). As a percentage of all tests attempted, its device-recorded overall error rate was 6.8% (95% confidence interval 6.6% to 6.9%), and in the period when reliable data could be obtained, the operator-attributable error rate was 2.3% (2.2% to 2.4%). Staff identified that the most difficult step was the filling of cartridges, but that this could be improved through practice, with a perception that cartridge wastage through errors was rare.

**Conclusions:** In the observed settings, the rate of errors attributable to operators of the primary point of care device was less than 1 in 40. In some cases, errors may lead to a small increase in resource use or time required so adequate staff training is necessary to prevent adverse impact on patient care.

**Keywords**: Point of care, ambulatory Care, pre-analytical error

#### 80. Development of a model to predict the likelihood of a genetic variant causing familial hypercholesterolaemia

##### Rachel O'Leary^1,2^, Samuel G. Urwin^2,3^, Clare Lendrem^2,3^, Ahai Luvai^4^, R. Dermot G. Neely^2^, A. Joy Allen^2^

###### ^1^Northern Medical Physics and Clinical Engineering, The Newcastle-upon-Tyne Hospitals NHS Foundation Trust, Newcastle-upon-Tyne, UK; ^2^The National Institute for Health Research Newcastle In Vitro Diagnostics Co-operative, Newcastle-upon-Tyne, UK; ^3^Translational and Clinical Research Institute, Faculty of Medical Sciences, Newcastle University, Newcastle-upon-Tyne, UK; ^4^Laboratory Medicine, The Newcastle-upon-Tyne Hospitals NHS Foundation Trust, Newcastle-upon-Tyne, UK

####### **Correspondence:** Samuel G. Urwin

**Background:** Familial hypercholesterolaemia (FH) is a common, life-threatening genetic condition associated with long-term elevation of cholesterol levels in the blood. A diagnosis of FH can be confirmed by genetic testing; however, it is expensive, and can often be mistargeted due to limitations of the scoring systems used to refer patients.

We aimed to develop a model using clinical data to improve the targeting of genetic testing by predicting the likelihood of a patient having a variant causing FH.

**Methods:** Data were obtained from 243 patients referred for genetic testing on suspicion of having FH. Forward stepwise logistic regression was performed, with variant status (binary) as the dependent variable, and age, sex, individual components of the Dutch Lipid Clinic Network (DLCN) criteria, total cholesterol (TC), high-density lipoprotein cholesterol (HDL-C), triglycerides, and low-density lipoprotein cholesterol (LDL-C) as independent variables. Variables were added to the model until their inclusion was not significant (p>0.05), and the Bayesian information criterion (BIC) increased. Backward stepwise logistic regression was performed to verify the results and ensure consistency. Receiver operating characteristic (ROC) curve analysis and cross validation (CV) were performed.

**Results:** Data for 170 patients remained after exclusion of missing data and outliers. The optimal model contained the variables age, LDL-C, and triglycerides. The regression equation for this model was: Probability of FH causing variant = (0.74768 x LDL-C) – (0.06656 x age) – (1.26284 x triglycerides) – 0.06555. The area under the ROC curve (AUROC) for the model was 0.82, with an R^2^ of 0.25 and test error rate following CV of 0.25.

**Conclusions:** The model displayed promising results, and shows potential for improving the targeting of genetic testing in patients suspected of having FH. Interestingly, no individual components of the DLCN criteria were retained in the optimal model. Further work is required to develop and validate the model.

**Keywords:** Familial hypercholesterolaemia, genetic testing, variant, modelling

#### 81. Development of an application to support the identification of patients with familial hypercholesterolaemia

##### Rachel O'Leary^1,2^, Samuel G. Urwin^2,3^, Clare Lendrem^2,3^, Ahai Luvai^4^, R. Dermot G. Neely^2^, A. Joy Allen^2^

###### ^1^Northern Medical Physics and Clinical Engineering, The Newcastle-upon-Tyne Hospitals NHS Foundation Trust, Newcastle-upon-Tyne, UK; ^2^The National Institute for Health Research Newcastle In Vitro Diagnostics Co-operative, Newcastle-upon-Tyne, UK; ^3^Translational and Clinical Research Institute, Faculty of Medical Sciences, Newcastle University, Newcastle-upon-Tyne, UK; ^4^Laboratory Medicine, The Newcastle-upon-Tyne Hospitals NHS Foundation Trust, Newcastle-upon-Tyne, UK

####### **Correspondence:** Samuel G. Urwin

**Background:** Familial hypercholesterolaemia (FH) is associated with the long-term elevation of cholesterol levels in the blood. According to guidance from the National Institute for Health and Care Excellence (NICE), FH is suspected if total cholesterol exceeds 7.5 and 9 mmol/L in people under and over 30 years of age, respectively. The use of these cut-offs may over diagnose older people whose cholesterol has risen due to lifestyle factors, and underdiagnose younger people who have not reached the threshold, but may be at risk.

We aimed to develop an interactive application which places a patient on a specific population-based cholesterol centile according to their age and sex to improve the identification of people at risk of FH.

**Methods:** Health Survey for England (HSE) data were obtained from NHS Digital covering seven years between 2003 and 2014. Data for age, sex, high-density lipoprotein cholesterol (HDL-C), total cholesterol (TC), and use of lipid-lowering drugs were extracted. Centiles were derived at intervals of 0.1 between 0.5 and 99.5, for non-HDL cholesterol (non-HDL-C) [non-HDL-C = TC – HDL-C] and TC, in patients not being treated with lipid-lowering drugs.

**Results:** An interactive application was developed using Shiny that places a patient on a specific cholesterol centile based on their age and sex. Figure 1 shows an example of a 35 (I) and 55 (II) year old male with a TC of 9 mmol/L, which places the example patients on the 98.7 and 96.9 centiles, respectively.

**Fig. 1 (abstract 81).**
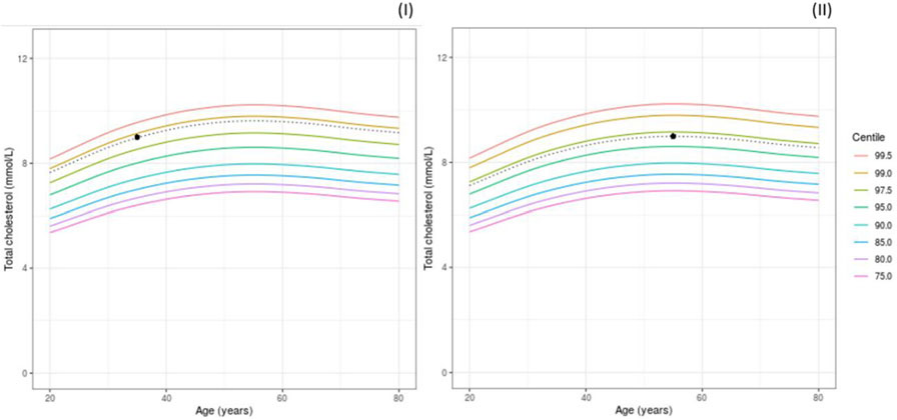
Screenshots from the interactive Shiny application (https://micncltools.shinyapps.io/miccentilesshinyapp/) showing total cholesterol centile plots for a 35 (I) and 55 (II) year old male with a total cholesterol of 9 mmol/L. The two example patients are each indicated by a black dot on the plots.

**Conclusions:** When used in conjunction with current methods, the use of age and sex adjusted cholesterol centiles could help improve the identification of patients with FH, and therefore refine the selection of index cases for targeted genetic testing.

**Keywords:** Familial hypercholesterolaemia, cholesterol, identification, application.

#### 82. The potential of ‘conceptually oriented’ pre-processing of covariates to improve prognostic models. A case study in low back pain patients

##### Anne Molgaard Nielsen^1^, Adrian Binding^2^, Casey Ahlbrandt-Rains^3^, Martin Boeker^3^, Stefan Feuerriegel^2^, Werner Vach^4,5^

###### ^1^Department of Sports Science and Clinical Biomechanics, University of Southern Denmark, Campusvej 55, DK-5230 Odense M, Denmark; ^2^Department of Management, Technology, and Economics, ETH Zurich, Weinbergstr. 56/58, 8092 Zurich, Switzerland; ^3^ Institute of Medical Biometry and Statistics, Faculty of Medicine and Medical Center, University of Freiburg, Stefan-Meier-Str. 26, D-79104 Freiburg i. Br., Germany; ^4^ Department of Orthopaedics and Traumatology, University Hospital Basel, Spitalstr. 21, CH-4031 Basel, Switzerland; ^5^Nordic Institute of Chiropractic and Clinical Biomechanics, Campusvej 55, DK-5230 Odense M, Denmark

####### **Correspondence:** Werner Vach

**Background:** A conceptually oriented pre-processing of a large number of potential prognostic factors may improve the development of a prognostic model and hence may play an important role in this process. However, it is unclear, whether this assumption holds and which way of pre-processing is optimal.

This study investigated whether various forms of conceptually oriented pre-processing or the preselection of established factors was superior to using all factors as input.

**Methods:** We made use of an existing project which developed two conceptually oriented subgroupings of low back-pain patients without taking the outcome into account. Based on the prediction of six outcome variables by seven statistical methods, this type of pre-processing was compared with domain specific principal component scores, medical experts’ preselection of established factors as well as with using all 112 available baseline factors.

**Results:** Subgrouping of patients was associated with low prognostic capacity. Applying a Lasso-based variable selection to all factors or to domain-specific principal component scores performed best. The preselection of established factors showed a good compromise between model complexity and prognostic capacity.

**Conclusions:** The prognostic capacity is hard to improve by means of a conceptually oriented pre-processing when compared to purely statistical approaches. However, a careful selection of already established factors combined in a simple linear model should be considered as one option when constructing a new prognostic rule based on a large number of potential prognostic factors.

**Keywords:** Model construction, prognostic models, domain knowledge

#### 83. Visualizing the results of a diagnostic accuracy study using comparison regions

##### Maren Eckert, Werner Vach

###### Institute of Medical Biometry and Statistics, Faculty of Medicine and Medical Center, University of Freiburg, Stefan-Meier-Str. 26, D-79104 Freiburg, Germany

####### **Correspondence:** Werner Vach

**Background:** The results of a diagnostic accuracy are often two parameters which we have to interpret together: Sensitivity and specificity, false positive and true negative rate, positive and negative predictive value, test positive rate and sensitivity, change in false positive and true negative rate, change in sensitivity and specificity, etc. For the interpretation, we often assign weights or utilities to each rate, and consider a weighted average. However, different stakeholders may use different weights, and the weights may also vary with the intended application of the test. This raises the question how we should present the results of a diagnostic accuracy study – and in particular their uncertainty – such that we can evaluate different weights in a post hoc situation.

**Methods:** Post hoc analyses of weighted averages require testing null hypotheses of the type that a weighted average is below a certain threshold. This can be approached by comparing the corresponding half space in the two-dimensional parameter space with a 95% confidence region. However, this is a very conservative approach.

**Results:** We present as an alternative approach so-called comparison regions, such that no overlap between the half space and the comparison region is equivalent to rejecting the null hypothesis at the 5% level.^[1]^ This way we can test any hypothesis about any weighted average, and in addition any hypothesis, which corresponds to the complement of a convex sets. Figure 1 illustrates the point.

**Fig. 1 (abstract 83).**
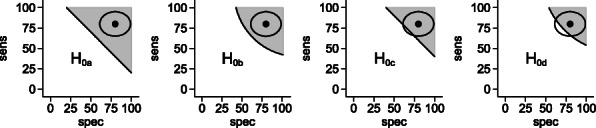
The results of a single arm diagnostic accuracy study visualized by the point estimate and a comparison region. Four different hypotheses are tested post hoc. H0a nad H0b can be rejected. H0c and H0d cannot be rejected.

**Conclusions:** The results of a diagnostic accuracy study can be presented in a way, which allows post hoc testing of (linear) hypotheses of weighted averages about two diagnostic accuracy parameters.

**Keywords:** Diagnostic accuracy studies, uncertainty, visualization, sensitivity and specificity, false positive and true negative rate.

**References**

^[1]^ M. Eckert, W. Vach, On the use of comparison regions in visualizing stochastic uncertainty in some two-parameter estimation problems. Biometrical Journal. 2019 (to appear). https://doi.org/10.1002/bimj.201800232

#### 84. Using harmonised results of different tests for a single biomarker in test accuracy meta-analysis

##### Yasaman Vali^1^, Jenny Lee^1^, Patrick M. Bossuyt^1^, Jerome Boursier^2,3^, Mohammad Hadi Zafarmand^1^, on behalf of the LITMUS project team

###### ^1^Department of Clinical Epidemiology, Biostatistics & Bioinformatics, Amsterdam UMC, Amsterdam, The Netherlands; ^2^Hepato-Gastroenterology Department, Angers University Hospital, Angers, France; ^3^HIFIH Laboratory, UPRES EA3859, Angers University, Angers, France

####### **Correspondence:** Yasaman Vali

**Background:** Evaluating the performance of a biomarker is challenging when different tests exist for measuring the same marker. Along with other sources of heterogeneity in systematic reviews of diagnostic test accuracy (DTA) studies, this can further influence and confound the results of meta-analysis.

We here propose a strategy to combine multiple tests to measure the same marker in a single meta-analysis. We apply this strategy to a meta-analysis of DTA studies of the Enhanced Liver Fibrosis (ELF) test, used in non-alcoholic fatty liver disease patients.

**Methods:** Our systematic search in five databases identified ten studies. Two different ELF tests were proposed, each using a different formula, expressed on a different scale. We initially conducted two meta-analyses, accounting for the multiple thresholds (diagmeta package in R). We then (1) evaluated, in a separate study of 502 samples, the presence of a linear relationship between the results of the tests. We (2) used the regression equation to obtain harmonized test results and (3) performed a single meta-analysis, combining the results from all nine studies.

**Results:** Eight studies used one formula (Siemens) and two used another (Guha). The first meta-analysis of the eight studies resulted in an “optimal” threshold (maximum Youden) of 9.30, for a sensitivity of 0.75 (95%CI 0.59; 0.87) and a specificity of 0.81 (95%CI 0.68; 0.90). After checking the linearity (R2: 0.995) and mapping the results on the same scale (Figure 1A), a meta-analysis of all ten studies was possible. This resulted in an “optimal” threshold of 9.38 for a sensitivity of 0.72 (95%CI 0.70; 0.90) and a specificity of 0.79 (95%CI 0.66; 0.88) (Figure 1B).

**Conclusions:** Our three-step method allows the combination of multiple tests of the same marker in a single meta-analysis, facilitating the interpretation of the accuracy of using specific thresholds.

**Fig. 1 (abstract 85).**
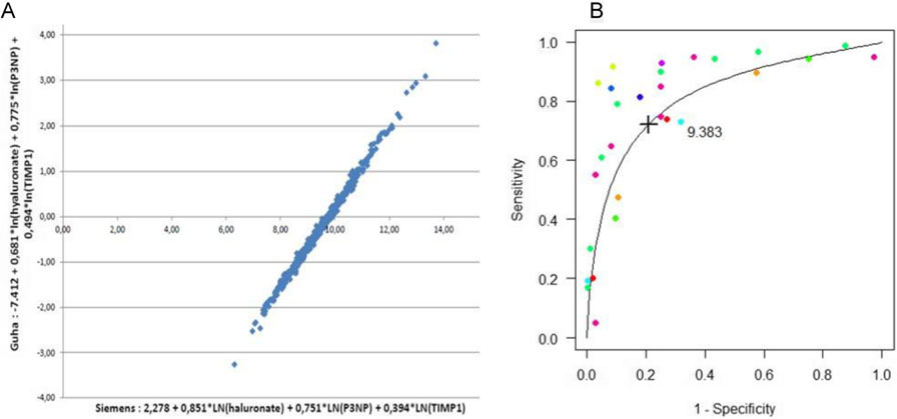
(A) Scatter plot of the correlation between two tests: Test 1: Siemens and Test 2: Guha. Using the regression formula of : Guha results = 0.8854*(Siemens results) - 8.6498. (B) Multiple thresholds sROC (mtsROC) curve based on the multiple thresholds model using homogenized thresholds. Circles represent information on sensitivity and specificity.

**Keywords:** Meta-analysis, accuracy studies, harmonization

#### 85. Large-scale validation of the Prediction model Risk Of Bias ASsessment Tool (PROBAST) using a short form

##### Esmee Venema^1,2^, Benjamin S Wessler^*3,4*^, Jessica K Paulus^3^, Rehab Salah^5^, Gowri Raman^6^, Lester Y Leung^7^, Benjamin C Koethe^*3*^, Jason Nelson^3^, Jinny G Park^3^, David van Klaveren^1,3^, Ewout W Steyerberg^*1,8*^, and David M Kent^3^

###### ^1^Department of Public Health, Erasmus MC University Medical Center, Rotterdam,The Netherlands; ^2^Department of Neurology, Erasmus MC University Medical Center, Rotterdam, The Netherlands; ^3^Predictive Analytics and Comparative Effectiveness Center, Tufts Medical Center, Boston, MA, USA ^4^Division of Cardiology, Tufts Medical Center, Boston, MA, USA; ^5^Benha Faculty of Medicine, Benha, Egypt; ^6^Center for Clinical Evidence Synthesis, Institute for Clinical Research and Health Policy Studies, Tufts Medical Center, Boston, MA, USA; ^7^Division of Stroke and Cerebrovascular Diseases, Department of Neurology, Tufts Medical Center, Boston, MA, USA; ^8^Department of Biomedical Data Sciences, Leiden University Medical Center, Leiden, The Netherlands

####### **Correspondence:** Esmee Venema

**Background:** The comprehensive Prediction model Risk Of Bias ASsessment Tool (PROBAST) ^[1]^ was developed for reviews of clinical prediction models (CPMs).

We aimed to assess whether PROBAST can identify CPMs that perform poorly at external validation and to develop a short form that is equally capable to identify poorly performing CPMs.

**Methods:** We evaluated risk of bias (ROB) using the PROBAST on 102 CPMs from the Tufts Predictive Analytics and Comparative Effectiveness Registry, compared to a short form consisting of six of the 20 PROBAST items anticipated to best identify high ROB. We then applied the short form to all CPMs in the Registry with at least 1 validation (n=556). Primary outcome was the change in the area under the receiver operating characteristic curve (dAUC, available for 1,147 validations) between the derivation and the validation cohorts in low versus high ROB CPMs.

**Results:** The full PROBAST classified 98 of 102 CPMS as high ROB. The short form identified 96 of these 98 as high ROB (98% sensitivity), with perfect specificity. Perfect agreement with the full PROBAST could be achieved with re-review of only a small number of low ROB CPMs. In the full CPM registry, 529 of 556 CPMs (95%) were classified as high ROB, 20 (4%) low ROB, and 7 (1%) unclear ROB. The median change in discrimination was significantly smaller in low ROB models (dAUC -0.9%, IQR -6.2% to 4.2%) compared to high ROB models (dAUC -12%, IQR -33% to 2.6%; p<0.001).

**Conclusions:** High ROB is pervasive among published CPMs. It is associated with poor performance at validation, supporting the application of PROBAST or a shorter version in reviews of CPMs.

**Keywords:** Prediction models, risk of bias, evaluation

**References**

^[1]^ R.F. Wolff, K.G.M. Moons, R.D. Riley, et al., *Ann Intern Med*, 170 2019, 51-58.

#### 86. Network meta-analysis methods for ranking the accuracy of multiple diagnostic tests

##### Areti Angeliki Veroniki^1,2,3^, Sofia Tsokani^1^, Yemisi Takwoingi^4,5^, Dimitris Mavridis^1,6^

###### ^1^Department of Primary Education, School of Education, University of Ioannina, Ioannina, Greece; ^2^Knowledge Translation Program, Li Ka Shing Knowledge Institute, St. Michael’s Hospital, Toronto, Ontario, Canada; ^3^Institute of Reproductive and Developmental Biology, Department of Surgery & Cancer, Faculty of Medicine, Imperial College, London, UK; ^4^Test Evaluation Research Group, Institute of Applied Health Research, University of Birmingham, UK; ^5^NIHR Birmingham Biomedical Research Centre, University Hospitals Birmingham NHS Foundation Trust and University of Birmingham, Birmingham, UK; ^6^Paris Descartes University, Sorbonne Paris Cité, Faculté de Médecine, Paris, France

####### **Correspondence:** Areti Angeliki Veroniki

**Background**: The diagnosis of a clinical condition is usually the first and more crucial step before initiating treatment. Diagnostic tests are routinely used for confirming or excluding a target condition. Although most diagnostic test accuracy (DTA) studies have focused on assessing a single index test, increasingly studies and systematic reviews are comparing the accuracy of multiple index tests to facilitate the selection of the best performing test(s) for patient care. For example, HPV DNA, HPV mRNA, and co-testing (Pap test + HPV DNA or mRNA test) can be used for cervical cancer diagnosis. But which test is the best? Since studies that directly compare test accuracy are not always available and comparisons between multiple tests constitute a network, DTA network meta-analysis (DTA-NMA) has been proposed.

We aimed to identify and assess DTA-NMA methods for comparing the accuracy of multiple diagnostic tests.

**Methods**: We conducted a methodological review of statistical and empirical studies that performed, described, or evaluated a DTA-NMA of at least 3 diagnostic tests. We searched PubMed, JSTOR, and Web of Science. Studies of any design published in English were eligible for inclusion. We also included relevant unpublished material.

**Results**: We included 38 relevant studies. The results will be presented at the Symposium. In particular, we will present the approaches that have been proposed together with a critique of their strengths and limitations. In addition, using cervical cancer as a case study, we will present an application of DTA-NMA methods to determine the most promising test (in terms of sensitivity and specificity) for use as the primary screening test for cervical cancer and to identify which women need referral for colposcopy.

**Conclusions**: Statistical approaches for comparative DTA meta-analysis of multiple tests differ and may influence interpretation and decision-making.

**Keywords:** Network meta-analysis, diagnostic test, accuracy, indirect comparison

#### 87. **Clinical Prediction Models for Patients with Acute Coronary Syndromes: Results from Independent External Validations**

##### **Benjamin S Wessler**^**1,2**^**, Jason Nelson**^**1**^**, Jinny Park**^**1**^**, Hannah McGinnis**^**1**^**, Jenica Upshaw**^**1,2**^**, Ben Van Calster**^**3**^**, David van Klaveren**^**1,4**^**, Ewout Steyerberg**^**4**^**, David Kent**^**1**^

###### ^1^Predictive Analytics and Comparative Effectiveness (PACE), Tufts Medical Center, USA; ^2^Division of Cardiology, Tufts Medical Center, Boston, MA, USA; ^3^KU Leuven, Department of Development and Regeneration, Leuven, Belgium; ^4^Department of Biomedical Data Sciences, Leiden University Medical Centre, Leiden, The Netherlands

####### **Correspondence: Benjamin S Wessler**

**Background:** It is increasingly recognized that clinical prediction models (CPMs) often do not perform as expected when they are tested on new databases. Independent external validations of CPMs are recommended but often not performed.

Here we conduct independent external validations of acute coronary syndrome (ACS) CPMs.

**Methods:** A systematic review identified CPMs predicting outcomes for patients with ACS. Independent external validations were performed by evaluating model performance using individual patient data from 5 large clinical trials. CPM performance with and without various recalibration techniques was evaluated with a focus on CPM discrimination (c-statistic, % relative change in c-statistic) as well as calibration (Harrell’s E_avg_, E_90_, net benefit).

**Results:** Of 276 ACS CPMs screened, 23 (8.3%) were compatible with the trials and 28 clinically appropriate external validations were performed. The median c statistic of the CPMs in the derivation cohorts was 0.76 (IQR, 0.74-0.78). The median c-statistic in these external validations was 0.70 (0.66-0.71) reflecting a 24% decrement in discrimination. Most of this decrement was due to narrower case-mix in the validation cohorts compared to derivation cohorts, as the median model based c-statistic was 0.71 (0.67-0.75). The median calibration slope in external validations was 0.82 (0.72- 0.95) and the median E_avg_ (standardized by the outcome rate) was 0.4 (0.3-0.8). Decision curve analysis indicates that most models had a high risk of causing net harm when not recalibrated, particularly if the decision threshold is not near the overall outcome rate. (Table 1)

**Conclusion:** For ACS CPMs, independent external validations generally demonstrate that discrimination is relatively preserved once case mix is taken into account. Since calibration is often poor, applying ‘off-the-shelf’ CPMs often risks net harm unless models are recalibrated.

**Table 1 (abstract 87).**
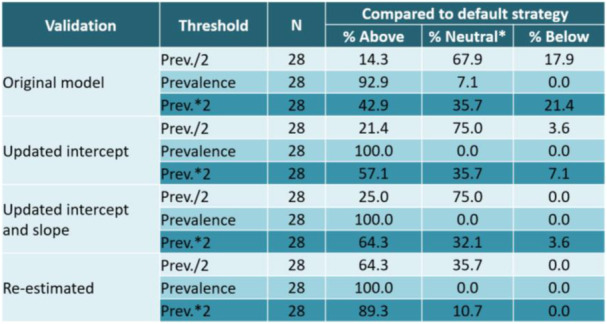
Effects of updating on net benefit. Threshold is the decision threshold and is represented in relation to the outcome prevalence. N is number of independent external validations. % Above refers to net benefit above the default strategy, % neutral refers to net benefit not different from the default strategy and % Below refers to net benefit less than the default strategy (net harm).

**Keywords:** Acute Coronary Syndrome, clinical prediction models, external validation

#### 88. Survey for elucidating potential roles for sepsis diagnostics in the UK NHS

##### Amanda Winter^1,2^, Raffaele Filieri^3^, Anthony Rostron^4,5^, Sara Graziadio^1,2^

###### ^1^National Institute for Health Research Newcastle In vitro Diagnostics Co-operative, Newcastle University, Medical School, Framlington Place, Newcastle-upon-Tyne, UK; ^2^The Newcastle-upon-Tyne Hospitals NHS Foundation Trust, Royal Victoria Infirmary, Queen Victoria Road, Newcastle-upon-Tyne, UK; ^3^Department of Marketing, Audencia Business School, 8 Route de la Jonelière, B.P. 31222, 44312 Nantes, Cedex 3, France; ^4^ Translational and Clinical Research Institute, Newcastle University, Medical School, Framlington Place, Newcastle-upon-Tyne, UK; ^5^South Tyneside and Sunderland NHS Foundation Trust, Kayll Road, Sunderland, UK

####### **Correspondence:** Amanda Winter

**Background:** Development of diagnostics is best driven by a comprehensive understanding of the clinical need and optimal role of the device within a care pathway.

We aimed to identify the potential roles of new diagnostic tests to inform future development objectives.

**Methods:** A survey was sent to UK NHS doctors and nurses who were involved in the care of patients with suspected sepsis. Questions focused on current care pathways in sepsis, current availability and utility of tests for infection and the unmet clinical needs in this pathway.

**Results:** Responses were received from 265 individuals across 68 NHS Trusts. The strongest role for a point of care (POC) sepsis test was as a ‘rule-out’ test which was favoured by doctors but not nursing staff, who preferred a ‘rule-in’ test. 67% of respondents indicated that the major cause of delay in caring for suspected sepsis patients was initial identification and flagging of deterioration. Existing blood tests did not greatly increase the confidence of consultants diagnosing sepsis. The majority of those surveyed felt there was a role for a POC sepsis test as they felt it would be quicker.

**Conclusions:** There is a need for sepsis diagnostics which are quicker and more specific than existing tests, to inform early identification and management of sepsis patients. Development of sepsis diagnostics should focus on solving these needs.

**Keywords:** Survey, development, diagnostic, care pathway, sepsis, point of care

